# Terrestrial forcing of marine biodiversification

**DOI:** 10.1038/s41598-022-12384-1

**Published:** 2022-05-18

**Authors:** Ronald E. Martin, Andrés L. Cárdenas

**Affiliations:** 1 Department of Earth Sciences, University of Delaware, Newark, DE 19716 USA; 2 Escuela de Ciencias Aplicadas e Ingeniería, Universidad EAFIT, Medellín, Colombia

**Keywords:** Evolution, Biogeochemistry, Solid Earth sciences

## Abstract

The diversification of the three major marine faunas during the Phanerozoic was intimately coupled to the evolution of the biogeochemical cycles of carbon and nutrients via nutrient runoff from land and the diversification of phosphorus-rich phytoplankton. Nutrient input to the oceans has previously been demonstrated to have occurred in response to orogeny and fueling marine diversification. Although volcanism has typically been associated with extinction, the eruption of continental Large Igneous Provinces (LIPs) is also a very significant, but previously overlooked, source of phosphorus involved in the diversification of the marine biosphere. We demonstrate that phosphorus input to the oceans peaked repeatedly following the eruption and weathering of LIPs, stimulating the diversification of nutrient-rich calcareous and siliceous phytoplankton at the base of marine food webs that in turn helped fuel diversification at higher levels. These developments were likely furthered by the evolution of terrestrial floras. Results for the Meso-Cenozoic hold implications for the Paleozoic Era. Early-to-middle Paleozoic diversity was, in contrast to the Meso-Cenozoic, limited by nutrient-poor phytoplankton resulting from less frequent tectonism and poorly-developed terrestrial floras. Nutrient runoff and primary productivity during the Permo-Carboniferous likely increased, based on widespread orogeny, the spread of deeper-rooting forests, the fossil record of phytoplankton, and biogeochemical indices. Our results suggest that marine biodiversity on geologic time scales is unbounded (unlimited), provided sufficient habitat, nutrients, and nutrient-rich phytoplankton are also available in optimal amounts and on optimal timescales.

## Introduction

**Figure 1:**
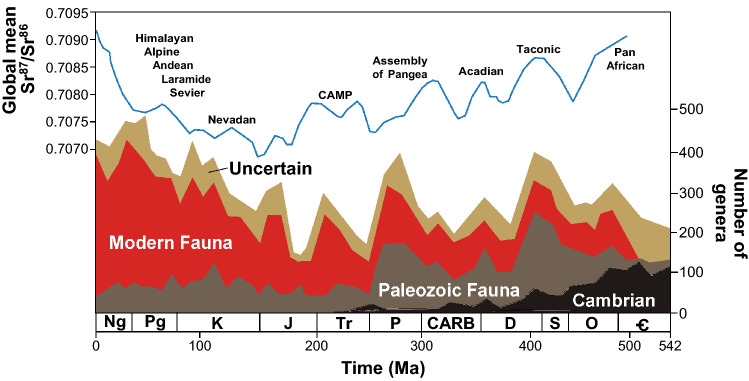
Marine biodiversity and major orogenic and tectonic phases during the Phanerozoic Eon. Genus-level richness (redrawn from 3*,* based on 2) with strontium isotope (^87^Sr/^86^Sr) curve (at 1 myr intervals^5^). Major orogenic episodes for the Phanerozoic are indicated. CAMP = Central Atlantic Magmatic Province is associated with the breakup of Pangea.

The geologic record of marine biodiversity consists of three main phases: the appearance and diversification of a relatively primitive Cambrian Fauna, the initial diversification of the Paleozoic Fauna and its subsequent plateauing (albeit with significant phases of diversification), and the nearly monotonic diversification of the Modern Fauna beginning in the Meso-Cenozoic era, as corroborated by recent studies utilizing the Paleobiology Database (PBDB; Fig. 1)^1–4^. A broad range of physico-chemical and biological factors, and likely acting and interacting on different scales of geologic time, have been hypothesized to explain the relative diversity of these three faunas, among them: sea level and habitat area associated with the tectonic forcing of sea level, changing climate regimes, latitudinal temperature gradients, biogeographic provinciality, oxygen levels, competition and predation, and biological disturbance^6–9^.

Physico-chemical controls are also associated with orogeny and erosion, potentially implicating nutrient runoff, primary productivity, and related “trophic resources” (food, energy) in marine biotic turnover and diversification during the Earth’s history^10–14^. Such relationships between nutrient availability, primary productivity, and biodiversity are evident in the broad patterns of strontium isotopes and the fossil record of marine biodiversity during the Phanerozoic^11–14^. The steep rise of the Modern fauna coincides with a similar, more-or-less monotonic rise of strontium isotope ratios (^87^Sr/^86^Sr), reflecting the widespread orogeny of this interval (Fig. 1). Major increases in ^87^Sr/^86^Sr indicate continental collisions of the Himalayan type: orogeny delivers the heavier ^87^Sr to the oceans in response to continental weathering, as opposed to ^86^Sr associated with increased rates of seafloor spreading and hydrothermal weathering^15,16^. Strontium isotope ratios (^87^Sr/^86^Sr) have therefore been employed as a general proxy for both terrestrial runoff and associated nutrient input to the oceans^11–17^. This behavior is affirmed by the 9‰ rise of ^87^Li in planktonic foraminifera from the Paleocene to present, consistent with uplift and more rapid continental denudation^18^. Based on ^87^Sr/^86^Sr ratios, phosphorus (as well as other nutrients such as silica) is typically considered to be initially derived from land by post-orogenic weathering by CO_2_ of continental rocks, especially those of granitic composition. Trends for selenium, another indicator of oxidative weathering of continental rocks, tend to parallel strontium isotope trends , but correlate more strongly with trace elements such as copper, molybdenum, and cadmium, some of which are necessary for the photosynthetic machinery of phytoplankton, and also with the macronutrient phosphorus^19,20^. Phosphorus in particular is critical to the synthesis of cell membrane phospholipids, nucleic acids (DNA, RNA), and bone, and trophic groups belonging to higher levels of the pelagic food web are reported to grow increasingly nutrient and especially phosphorus-rich^21^.

Continental rocks of granitic composition are not, however, the only source of phosphorus; so too, is volcanism, which has typically been associated with extinction in the fossil record. The associated injection of CO_2_ and its acceleration of the hydrologic cycle via warming and weathering of volcanic rocks, as well as the input of volcanic ash have also been determined to be significant sources of nutrients on ecologic (natural and field experiments) and geologic scales of time^22–29^, and especially of mafic-to-intermediate volcanics and pyroclastics like those associated with Large Igneous Provinces (LIPs) and continental arcs^30–33^. Basaltic igneous rocks are reported to weather 5–10 times faster than granitic or gneissic material while mantle plume volcanism is estimated to contribute ~ 10% of the total outgassed CO_2_ flux during LIP emplacement, continental rifting from 20 to 70%, and volcanic arcs 10–30% to CO_2_^30–34^. However, phosphorus has an ecologically long residence time in the oceans (~ 50,000 yrs) and has no atmospheric source, so its total oceanic inventory is ultimately controlled by weathering, nutrient runoff, uptake into living and dead biomass, recycling, and authigenic precipitation during early diagenesis of organic matter in sediment pore water. Total phosphorus inventory is thus considered the ultimate limiting nutrient, setting the upper limit for the roles of both nutrients and marine primary productivity in biodiversification on geologic time scales^35–37^.

Significant correlations of the time series between biodiversification, LIP emplacement and the weathering of volcanic rocks, and geochemical indices should therefore occur, given that they likely interacted through biogeochemical cycles linking the major Earth systems of land, ocean, atmosphere, and biosphere.

## Methods

We assessed the roles of phosphorus availability, primary productivity, and nutrient recycling on rates of nannofossil and marine genera origination rates (GOR) for the last 159.5 Ma (Jurassic, ca. late Oxfordian) of the Meso-Cenozoic. Detailed records of phosphorus accumulation rates (PAR), calcareous nannoplankton, isotope data, and genera origination rates are available for this interval. Data was compiled from a range of sources, which used various time scales and bins (see Supplementary Table S1 and Supplementary Fig. S1 for their raw data with their originally assigned ages).

Besides strontium, detailed records of two other stable isotopes are also available for the Meso-Cenozoic: carbon (δ^13^C) and sulfur (δ^34^S). Both carbon and sulfur cycles control redox conditions at the Earth’s surface by acting in a reciprocal manner. Positive carbon isotope ratios (δ^13^C) indicate enhanced marine and/or terrestrial photosynthesis, whereas negative ratios indicate decreased photosynthesis and/or input of isotopically-lighter ^12^C from various sources such as the erosion and oxidation of terrestrial organic carbon and its input into the oceans. High positive sulfur isotope values (δ^34^S) are interpreted to indicate extensive sulfate (SO_4_^2−^) reduction by sulfate-reducing bacteria, which are intolerant of oxygen and use dissolved SO_4_^2−^ as an electron acceptor to oxidize organic matter under anoxic conditions. Conversely, negative δ^34^S values are interpreted to indicate lower rates of sulfate reduction.

Our analyses were conducted using previously-published 11-myr binned data for stable isotopes and GOR; we further establishied 5-myr bins at the approximate mid-points of 11-myr bins in an attempt to achieve finer temporal resolution within the temporal constraints of the raw data.

Data used by Cárdenas and Harries^12^ were initially binned by them to 5-myr intervals using Linear Interpolation, including Genera Origination Rate (GOR) data of Alroy^38^ reported by him in approximately 11-myr bins. All data were then re-binned to 11-myr bins by Cardenas and Harries^12^, mirroring those used in the calculation of origination rates by Alroy^38^, prior to undertaking their statistical analyses. Materials sources are indicated below.

1. Strontium isotope ratios (‰)^12^: Data originally from McArthur et al.^39^ and linearly interpolated by Cárdenas and Harries^12^ in even 5-myr intervals recalibrated by them to the GTS2004 time scale^40^, and then re-binned to approximate 5-myr intervals by Cárdenas and Harries^12^.
2. CO_2_ (ppm^41^): Compiled from various sources and reported at various time intervals. Ages updated to GTS2012 time scale^42^.
3. Average Phosphorus Accumulation Rates (PAR, mg·cm^2^-ka^1^)^43^: Reported at 0.5 myr intervals beginning at 0.5 Ma based on a global data base of Deep Sea Drilling Project (DSDP) and Ocean Drilling Project (ODP) cores. Ages were attributed to each measurement using recent biostratigraphic distribution charts and the age assignments of Harland et al.^44^ by Föllmi^43^.

**Figure 2:**
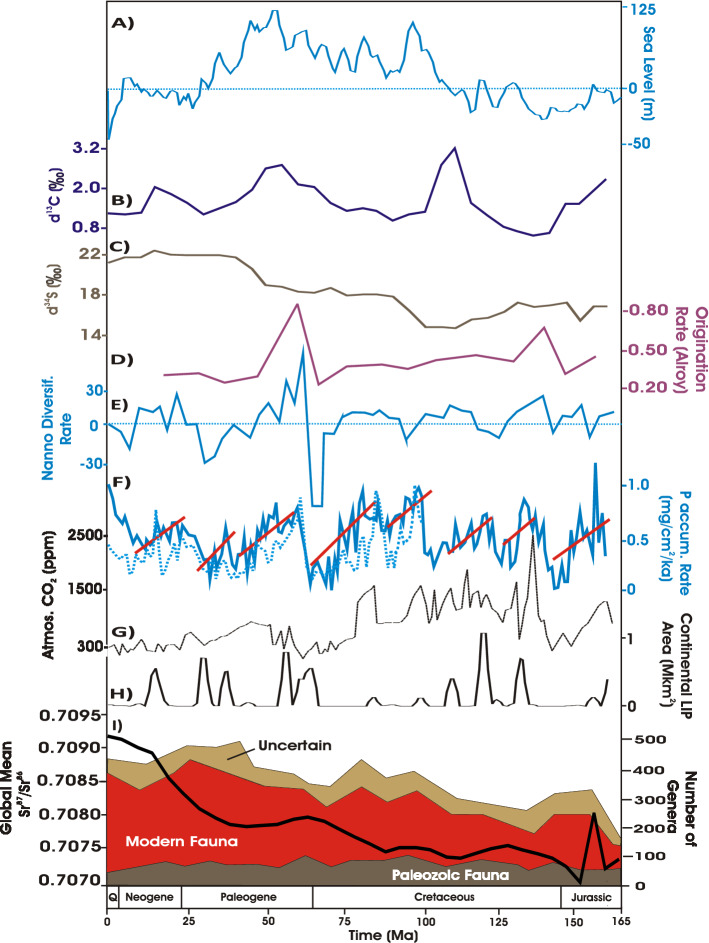
Physical, biogeochemical, and diversity indices for the Phanerozoic. (**A**) Sea level^47^. (**B**), δ^13^C^12^. (**C**) δ^34^S^12^. (**D**) Origination rate^12^. (**E**) Nannofossil diversification rate^41^. (**F**) Phosphorus accumulation rates (PAR)^43^. Solid line: total (biogenic + detrital) rate. Dashed line: biogenic rate only. Solid lines indicate generally declines in PAR and CO_2_ following Large Igneous Province (LIP) emplacement. Original PAR outlier value at 0.5 Ma removed for sake of scale (see Supplementary Figures and Tables). (**G**) Carbon dioxide levels^41^. (**H**), Area of continental Large Igneous Province (LIP) emplacement^34^. (**I**) Meso-Cenozoic genera biodiversity from Fig. 1 and strontium isotope ratios in approximate 5-myr bins^12^.

Two PAR time series were examined by us: Biogenic and Total (Biogenic + “Detrital”). Total PAR data extend back to 159.5 Ma (Jurassic, ca. late Oxfordian), Detrital to 102.5 Ma, and Biogenic to 100.5 Ma (Cretaceous, late Albian). The total P dataset includes information on phosphate phases that were originally dissolved and bioavailable and those that were detrital, i.e., derived from continental weathering without intermittent biological involvement. Föllmi attempted to discriminate between these two phases by considering a subset of pelagic biogenic sediments^43^. Phosphorus phases in this data subset were considered by him to likely be mainly nondetrital and representing reactive bioavailable phosphorus. The similarity of the total PAR and biogenic PAR curves (Fig. 2) indicates that changes in total P fluxes were closely tracked by changes in biogenic PAR fluxes or, perhaps more generally according to Föllmi, that changes in total continental weathering rates led to comparable changes in chemical weathering as the main long-term source of dissolved bioavailable phosphorus^43^.

4. Carbon isotope ratios (δ^13^C, ‰)^12^: Data originally from Veizer et al.^45^. Veizer et al. made carbon isotope measurements on calcitic and phosphatic shells, mainly brachiopods with some conodonts and belemnites, collected at high temporal resolution (up to 0.7 Ma or one biozone) from the stratotype sections of all continents but Antarctica and from many sedimentary basins, and mostly from paleotropical domains. Scanning electron microscopy, petrography, cathodoluminescence and trace element analyses of calcitic shells and the conodont alteration index (CAI) of the phosphatic shells indicated excellent preservation of their ultrastructure. These datasets were complemented by extensive literature compilations of Phanerozoic low-Mg calcitic, aragonitic and phosphatic isotope data of analogous skeletons. Data were treated by Cárdenas and Harries as for Sr isotope ratios^12^.
5. Sulfur isotope ratios (δ^34^S, ‰)^12^: Data originally from Kampschulte and Strauss in 5-myr intervals^46^. Sulfur isotopes were measured on biogenic calcite (brachiopods and belemnites) and micritic carbonates, all of Paleozoic and Mesozoic age. Results were supplemented with published sulfur isotope data for Neogene foraminifera, whole rock carbonates across the Cenomanian–Turonian boundary, and Cenozoic marine barites. Data were treated by Cárdenas and Harries as above^12^.
6. Sea-level (meters)^47^: Used by Cárdenas and Harries and originally reported on various durations based on GTS2004 time scale and re-binned by them^12,40^.
7. Calcareous nannoplankton diversification rates^48^: Available as graphs on 3-myr intervals and expressed as percentage increase or decrease of Rate of Speciation (R_s_) – Rate of Extinction (R_e_). Data were originally plotted as mid-points of intervals calibrated according to Berggren et al. and Gradstein et al.^49,50^. Peaks and valleys of Bown et al.’s Fig. 3 were digitized by us, resulting in data points spaced ~ 3 myr apart^51^.
8. Genera Origination Rates^12^: Data originally from Alroy^38^ on approximate 11-myr intervals based on GTS2004 time scale, then re-binned by Cárdenas and Harries to ~ 5-myr intervals, and re-binned by us to uniform 5-myr intervals^12,38,40^. Alroy originally binned marine origination rates using the global compilation of the Paleobiology Database into 48 intervals averaging 11 myr in duration and calibrated to the GTS2004 time scale^40^.
9. Large Igneous Provinces (LIP, km^2^)^34^: Data reported on 1-myr intervals and shown herein in Fig. 2. These data were not used in correlations because of numerous zero values (Fig. 2; Table 1).

**Table 1:** Comparison of correlations of uncorrected data*.

11-myr bin	CO_2_	^87^Sr/^86^Sr	Biogenic PAR	Total PAR	Nanno divers	δ^13^C	δ^34^S	GOR	Sea level
CO_2_		0.15795	0.6191	0.68079	**0.053748**	0.85201	0.2738	0.47378	0.18541
^87^Sr/^86^Sr	− 0.39868		0.33313	0.34889	0.7346	0.54963	0.23592	*0.0062*	0.76328
Biogenic PAR	− 0.2143	0.39522		**0.051711**	**0.089633**	0.35987	0.75203	*0.00724*	0.12364
Total PAR	0.12637	0.28295	**0.71429**		0.19441	0.66761	0.78895	**0.067292**	*0.011225*
Nanno divers	** − 0.52527**	0.099671	**0.64286**	0.38462		0.39192	**0.058637**	**0.0586**	0.493
δ^13^C	0.054945	0.17498	0.38095	0.13187	0.24835		0.74794	0.2207	**0.056154**
δ^34^S	0.31429	0.33888	− 0.1429	− 0.08242	** − 0.51648**	− 0.09451		0.9465	0.44563
GOR	− 0.20879	*0.69105*	*0.88095*	**0.52198**	**0.51648**	0.34945	0.01978		0.20256
Sea level	0.37582	0.088596	0.59524	*0.67582*	0.2	**0.52088**	0.22198	0.36264	

5-myr bin	CO_2_	^87^Sr/^86^Sr	Biogenic PAR	Total PAR	Nanno divers	δ^13^C	δ^34^S	GOR	Sea level
CO_2_		0.092295	0.46446	0.53194	*0.026636*	0.5898	0.35741	0.10304	**0.050857**
^87^Sr/^86^Sr	− 0.31841		0.13407	0.46623	0.63687	0.6026	0.36151	*0.004704*	0.56479
Biogenic PAR	− 0.19706	0.39118		*0.002854*	*0.023537*	0.3218	0.38036	*8.82E − 07*	0.1919
Total PAR	− 0.12576	0.14639	*0.69412*		*0.002661*	0.77617	0.63872	*0.000116*	*0.046842*
Nanno divers	** − ** *0.41133*	0.091503	*0.56176*	*0.55495*		**0.084763**	**0.054651**	*0.000106*	0.4535
δ^13^C	0.10443	0.10088	0.26471	0.057387	**0.32562**		*0.025269*	**0.076607**	*0.035143*
δ^34^S	0.17734	0.17585	− 0.2353	0.094628	** − 0.36059**	** − ** *0.41478*		0.94742	0.6075
GOR	− 0.30887	*0.51005*	*0.91176*	*0.67399*	*0.65764*	**0.33399**	− 0.01281		0.53119
Sea level	0.36601	− 0.11148	0.34412	*0.38584*	0.14483	*0.39261*	− 0.0995	0.12118	

*Data not corrected with Bonferroni correction (see Methods). Upper half of each binning method (above diagonally-arranged blank boxes): *p* (uncorrelated); lower half: Spearman’s correlation coefficient (ρ).

Italics: significant correlations (*p* < 0.05); boldface: marginal correlations discussed in text.

### Statistical analysis

We first conducted statistical analyses in Paleontological Analysis Statistical Software (PAST) version 4.03 and reaffirmed the results in R^52,53^. Linear Interpolation was used by us to re-bin all original data to 5-myr intervals approximately midway between 11-myr bins intervals in an attempt to achieve greater temporal resolution while remaining within the temporal constraints of the raw data. In several cases for data not taken from Cárdenas and Harries^12^, we readjusted the youngest ages to exactly 17 Ma so as to obtain exactly uniform 11-myr bins and from which we produced uniform 5-myr bins via linear interpolation. Readjusted ages (see below for original sources) are for: CO_2_ (originally 17.5 Ma), Phosphorus Accumulation Rate (PAR, total and biogenic; originally 17.5 Ma) and nannofossil diversification rates (originally 17.788 Ma; Supplementary Tables S2-S3; Supplementary Figs. S2, S3).

We first tested for the normal distribution of the undifferenced 11-myr and 5-myr binned data sets (Normality Tests option under PAST Univariate menu), one or more tests of which (Shapiro–Wilk, Anderson–Darling, χ^2^, and Jacque-Bera) indicated that the undifferenced 11-myr binned GOR were not normally-distributed (*p* < 0.05 for non-normality) and the p value of the δ^34^S approached that of non-normality (*p* < 0.05). One or more the same tests indicated that the undifferenced 5-myr binned CO_2_, Sr, δ^34^S, GOR, and sea level data were also non-normally distributed. Pearson’s r therefore proved inapplicable for correlation.

We therefore correlated using Spearman’s correlation coefficient (ρ), which is a non-parametric test that rank orders interval data when it is non-normally distributed. Spearman’s ρ has less sensitivity (power, 1-β, the likelihood of accepting the null hypothesis when it should have been rejected) than does Spearman’s r; the higher a statistical test’s power, the greater the probability that a small difference or correlation will be found to be significant. Unlike Pearson’s r, however, Spearman’s ρ makes no assumptions regarding the parameter mean or standard deviation of the sampled population. We used a significance level of *p* < 0.05. We also noted certain correlations which were considered marginally insignificant (0.05 > *p* < 0.1); these particular correlations may reflect the binning of the original time series, which exhibited different scales of temporal resolution.

Individual time series varied slightly in length; consequently, correlations were conducted only as far back in age as comparisons with the PAR time series permitted: ~ 159 Ma (Supplementary Fig.S4). Data were differenced to eliminate possible spurious correlations using the Evaluation Expression (u-d) of the Transform Data option of PAST (i.e., the value of each interval was subtracted from the one succeeding it in time; Supplementary Tables S4, S5).

We correlated both 11- and 5-myr binned uncorrected data and the same data corrected with the Bonferroni procedure, which has been advocated by some but not all investigators in the natural sciences. Because of these contrasting views, we correlated with and without the Bonferroni correction for the sake of comparison of our results.

The Bonferroni correction has been widely recommended when conducting multiple comparisons to cull null hypotheses which should be rejected. The Bonferroni correction is based on the equation *p* (corrected) = α/m, where α = desired uncorrected p at the outset (e.g., 0.05 in our study) and m = number of comparisons (“hypotheses” to be tested). The correction has therefore also been severely criticized, as it is extremely conservative. This is because as the number of variables used in multiple comparisons increases, the required p value with the Bonferroni correction for the rejection of the null hypothesis becomes increasingly small to the point that it may begin to produce false negatives (Type I error: null hypothesis rejection when the hypothesis is true) rather than omitting false positives (Type II error: null hypothesis acceptance when the hypothesis is false)^54–56^. Some workers therefore advocate using only uncorrected data^54–56^. The theoretical basis for advocating an adjustment for multiple comparisons is that “chance” serves as the first-order explanation for observed phenomena. This particular assertion is thought by some workers to undermine the basic premises of empirical research: that nature obeys regular laws that can be studied through observation; omitting the Bonferroni adjustment is therefore thought to be preferable because it will lead to fewer errors of interpretation when the data under evaluation are not randomly distributed but actual observations.

## Results

Both 5- and 11-myr binned uncorrected data yielded a significant Spearman’s correlation (*p* < 0.05) between ^87^Sr/^86^Sr and GOR, corroborating the role of nutrient runoff in GOR, as previously reported for the entire Phanerozoic (Table 1)^12^. Strong significant correlations for both bin intervals were also found between biogenic PAR and GOR, directly implicating the bioavailability of phosphorus and its transfer along food chains in GOR.

The increased temporal resolution of five-myr binned uncorrected data further yielded significant positive correlations: biogenic PAR with both total PAR and nannofossil diversification; total PAR with GOR; and nannofossil diversification with GOR; a significant negative correlation between δ^13^C and δ^34^S was also found. These particular correlations for 5-myr binned data were found to be either insignificant (*p* > 0.1) or, suggestively, marginally insignificant (*p* > 0.05 but < 0.1) for 11-myr binned data. Other marginally insignificant correlations were also found: negative correlations between δ^34^S and nannofossil diversification for both 5- and 11-myr binned data, and positive correlations for 5-myr binned data between δ^13^C and both nannofossil diversification (*p* < 0.085) and GOR (*p* < 0.077).

Sea level exhibited no impact on GOR with either 11 or 5-myr binned uncorrected data, similar to earlier findings for 11-myr time bins (Table 1)^12^. Both binning methods, however, implicated sea level in total PAR and carbon burial (δ^13^C) at significant to marginally insignificant levels. Sea level also exhibited a positive correlation with CO_2_ in 5-myr binned data, which was only very marginally insignificant (ρ = 0.37, *p* < 0.508).

We conducted a second set of correlations using the Bonferroni correction (see Methods). We chose to eliminate CO_2_ and sea level because they did not correlate with GOR using uncorrected data; this allowed us to further test the strength of correlations which had previously yielded significant correlations with uncorrected 11- and 5-myr binned data (Table 1). Elimination of CO_2_ and sea level resulted in a somewhat higher p value of 0.0024 (*p* = 0.05/21 comparisons). No significant correlations were found for 11-myr binned corrected data, whereas five-myr corrected data again yielded significant correlations between ^87^Sr/^86^Sr, biogenic PAR, total PAR, and nannofossil diversification with GOR (Table 2). These correlations therefore appear robust, given the previous results for 5-myr binned, uncorrected data (cf. Tables 1, 2).

**Table 2:** Comparison of correlations omitting CO_2_ and sea level using Bonferroni correction*.

11-myr bin	^87^Sr/^86^Sr	Biogenic PAR	Total PAR	Nanno divers	δ^13^C	δ^34^S	GOR
^87^Sr/^86^Sr		1	1	1	1	1	0.13025
Biogenic PAR	0.39522		1	1	1	1	0.15208
Total PAR	0.28295	0.71429		1	1	1	1
Nanno Divers	0.099671	0.64286	0.38462		1	1	1
δ^13^C	0.17498	0.38095	0.13187	0.24835		1	1
δ^34^S	0.33888	− 0.1429	− 0.0824	− 0.5165	− 0.0945		1
GOR	0.69105	0.88095	0.52198	0.51648	0.34945	0.01978	

5-myr bin	^87^Sr/^86^Sr	Biogenic PAR	Total PAR	Nanno Divers	δ^13^C	δ^34^S	GOR
^87^Sr/^86^Sr		1	1	1	1	1.00E + 00	*9.88E − 02*
Biogenic PAR	0.39118		0.059928	0.49428	1	1	*1.85E − 05*
Total PAR	0.14639	0.69412		0.055883	1	1	*0.002437*
Nanno Divers	0.091503	0.56176	0.55495		1	1	*0.002225*
δ^13^C	0.10088	0.26471	0.057387	0.32562		0.53065	1
δ^34^S	0.17585	− 0.2353	0.094628	− 0.36059	− 0.4148		1
GOR	*0.51005*	*0.91176*	*0.67399*	*0.65764*	0.33399	− 0.01281	

*Bonferroni correction set to p < 0.0024 (see Methods). Upper half of each binning method (above diagonally-arranged blank boxes): *p* (uncorrelated); lower half: Spearman’s correlation coefficient (ρ). Italics: significant correlations (*p* < 0.0024).

Cross-correlations of lagged indices against GOR weakened Spearman’s correlations of both uncorrected and corrected data to insignificance, however, suggesting that the interactions of environmental forcings and the responses of biodiversification to them are occurring primarily on relatively geologically-short time scales at least down to the range of 5–11 myr. The temporal spacing of data points of some time series did not permit us to further refine the time range without the questionable interpolation of further data points between more widely-spaced intervals.

## Discussion

### Meso-Cenozoic phytoplankton stoichiometry fueled diversification of the modern fauna

The dominant eukaryotic phytoplankton of the Meso-Cenozoic have been allied with so-called “red lineages” (coccolithophorids, dinoflagellates diatoms). These taxa are characterized by the accessory pigment chlorophyll c, specific trace elements employed in their plastids (such as those mentioned above), and low carbon:phosphorus (C:P) ratios^20,37^. Although their stoichiometry varies in response to natural conditions of temperature and nutrient availability, culture studies of modern representatives have been determined to be relatively phosphorus-rich and carbon-poor^20,37^. Modeling of C:P ratios occurring at the time of each major taxon’s appearance in the geologic record resembles that of their cultured modern representatives, suggesting that the nutrient preferences and stoichiometric compositions of modern representatives are evolutionarily conserved and reflect ancestral conditions rather than modern ones^20,37^. Ecologic stoichiometric theory predicts that increasing the phosphorus content of phytoplankton decreases the amount of energy that consumers must expend to respire excess carbon to obtain inorganic macronutrients like phosphorus; this potentially leaves excess resources like energy to be devoted to metabolic activity, reproduction, and changes in life cycles that could potentially impact diversification^21^.

The appearance of different major taxa of phytoplankton significantly impacted the deposition of deep-sea oozes and the carbon cycle. The advent of deep-sea calcareous oozes with the appearance of calcareous nannoplankton linked tectonism to atmospheric CO_2_ concentrations and weathering rates. A higher recycling efficiency between subducted carbon and CO_2_ return flux to the atmosphere via volcanism, as occurred during the Meso-Cenozoic in the form of calcareous oozes, would have provided positive feedback on volcanism and CO_2_ flux to the atmosphere, enhancing weathering rates, nutrient input to the oceans, and the primary productivity of calcareous phytoplankton^34,57,58^. Based on the fossil record and sedimentary markers, the apparent steep expansion of the Modern Fauna during the Cenozoic era was paralleled by the tremendous expansion of diatoms which are especially phosphorus-rich (via luxury storage), more so than calcareous nannoplankton^14,20,59–62^*.* The rain of phosphorus-rich dead organic matter likely promoted phosphorus bio-limitation in the marine realm. These trends are accompanied by a similar rise of both biogenic and total PAR in DSDP and ODP cores; total PAR may also partly reflect authigenic phosphorus precipitation during the last 15 myr of the Neogene (Fig. 2)^63^.

The expansion of diatoms during the Cenozoic may have been aided by the evolution and diversification of terrestrial angiosperm floras, as gymnosperm and calcareous phytoplankton diversity began to decline^60–62,64,65^. Angiosperm leaf litter, especially that of woody deciduous species, tends to be relatively nutrient and phosphorus-rich and decay relatively rapidly as compared to that of gymnosperms^66–68^. Diatoms increase their proportions of total phytoplankton biomass and primary production whenever silica is not limiting; grasses, which are silica-rich, became widespread during the last half of the Cenozoic in response to decreased atmospheric moisture resulting from late Eocene-Oligocene glaciation, and are considered to have increased silica input to the oceans via their highly soluble phytoliths^61,62^.

Rates of weathering are nevertheless geologically slow, limiting rates of nutrient runoff to the oceans. Nutrient cycling therefore likely remained critical to continued marine diversification and the biogeochemical cycles of phosphorus. This is especially true for the Meso-Cenozoic, as evidenced by increasing rates and depths of bioturbation^11–14,69^. Rapid decay of dead organic matter and nutrient recycling via sulfate reduction resulting from primary productivity and the secondary productivity of GOR is suggested by the near mirror-image relation between δ^34^S and δ^13^C and their negative correlation (Fig. 2; Table 1). Rates of weathering appear to have varied, however, on shorter time scales. Biogenic PAR peaked in association with initial LIP emplacement and peak CO_2_ concentrations, but they began to decline almost immediately during the succeeding ~30 myr, as weathering inexorably slowed in response to CO_2_ drawdown (Fig. 2).

### Implications for Paleozoic diversity

We have concentrated on the Meso-Cenozoic because of its more precise chronologies and completeness of the stratigraphic record, especially that of deep-sea cores. Our results nevertheless hold implications for Paleozoic marine biodiversity: we hypothesize that Paleozoic marine biodiversity remained relatively subdued precisely for the same reasons that it rose so dramatically during the Meso-Cenozoic. In terms of ecological theory, marine biodiversity of the Meso-Cenozoic was “unbounded” (unlimited) whereas that of the Paleozoic, although variable, was much more subdued, or “bounded” because of nutrient limitation^70,71^.

In contrast to the relatively continuous and widespread orogeny beginning in the Mesozoic, and especially during the Cenozoic, the early-to-middle Paleozoic portion of the diversity curve was punctuated by widely-separated peaks associated with peak strontium isotope ratios and orogeny (Fig. 1). Strontium isotope ratios are significantly greater for the Meso-Cenozoic than for the Paleozoic (Mann-Whitney U, *p*<0.0001, one-tailed), as are primary productivity and sulfate reduction, presumably in response to greater nutrient runoff (both Mann-Whitney U, *p*<0.0268, one-tailed; data from^12^). The eruption of continental LIPs is also thought to have been less frequent during the Paleozoic^5,34^. Although the accuracy of the LIP record before ~200 Ma is limited by the much greater duration of time available for erosion, Paleozoic LIPs are still recognizable by such features as dike swarms, and phosphorus input has been implicated in Late Ordovician marine biotic turnover^23,29^. The earlier Paleozoic was also characterized by terrestrial floras consisting of relatively primitive, rootless and shallow-rooting taxa. The litter of modern representatives of these taxa decays relatively slowly^64–68^.

These developments were again paralleled by the phytoplankton. Acritarchs were the dominant phytoplankton of the early-to-middle Paleozoic according to the fossil record and represent the organic-walled fraction of the phytoplankton preserved as cysts (resting stages resistant to inimical conditions). Acritarchs have been allied with eukaryotic “green” phytoplankton lineages. Presumed modern representatives of acritarchs are characterized by plastid trace elements which differ from those of red lineages, and by high C:P ratios (low phosphorus content, carbon-rich) that would have limited biodiversification according to ecologic stoichiometric theory^20,21^.

The Permo-Carboniferous appears to have been transitional between the earlier Paleozoic and the Mesozoic in terms of nutrient availability, primary productivity, and the diversification of marine biotas by increasing the ability of ecosystems to sustain higher but still optimal levels of diversity^72^. Acritarchs largely disappeared from the fossil record during the Permo-Carboniferous. Widespread orogenies associated with the formation of Pangea and the spread of deeper-rooting terrestrial floras inland are thought to have encouraged an increase of weathering rates and nutrient runoff, as indicated by strontium and selenium isotopes and phosphorus concentrations in shales, despite increased carbon and nutrient sequestration on land (Fig. 2)^19,73–75^. Acritarchs may actually represent pre-dinoflagellate lineages or perhaps even dinoflagellates themselves based on biomarkers, whereas modern dinoflagellates appear to be intermediate between red and green lineages with regard to C:P ratios and biomarkers^20,76^. As compared to nutrient-enrichment experiments in modern ecosystems and mass and minor extinctions, which may initially lower diversity via eutrophication, geologically-slow nutrient inputs maintain the relative stability of trophic resources in sufficient quantity thought critical to biodiversity and biodiversification^12–14,72,77,78^.

### Mechanisms of biodiversification

The exact pathways by which trophic resources are transmuted into biological diversity remain poorly-understood^79^. Nutrient availability and productivity nevertheless undoubtedly play significant roles in biodiversification on different time scales, like those associated with stoichometric theory: enhanced metabolism, increased grazing and predation, all of which would have impacted biogeochemical cycles; enhanced resources available for reproduction, population increase and potential dispersal leading to genetic isolation; and life cycle changes^8,9,13,21^. Productivity, which would influence oxygenation, total PAR, carbon burial and sulfate reduction, has been found to increase niche diversity more than area alone. Higher resource density may allow for increased specialization along a resource axis while still maintaining minimum viable population sizes^80^.

Area in the marine realm corresponds to sea level, which appears to be a function in part of tectonism, given its very nearly significant (but still marginally insignificant) correlation with CO_2_. Previous studies have found sea level to either be associated with biodiversity or not^12,81–83^. Comparison of the sea level curve with those of other indices (Fig. 2) indicates that the flooding of shelves undoubtedly plays significant roles in biodiversification akin to the temporal scales of tectonically-driven Sloss seismic sequences (ca. tens-of-millions of years or more) and longer tectonic cycles of ~250-300 myr duration (as indicated by the broad patterns of marine fossil biodiversity documented by previous studies)^81–84^. Similar to water depth in the open oceans beyond the shelf edge, sea level likely establishes the broad constraints of habitat availability and environmental conditions on primary and secondary productivity (δ^13^C) and microbial sulfate reduction (δ^34^S) via substrate, sediment accumulation rates, carbon and phosphorus burial, water column stratification and its oxygenation, depth and areal extent of the photic zone, and competition for light and nutrients^7,8,85^.

## Conclusion

The diversification of the Modern Fauna is coupled to the appearance and diversification of new and more phosphorus-rich phytoplankton taxa. The evolution of the major phytoplankton taxa of the Meso-Cenozoic in turn broadly parallels that of tectonism, the evolution of terrestrial floras, and the evolution of the biogeochemical cycles carbon and nutrients toward the present. Volcanism, long associated with extinction, serves as a rich source of phosphorus, especially with the eruption of mafic Large Igneous Provinces and the spread of angiosperms. The fossil and biogeochemical records for the Meso-Cenozoic suggest that enhanced nutrient availability and nutrient recycling relaxed the constraints of nutrient limitation, allowing marine diversity to remain more-or-less unbounded. In contrast, the Paleozoic was much more nutrient-limited overall, with the Permo-Carboniferous transitional between the early-to-middle Paleozoic and the Meso-Cenozoic based on phytoplankton and biogeochemical records.

## Supplementary Information

Supplementary Information.

## Data Availability

All data are available in the main text or the supplementary materials online.

## References

[1] Sepkoski JJ (1981). A factor analytic description of the Phanerozoic marine fossil record. Paleobiol..

[2] Sepkoski JJ (2002). A compendium of fossil marine animal genera. Bull. Amer. Paleontol..

[3] Alroy J (2010). The shifting balance of diversity among major marine animal groups. Science.

[4] Bush AM, Bambach RK (2015). Sustained Mesozoic-Cenozoic diversification of marine metazoa: a consistent signal from the fossil record. Geology.

[5] Prokoph A, Bilali HE, Ernst RE (2014). Periodicities in the emplacement of large igneous provinces through the Phanerozoic: relations to ocean chemistry and marine biodiversity evolution. Geosci. Front..

[6] Bambach RK (1999). Energetics in the global marine fauna: a connection between terrestrial diversification and change in the marine biosphere. Geobios.

[7] Bush AM, Bambach RK (2011). Paleoecologic megatrends in marine metazoa. Ann. Rev. Earth Planet. Sci..

[8] Vermeij GJ (2013). On escalation. Ann. Rev. Earth Planet. Sci..

[9] Bush AM, Payne JL (2021). Biotic and abiotic controls on the Phanerozoic history of marine animal biodiversity. Ann. Rev. Ecol. Evol. System..

[10] Bambach RK (1993). Seafood through time: changes in biomass, energetics, and productivity in the marine ecosystem. Paleobiology.

[11] Martin RE, Quigg A, Podkovyrov V (2008). Marine biodiversification in response to evolving phytoplankton stoichiometry. Palaeogeog. Paleoclimatol. Palaeoecol..

[12] Cárdenas AL, Harries PJ (2010). Effect of nutrient availability on marine origination rates throughout the Phanerozoic eon. Nat. Geosci..

[13] Allmon WD, Martin RE (2014). Seafood through time revisited: the Phanerozoic increase in marine trophic resources and its macroevolutionary consequences. Paleobiol..

[14] Martin RE, Servais T (2019). Review: Did the evolution of the phytoplankton fuel the diversification of the marine biosphere?. Lethaia.

[15] Edmond JM (1992). Himalayan tectonics, weathering processes, and the strontium isotope record in marine limestones. Science.

[16] Richter FM, Rowley DB, DePaolo DJ (1992). isotope evolution of seawater: the role of tectonics. Earth Planet. Sci. Lett..

[17] Tardy Y, N’Kounkou R, Probst JL (1989). The global water cycle and continental erosion during Phanerozoic time (570 my). Am. Jour. Sci..

[18] Misra S, Froelich PN (2012). Lithium isotope history of Cenozoic seawater: changes in silicate weathering and reverse weathering. Science.

[19] Large R, Halpin JA, Lounejeva E, Danyushevsky L, Maslennikov VV (2015). Cycles of nutrient trace elements in the Phanerozoic ocean. Gondwana Res..

[20] Quigg A, Finkel ZV, Irwin AJ, Rosenthal Y, Ho TY (2003). The evolutionary inheritance of elemental stoichiometry in marine phytoplankton. Nature.

[21] Sterner, R. W. & Elser, J. J. *Ecological Stoichiometry: The Biology of Elements from Molecules*. (Princeton University Press, 2002).

[22] Vermeij GJ (1995). Economics, volcanoes, and Phanerozoic revolutions. Paleobiology.

[23] Botting JP (2002). The role of pyroclastic volcanism in Ordovician diversification. Geol. Soc. Lond. Spec. Publ..

[24] Thingstad TF, Krom MD, Mantoura RFC, Flaten GAF, Groom S (2005). Nature of phosphorus limitation in the ultraoligotrophic eastern Mediterranean. Science.

[25] Duggen S, Croot P, Schacht U, Hoffmann L (2007). Subduction zone volcanic ash can fertilize the surface ocean and stimulate phytoplankton growth: evidence from biogeochemical experiments and satellite data. Geophys. Res. Lett..

[26] van Helmond NAGM, Sluijs A, Reichart GJ, Damsté JSS, Slomp CP (2014). A perturbed hydrological cycle during oceanic anoxic event 2. Geology.

[27] Shen J, Lei Y, Algeo TJ, Qinglai F, Servais T (2013). Volcanic effects on microplankton during the Permian-Triassic transition (Shangsi and Xinmin, south China). Palaios.

[28] Percival LME, Cohen AS, Davies MK, Dickson AJ, Hesselbo S (2016). Osmium isotope evidence for two pulses of increased continental weathering linked to Early Jurassic volcanism and climate change. Geology.

[29] Longman J, Mills BJW, Manners HR, Gernon TM, Palmer MR (2021). Late Ordovician climate change and extinctions driven by elevated volcanic nutrient supply. Nat. Geosci..

[30] Dessert C, Dupré B, Gaillardet J, François LM, Allègre CJ (2003). Basalt weathering laws and the impact of basalt weathering on the global carbon cycle. Chem. Geol..

[31] Milliman JD, Farnsworth KL (2011). River Discharge to the Coastal Ocean: A Global Synthesis.

[32] Hartmann J, Moosdorf N, Lauerwald R, Hinderer M, West AJ (2014). Global chemical weathering and associated P-release-the role of lithology, temperature and soil properties. Chem. Geol..

[33] Gernon TM, Hincks TK, Merdith AS, Rohling EJ, Palmer MR (2021). Global chemical weathering dominated by continental arcs since the mid-Paleozoic. Nat. Geosci..

[34] Johansson L, Zahirovic S, Müller RM (2018). The interplay between the eruption and weathering of large igneous provinces and the deep-time cycle. Geophys. Res. Lett..

[35] Tyrrell T (1999). The relative influences of nitrogen and phosphorus on oceanic primary production. Nature.

[36] Moore CM, Mills MM, Arrigo KR, Berman-Frank I, Bopp L (2013). Processes and patterns of oceanic nutrient limitation. Nat. Geosci..

[37] Sharoni S, Halevy I (2022). Geologic controls on phytoplankton elemental composition. Proc. Nat. Acad. Sci..

[38] Alroy J (2008). Dynamics of origination and extinction in the marine fossil record. Proc. Natl. Acad. Sci. U.S.A..

[39] McArthur JM, Howarth RJ, Bailey TR (2001). Strontium isotope stratigraphy: LOWESS version 3: Best fit to the marine Sr-isotope curve for 0-509 Ma and accompanying look-up table for deriving numerical age. Jour. Geol..

[40] Gradstein, F. M., Ogg, J. G. & Smith, A. G. Eds., *A Geologic Time Scale*, *2004* (Cambridge University Press, 2004).

[41] Foster GL, Royer DL, Lunt DJ (2017). Future climate forcing potentially without precedent in the last 20 million years. Nat. Comm..

[42] Gradstein, F. M., Ogg, J. G., Schmitz, M. D. & Ogg, G. M. Eds., *The Geologic Time Scale* (Elsevier, 2012).

[43] Föllmi KB (1995). 160 m.y. record of marine sedimentary phosphorus burial: coupling of climate and continental weathering under greenhouse and icehouse conditions. Geology.

[44] Harland, W. B., Armstrong, R. L., Cox, A. V., Craig, L. E., Smith, A. G. *et al*. *A Geological Timescale 1989* (Cambridge University Press, 1990).

[45] Veizer J, Davin A, Azmy K, Bruckschen P, Buhl D (1999). ^87^Sr/^86^Sr, δ^13^C and δ^18^O evolution of Phanerozoic seawater. Chem. Geol..

[46] Kampschulte A, Strauss H (2004). The sulfur isotopic evolution of Phanerozoic seawater based on the analysis of structurally substituted sulfate in carbonates. Chem. Geol..

[47] Miller KG, Kominz M, Browning JV, Wright JD, Mountain GS (2005). The Phanerozoic record of global sea-level change. Science.

[48] Bown, P. R., Lees, J. A. & Young, J. R. Calcareous nannoplankton diversity and evolution through time, in *Coccolithophores - From Molecular Processes to Global Impact* (eds H. Thierstein, H. & Young, J.) Chap. 18 (Springer, 2004).

[49] Berggren, W. A., Kent, D. V., Swisher III, C. C. &Aubry, M.-P., A revised Cenozoic chronology and chronostratigraphy in *Geochronology, Time-Scales, and Global Stratigraphic Correlation: Framework for an Historical Geology* (eds. Berggren, W. A., Kent, D. V. & Hardenbol, J.) Chap. 8 (Society for Sedimentary Geology, 1995).

[50] Gradstein, F. M., Agterberg, F. P., Ogg, J. G., Hardenbol, J., Van Veen, P. et al. A Triassic, Jurassic and Cretaceous time-scale, in *Geochronology, Time-Scales, and Global Stratigraphic Correlation:Framework for an Historical Geology* (eds W. A. Berggren, W. A., Kent, D. V. & Hardenbol, J.) Chap. 7 (Society for Sedimentary Geology, 1995)

[51] Rohatgi, A. C:\Users\Carol\Desktop\WebPlotDigitizer-4.2-win32-x64.

[52] Hammer, Ø. https://past.en.lo4d.com/windows.

[53] The R Project for Statistical Computing, www.r-project.org.

[54] Rothman KJ (1990). No adjustments are needed for multiple comparisons. Epidemiology.

[55] Perneger TV (1998). What is wrong with Bonferroni adjustments. Brit. Med. Jour..

[56] Cabin RJ, Mitchell RJ (2000). To Bonferroni or not to Bonferroni: when and how are the questions. Bull. Ecol. Soc. Am..

[57] Wong K, Mason E, Brune S, East M, Edmonds S (2019). Deep carbon cycling over the past 200 million years: a review of fluxes in different tectonic settings. Front. Earth Sci..

[58] Planck T, Manning CE (2019). Subducting carbon. Nature.

[59] Margalef R (1978). Life-forms of phytoplankton as survival alternatives in an unstable environment. Oceanol. Acta.

[60] Cermeño P (2016). The geological story of marine diatoms and the last generation of fossil fuels. Perspect. Phycol..

[61] Katz ME, Finkel ZE, Grzebyk D, Knoll AH, Falkowski PG (2004). Evolutionary trajectories and biogeochemical impacts of marine eukaryotic phytoplankton. Ann. Rev. Ecol. Evol. Syst..

[62] Katz O (2015). Silica phytoliths in angiosperms: phylogeny and early evolutionary history. New Phytol..

[63] Anderson LD, Delaney ML, Faul KL (2001). Carbon to phosphorus ratios in sediments: implications for nutrient cycling. Glob. Biogeochem. Cycles.

[64] Cleal CJ, Cascales-Miñana B (2014). Composition and dynamics of the great Phanerozoic evolutionary floras. Lethaia.

[65] Dahl TW, Arens SKM (2020). The impacts of land plant evolution on Earth's climate and oxygenation state an interdisciplinary review. Chem. Geol..

[66] Wright IJ, Reich PB, Ackerly DD, Baruch Z, Bongers F (2004). The worldwide leaf economics spectrum. Nature.

[67] Cornwell WK, Cornelissen JHC, Amatangelo K, Dorrepaal E, Eviner VT (2008). Plant species traits are the predominant control on litter decomposition rates within biomes worldwide. Ecol. Lett..

[68] Díaz S, Kattge J, Cornelissen JHC, Wright IJ, Lavore S (2016). The global spectrum of plant form and function. Nature.

[69] Thayer, C. W. Sediment‐mediated biological disturbance and the evolution of marine benthos. in *Biotic Interactions in Recent and Fossil Benthic Communities* (eds Tevesz, M.J.S. & McCall, P.L.) Chapter (Plenum, 1983).

[70] Rabosky DL, Hurlbert AH (2015). Species richness at continental scales is dominated by ecological limits. Am. Nat..

[71] Harmon LJ, Harrison S (2015). Species diversity is dynamic and unbounded at local and continental scales. Am. Nat..

[72] Rosenzweig, M L. & Abramsky, Z. How are diversity and productivity related? in *Species Diversity in Ecological Communities: Historical and Geographical Perspectives* (eds Ricklefs, R. E. & Schluter, D.) Chap. 5 (University of Chicago Press, 1993).

[73] Algeo TJ, Scheckler SE (1998). Terrestrial-marine teleconnections in the Devonian: links between the evolution of land plants, weathering processes, and marine anoxic events. Philos. Trans. R. Soc. Lond..

[74] Servais T, Martin RE, Nützel A (2016). The impact of the ‘terrestrialization process’ in the late Palaeozoic: pCO2, pO2, and the ‘phytoplankton blackout’. Rev. Palaeobot. Palynol..

[75] D’Antonio MP, Ibarra DE, Boyce CK (2020). Land plant evolution, decreased, rather than increased weathering rates. Geology.

[76] Moldowan JM, Talyzina NM (1998). Biogeochemical evidence for dinoflagellate ancestors in the Early Cambrian. Science.

[77] Martin, R.E. Catastrophic fluctuations in nutrient levels as an agent of mass extinction: upward scaling of ecological processes? in *Biodiversity Dynamics: Turnover of Populations, Taxa, and Communities* (eds McKinney, M.L. & Drake, J.A.) Chap. 17 (Columbia University Press, 1998).

[78] Algeo TJ, Chen ZQ, Fraiser ML, Twitchett RJ (2011). Terrestrial–marine teleconnections in the collapse and rebuilding of Early Triassic marine ecosystems. Palaeogeog. Palaeoclimatol. Palaeoecol..

[79] Antell GW, Saupe EE (2021). Bottom-up controls, ecological revolutions and diversification in the oceans through time. Curr. Biol..

[80] Hurlbert AH, Stegen JC (2014). When should species richness be energy limited, and how would we know?. Ecol. Lett..

[81] Valentine JW, Moores EM (1972). Global tectonics and the fossil record. J. Geol..

[82] Hannisdal B, Peter SE (2011). Phanerozoic earth system evolution and marine biodiversity. Science.

[83] Zaffos A, Finnegan S, Peters SE (2017). Plate tectonic regulation of global marine animal diversity. Proc. Nat. Acad. Sci..

[84] Roberts GG, Mannion PD (2019). Timing and periodicity of Phanerozoic marine biodiversity and environmental change. Nature.

[85] Martin RE (2003). The fossil record of biodiversity: nutrients, productivity, habitat area and differential preservation. Lethaia.

