# Supplementary material for: Terrestrial forcing of marine biodiversification
Source: Sci Rep. 2022 May 18;12:8309. doi: 10.1038/s41598-022-12384-1 (PMC9117300; doi:10.1038/s41598-022-12384-1)
Supplement: Supplementary file 1 — Supplementary Information. [file 41598_2022_12384_MOESM1_ESM.docx]

**Martin and Cárdénas-Supplementary Figures and Tables**

**(References refer to original article)**

**Fig. S1.** **| Raw data.** **a**, Last 160 Myr signatures of global mean CO_2_ (n=230)^41^. **b**, Mean global isotopic signature of ^87^Sr/^86^Sr (n=36)^12^. **c**, Total PAR (n=160)^43^. **d**, Mean global isotopic signature of δ^13^C (n=35)^12^. **e**, Mean global isotopic signature of δ^34^S (n=35)^12^. **f**, Biogenic PAR (n=100)^43^. **g**, Nannofossil diversification rate (n=57)^48^. **h**, Eustatic sea level (n=3095)^47^. **i**, GOR (n=16)^12^. Dots represent raw data for all curves.

**Fig. S2.** **Raw data binned into 11-myr bins.** **a**, Last 160 Myr signatures of global mean CO_2_ (n=230)^41^. **b**, Mean global isotopic signature of ^87^Sr/^86^Sr (n=36)^12^. **c**, Total PAR (n=160)^43^. **d**, Mean global isotopic signature of δ^13^C (n=35)^12^. **e**, Mean global isotopic signature of δ^34^S (n=350)^12^. **f**, Biogenic PAR (n=100)^43^. **g**, Nannofossil diversification rate (n=57)^48^. **h**, Eustatic sea level (n=3095)^47^. **i**, GOR (n=16)^12^. Dots represent raw data for all curves.

**Fig. S3.** **Raw data binned into 5-myr bins.** **a**, Last 160 Myr signatures of global mean CO_2_ (n=230)^41^. **b**, Mean global isotopic signature of ^87^Sr/^86^Sr (n=36)^12^. **c**, Total PAR (n=160)^43^. **d**, Mean global isotopic signature of δ^13^C (n=35)^12^. **e**, Mean global isotopic signature of δ^34^S (n=35)^12^. **f**, Biogenic PAR (n=100)^43^. **g**, Nannofossil diversification rate (n=57)^48^. **h**, Eustatic sea level (n=3095)^47^. **i**, GOR (n=16)^12^. Dots represent raw data for all curves.

**Fig. S4.** **Statistically significant relationships obtained between changes in both GOR and changes in the environmental signatures using 5-myr bins.** **a**, Correlation between first differences of GOR and ^87^Sr/^86^Sr. **b**, Correlation between first differences of GOR and biogenic PAR. **c**, Correlation between first differences of GOR and total PAR. **d**, Correlation between first differences of GOR and nannofossil diversification rate. **e**, Correlation between first differences of GOR and δ^13^C. For all the correlation tests carried out by the Spearman’s method, the time series consisted of 27 (GOR vs ^87^Sr/^86^Sr), 16 (GOR vs biogenic PAR), 27 (GOR vs total PAR), 29 (GOR vs nannofossil diversification rate), and 29 (GOR vs δ^13^C) points ordered in time, and the alpha level established at 0.05.

Supplementary Figure S1.

Supplementary Figure S2.

Supplementary Figure S3.

Supplementary Figure S4.

| **Supplementary Table S1. ALL RAW DATA WITH ORIGINAL AGES.*** | | | | | |  |  |
| --- | --- | --- | --- | --- | --- | --- | --- |
| **Age (Ma)*** | **CO2 (ppm) (*20*)** | **Age (Ma) (*8*)** | **Sr Ratio (‰) (*8*)** | **Age (Ma) (*19*)** | **TOTAL P Accum Rate (PAR) (mg٠cm^-2^ka^-1^) (*19*)** | **DETRITAL P Accum Rate (PAR) (mg٠cm^-2^ka^-1^) (*19*)** | **BIOGENIC P Accum Rate (PAR) (mg٠cm^-2^ka^-1^) (*19*)** |
| 1 | 244 | 0 | 0.70913 | 0.5 | 1.90437472 | 2.69960579 | 0.44865336 |
| 1.1 | 214 | 4.9 | 0.70908 | 1.5 | 0.94071485 | 1.31296766 | 0.39029337 |
| 1.2 | 335 | 9.9 | 0.70895 | 2.5 | 0.69228203 | 0.87235839 | 0.36111223 |
| 1.2 | 217 | 14.8 | 0.70887 | 3.5 | 0.63460613 | 0.82098129 | 0.319773 |
| 1.2 | 193 | 19.8 | 0.70849 | 4.5 | 0.55732621 | 0.72262622 | 0.30102477 |
| 1.3 | 141 | 24.8 | 0.70826 | 5.5 | 0.65381843 | 0.91410189 | 0.34249599 |
| 1.3 | 196 | 29.9 | 0.70802 | 6.5 | 0.52025258 | 0.62396653 | 0.34492547 |
| 1.3 | 157 | 34.9 | 0.70789 | 7.5 | 0.38006625 | 0.57137511 | 0.15240393 |
| 1.4 | 192 | 40 | 0.70779 | 8.5 | 0.35894576 | 0.47371277 | 0.18167277 |
| 1.4 | 192 | 45.1 | 0.70776 | 9.5 | 0.43896483 | 0.51027653 | 0.35138818 |
| 1.4 | 194 | 50.2 | 0.70777 | 10.5 | 0.34811445 | 0.38264154 | 0.28973289 |
| 1.4 | 194 | 55.3 | 0.70779 | 11.5 | 0.31898968 | 0.34243054 | 0.26499254 |
| 1.5 | 303 | 60.4 | 0.70787 | 12.5 | 0.37230719 | 0.35410871 | 0.40950062 |
| 1.5 | 239 | 65.5 | 0.7079 | 13.5 | 0.34805982 | 0.36068753 | 0.32932404 |
| 1.5 | 210 | 70.5 | 0.70783 | 14.5 | 0.37967754 | 0.39965533 | 0.34630489 |
| 1.6 | 230 | 75.4 | 0.70773 | 15.5 | 0.56185744 | 0.47653488 | 0.7966596 |
| 1.6 | 207 | 80.4 | 0.7076 | 16.5 | 0.43070819 | 0.44436425 | 0.39456822 |
| 1.6 | 247 | 85.4 | 0.7075 | 17.5 | 0.50177838 | 0.8198785 | 0.13751188 |
| 1.6 | 282 | 90.3 | 0.70737 | 18.5 | 0.45235384 | 0.77869251 | 0.19539999 |
| 1.7 | 239 | 95.3 | 0.70744 | 19.5 | 0.37312626 | 0.41932863 | 0.29543258 |
| 1.8 | 230 | 100.3 | 0.70743 | 20.5 | 0.56967808 | 0.7349163 | 0.34816624 |
| 1.9 | 252 | 105.3 | 0.7074 | 21.5 | 0.57777628 | 0.64315584 | 0.4662799 |
| 1.9 | 307 | 110.2 | 0.7073 | 22.5 | 0.39290748 | 0.61661102 | 0.2893874 |
| 2.1 | 300 | 115.2 | 0.70728 | 23.5 | 0.53433049 | 0.52439691 | 0.54252272 |
| 2.1 | 299 | 120.2 | 0.70736 | 24.5 | 0.49585885 | 0.48741699 | 0.49932274 |
| 2.1 | 278 | 125.1 | 0.70742 | 25.5 | 0.42918922 | 0.44114503 | 0.42333338 |
| 2.2 | 338 | 130.1 | 0.70747 | 26.5 | 0.42631514 | 0.42133086 | 0.42824783 |
| 2.3 | 342 | 135.1 | 0.70741 | 27.5 | 0.41641483 | 0.30554057 | 0.4409869 |
| 2.3 | 405 | 140 | 0.70734 | 28.5 | 0.35581516 | 0.31272347 | 0.36748569 |
| 2.3 | 387 | 145 | 0.70727 | 29.5 | 0.27984135 | 0.19380059 | 0.31629876 |
| 2.6 | 400 | 149.4 | 0.70715 | 30.5 | 0.25321009 | 0.27781469 | 0.21034521 |
| 2.6 | 385 | 153.7 | 0.70694 | 31.5 | 0.25659873 | 0.4096428 | 0.19155136 |
| 4 | 80 | 158.1 | 0.70796 | 32.5 | 0.16944161 | 0.16819778 | 0.17027594 |
| 5 | 324 | 162.4 | 0.70711 | 33.5 | 0.2606356 | 0.15628051 | 0.32254287 |
| 5.5 | 222 | 166.7 | 0.70726 | 34.5 | 0.21345717 | 0.24713285 | 0.11041345 |
| 6.4 | 213 |  |  | 35.5 | 0.24703418 | 0.29191095 | 0.21225308 |
| 6.7 | 283 |  |  | 36.5 | 0.29669592 | 0.31993027 | 0.27746359 |
| 6.8 | 372 |  |  | 37.5 | 0.47172862 | 0.84023877 | 0.28760572 |
| 7 | 148 |  |  | 38.5 | 0.35128581 | 0.84728904 | 0.16258707 |
| 7.1 | 468 |  |  | 39.5 | 0.22894727 | 0.37273735 | 0.12234651 |
| 7.7 | 292 |  |  | 40.5 | 0.21257413 | 0.28977188 | 0.15850609 |
| 8.4 | 133 |  |  | 41.5 | 0.28569941 | 0.58245018 | 0.10816063 |
| 8.8 | 274 |  |  | 42.5 | 0.27973541 | 0.43130099 | 0.17658848 |
| 9 | 204 |  |  | 43.5 | 0.41386617 | 0.60402377 | 0.17129329 |
| 9.1 | 197 |  |  | 44.5 | 0.17770483 | 0.12849069 | 0.21762885 |
| 10.6 | 448 |  |  | 45.5 | 0.32605011 | 0.40090091 | 0.26517445 |
| 12.7 | 267 |  |  | 46.5 | 0.24415824 | 0.30823784 | 0.18845634 |
| 12.8 | 273 |  |  | 47.5 | 0.38037838 | 0.28363807 | 0.4857639 |
| 13 | 296 |  |  | 48.5 | 0.39320677 | 0.37649885 | 0.42097271 |
| 13 | 176 |  |  | 49.5 | 0.58821929 | 0.65538057 | 0.40290181 |
| 13.1 | 370 |  |  | 50.5 | 0.44388324 | 0.46038694 | 0.38709299 |
| 13.7 | 133 |  |  | 51.5 | 0.63690957 | 0.80008337 | 0.27077732 |
| 13.7 | 101 |  |  | 52.5 | 0.62180975 | 1.07144074 | 0.2817948 |
| 13.8 | 190 |  |  | 53.5 | 0.87082429 | 0.9929651 | 0.47197004 |
| 14.4 | 132 |  |  | 54.5 | 0.45790427 | 0.44084607 | 0.48605339 |
| 14.7 | 74 |  |  | 55.5 | 0.45207299 | 0.47201958 | 0.41982007 |
| 15.1 | 182 |  |  | 56.5 | 0.54939509 | 0.57388407 | 0.48202172 |
| 15.6 | 473 |  |  | 57.5 | 0.58498143 | 0.61702449 | 0.53591139 |
| 16 | 333 |  |  | 58.5 | 0.53212479 | 0.50918913 | 0.5760436 |
| 16.1 | 289 |  |  | 59.5 | 0.6946494 | 0.75797952 | 0.6512664 |
| 17.5 | 261 |  |  | 60.5 | 0.78263022 | 0.8528667 | 0.68528815 |
| 17.8 | 237 |  |  | 61.5 | 0.5047315 | 0.37478281 | 0.91542318 |
| 18 | 160 |  |  | 62.5 | 0.36234881 | 0.3025971 | 0.59476792 |
| 18.2 | 274 |  |  | 63.5 | 0.25112978 | 0.25766353 | 0.23250591 |
| 21 | 312 |  |  | 64.5 | 0.15344846 | 0.15108436 | 0.15584955 |
| 23 | 203 |  |  | 65.5 | 0.21848156 | 0.2698288 | 0.11598297 |
| 25.6 | 588 |  |  | 66.5 | 0.23009595 | 0.29802262 | 0.17765143 |
| 31.6 | 377 |  |  | 67.5 | 0.24124032 | 0.31649859 | 0.16367791 |
| 31.6 | 265 |  |  | 68.5 | 0.19827808 | 0.25469048 | 0.17494668 |
| 34.2 | 399 |  |  | 69.5 | 0.15308094 | 0.28479446 | 0.0931607 |
| 45 | 780 |  |  | 70.5 | 0.27462328 | 0.31393412 | 0.23625093 |
| 50.5 | 684 |  |  | 71.5 | 0.12898436 | 0.15013186 | 0.1226192 |
| 50.7 | 397 |  |  | 72.5 | 0.23133484 | 0.18013127 | 0.26880183 |
| 50.9 | 178 |  |  | 73.5 | 0.19732396 | 0.15157735 | 0.21204083 |
| 51.1 | 725 |  |  | 74.5 | 0.61448313 | 1.0284914 | 0.30921154 |
| 51.1 | 1285 |  |  | 75.5 | 0.4656497 | 0.52152198 | 0.39803187 |
| 51.2 | 1354 |  |  | 76.5 | 0.31404532 | 0.33918769 | 0.27444716 |
| 51.4 | 971 |  |  | 77.5 | 0.56342219 | 0.70964077 | 0.28198117 |
| 51.5 | 1709 |  |  | 78.5 | 0.7080337 | 1.16250023 | 0.1950618 |
| 51.5 | 1299 |  |  | 79.5 | 0.27575399 | 0.29190647 | 0.21343872 |
| 51.8 | 903 |  |  | 80.5 | 0.86868093 | 1.02601607 | 0.47182696 |
| 51.8 | 1210 |  |  | 81.5 | 1.16511941 | 1.29300391 | 0.53351906 |
| 51.9 | 1260 |  |  | 82.5 | 0.670087 | 0.884352 | 0.22087945 |
| 52 | 301 |  |  | 83.5 | 0.63224167 | 0.7120191 | 0.48679303 |
| 52.6 | 750 |  |  | 84.5 | 0.64121572 | 0.69051451 | 0.51345009 |
| 52.8 | 400 |  |  | 85.5 | 0.59522717 | 0.53489106 | 0.9801607 |
| 53.5 | 80 |  |  | 86.5 | 0.81891061 | 0.78687358 | 0.8958441 |
| 54.2 | 814 |  |  | 87.5 | 0.48565736 | 0.39727403 | 0.72578763 |
| 54.9 | 231 |  |  | 88.5 | 0.47290833 | 0.47113889 | 0.47706291 |
| 55.5 | 328 |  |  | 89.5 | 0.59124257 | 0.66513188 | 0.2172992 |
| 55.5 | 100 |  |  | 90.5 | 0.43084755 | 0.45476873 | 0.39091568 |
| 56.2 | 461 |  |  | 91.5 | 0.60953424 | 0.54495003 | * |
| 56.5 | 487 |  |  | 92.5 | 0.66891586 | 0.66891586 | * |
| 56.5 | 428 |  |  | 93.5 | 0.6900539 | 0.69767996 | 0.63429048 |
| 56.5 | 100 |  |  | 94.5 | 0.57973546 | 0.76397456 | 0.31882838 |
| 56.7 | 579 |  |  | 95.5 | 0.97342273 | 1.06366635 | 0.30743289 |
| 57.3 | 816 |  |  | 96.5 | 0.93388696 | 0.97474939 | 0.37994648 |
| 57.7 | 474 |  |  | 97.5 | 0.75824017 | 0.78045034 | 0.67880118 |
| 60.7 | 0 |  |  | 98.5 | 1.14109536 | 1.03043027 | 1.61889651 |
| 63.6 | 235 |  |  | 99.5 | 1.21065849 | 1.27523413 | 0.94339581 |
| 63.7 | 212 |  |  | 100.5 | 1.01908839 | 1.06512535 | 0.70001365 |
| 64.6 | 200 |  |  | 101.5 | 0.28603593 | 0.24952912 |  |
| 64.7 | 289 |  |  | 102.5 | 0.2801754 | 0.2801754 |  |
| 64.8 | 400 |  |  | 103.5 | 0.30850293 |  |  |
| 64.9 | 175 |  |  | 104.5 | 0.22384808 |  |  |
| 65 | 357 |  |  | 105.5 | 0.26508482 |  |  |
| 65.1 | 278 |  |  | 106.5 | 0.31336164 |  |  |
| 65.2 | 163 |  |  | 107.5 | 0.36857173 |  |  |
| 65.2 | 360 |  |  | 108.5 | 0.36840373 |  |  |
| 65.2 | 178 |  |  | 109.5 | 0.30443555 |  |  |
| 65.3 | 340 |  |  | 110.5 | 0.30798027 |  |  |
| 65.4 | 88 |  |  | 111.5 | 0.57416412 |  |  |
| 65.4 | 298 |  |  | 112.5 | 0.39163277 |  |  |
| 65.6 | 111 |  |  | 113.5 | 0.32809594 |  |  |
| 65.8 | 178 |  |  | 114.5 | 0.36105536 |  |  |
| 65.9 | 54 |  |  | 115.5 | 0.45668994 |  |  |
| 65.9 | 300 |  |  | 116.5 | 0.55997235 |  |  |
| 66.1 | 183 |  |  | 117.5 | 0.32784569 |  |  |
| 66.2 | 277 |  |  | 118.5 | 0.35706633 |  |  |
| 66.4 | 90 |  |  | 119.5 | 0.45076054 |  |  |
| 66.5 | 0 |  |  | 120.5 | 0.66082228 |  |  |
| 66.5 | 492 |  |  | 121.5 | 0.68697872 |  |  |
| 66.5 | 42 |  |  | 122.5 | 0.41115521 |  |  |
| 66.6 | 263 |  |  | 123.5 | 0.56185042 |  |  |
| 66.6 | 15 |  |  | 124.5 | 0.30760394 |  |  |
| 66.7 | 453 |  |  | 125.5 | 0.26317222 |  |  |
| 66.9 | 265 |  |  | 126.5 | 0.23560384 |  |  |
| 66.9 | 426 |  |  | 127.5 | 0.39315859 |  |  |
| 67 | 128 |  |  | 128.5 | 0.38397373 |  |  |
| 67 | 219 |  |  | 129.5 | 0.2147084 |  |  |
| 67.2 | 300 |  |  | 130.5 | 0.23225541 |  |  |
| 67.2 | 560 |  |  | 131.5 | 0.31066868 |  |  |
| 68.3 | 175 |  |  | 132.5 | 0.70248458 |  |  |
| 68.5 | 10 |  |  | 133.5 | 0.9517736 |  |  |
| 68.7 | 171 |  |  | 134.5 | 0.8474753 |  |  |
| 68.8 | 110 |  |  | 135.5 | 0.59809864 |  |  |
| 69 | 158 |  |  | 136.5 | 0.52122358 |  |  |
| 69.1 | 138 |  |  | 137.5 | 0.69952161 |  |  |
| 69.1 | 60 |  |  | 138.5 | 0.39559396 |  |  |
| 69.1 | 252 |  |  | 139.5 | 0.54694889 |  |  |
| 69.1 | 172 |  |  | 140.5 | 0.28887722 |  |  |
| 69.1 | 328 |  |  | 141.5 | 0.20423645 |  |  |
| 69.3 | 202 |  |  | 142.5 | 0.21523434 |  |  |
| 69.4 | 145 |  |  | 143.5 | 0.13980852 |  |  |
| 69.5 | 345 |  |  | 144.5 | 0.14260395 |  |  |
| 69.7 | 204 |  |  | 145.5 | 0.20916018 |  |  |
| 69.9 | 309 |  |  | 146.5 | 0.14967613 |  |  |
| 70 | 375 |  |  | 147.5 | 0.15870355 |  |  |
| 70.1 | 329 |  |  | 148.5 | 0.47286229 |  |  |
| 70.2 | 122 |  |  | 149.5 | 0.31113939 |  |  |
| 70.3 | 38 |  |  | 150.5 | 0.4063561 |  |  |
| 70.5 | 126 |  |  | 151.5 | 0.76325954 |  |  |
| 71.4 | 254 |  |  | 152.5 | 0.42348855 |  |  |
| 71.5 | 289 |  |  | 153.5 | 0.48362262 |  |  |
| 71.6 | 294 |  |  | 154.5 | 0.35664753 |  |  |
| 77.1 | 480 |  |  | 155.5 | 0.27876807 |  |  |
| 77.9 | 1180 |  |  | 156.5 | 2.14039555 |  |  |
| 77.9 | 504 |  |  | 157.5 | 0.37769938 |  |  |
| 83 | 1522 |  |  | 158.5 | 0.77323231 |  |  |
| 83.3 | 740 |  |  | 159.5 | 0.28452984 |  |  |
| 92.2 | 772 |  |  |  |  |  |  |
| 95.3 | 1307 |  |  |  |  |  |  |
| 98 | 1520 |  |  |  |  |  |  |
| 100 | 746 |  |  |  |  |  |  |
| 100.5 | 1368 |  |  |  |  |  |  |
| 100.8 | 763 |  |  |  |  |  |  |
| 102 | 1428 |  |  |  |  |  |  |
| 102.4 | 621 |  |  |  |  |  |  |
| 102.5 | 886 |  |  |  |  |  |  |
| 103.1 | 792 |  |  |  |  |  |  |
| 103.3 | 1052 |  |  |  |  |  |  |
| 103.5 | 1060 |  |  |  |  |  |  |
| 104.6 | 1246 |  |  |  |  |  |  |
| 104.7 | 1139 |  |  |  |  |  |  |
| 104.8 | 1254 |  |  |  |  |  |  |
| 106.1 | 1399 |  |  |  |  |  |  |
| 106.4 | 1474 |  |  |  |  |  |  |
| 106.5 | 1679 |  |  |  |  |  |  |
| 106.6 | 1497 |  |  |  |  |  |  |
| 106.8 | 600 |  |  |  |  |  |  |
| 106.9 | 1496 |  |  |  |  |  |  |
| 107.8 | 1201 |  |  |  |  |  |  |
| 108.8 | 1015 |  |  |  |  |  |  |
| 110.3 | 1065 |  |  |  |  |  |  |
| 111.1 | 1637 |  |  |  |  |  |  |
| 111.5 | 996 |  |  |  |  |  |  |
| 111.5 | 1966 |  |  |  |  |  |  |
| 111.8 | 1880 |  |  |  |  |  |  |
| 112.3 | 1829 |  |  |  |  |  |  |
| 112.7 | 1419 |  |  |  |  |  |  |
| 113.4 | 591 |  |  |  |  |  |  |
| 113.5 | 1335 |  |  |  |  |  |  |
| 113.5 | 449 |  |  |  |  |  |  |
| 113.8 | 1295 |  |  |  |  |  |  |
| 114.8 | 1514 |  |  |  |  |  |  |
| 114.9 | 1426 |  |  |  |  |  |  |
| 115.3 | 1367 |  |  |  |  |  |  |
| 115.5 | 986 |  |  |  |  |  |  |
| 116 | 1071 |  |  |  |  |  |  |
| 116.1 | 1666 |  |  |  |  |  |  |
| 116.5 | 1190 |  |  |  |  |  |  |
| 117.8 | 1364 |  |  |  |  |  |  |
| 119.4 | 1179 |  |  |  |  |  |  |
| 119.7 | 1076 |  |  |  |  |  |  |
| 119.7 | 592 |  |  |  |  |  |  |
| 119.7 | 904 |  |  |  |  |  |  |
| 119.7 | 1084 |  |  |  |  |  |  |
| 123.3 | 736 |  |  |  |  |  |  |
| 123.6 | 607 |  |  |  |  |  |  |
| 123.7 | 824 |  |  |  |  |  |  |
| 123.8 | 1018 |  |  |  |  |  |  |
| 126.4 | 1065 |  |  |  |  |  |  |
| 126.6 | 1510 |  |  |  |  |  |  |
| 127.5 | 1704 |  |  |  |  |  |  |
| 127.9 | 393 |  |  |  |  |  |  |
| 129.8 | 940 |  |  |  |  |  |  |
| 130.1 | 112 |  |  |  |  |  |  |
| 130.1 | 1150 |  |  |  |  |  |  |
| 130.2 | 1023 |  |  |  |  |  |  |
| 130.8 | 842 |  |  |  |  |  |  |
| 131.3 | 1910 |  |  |  |  |  |  |
| 132 | 2562 |  |  |  |  |  |  |
| 134.3 | 653 |  |  |  |  |  |  |
| 135.7 | 924 |  |  |  |  |  |  |
| 137.9 | 174 |  |  |  |  |  |  |
| 142.2 | 560 |  |  |  |  |  |  |
| 143.5 | 232 |  |  |  |  |  |  |
| 154.7 | 1272 |  |  |  |  |  |  |
| 157.8 | 558 |  |  |  |  |  |  |

*Data sources indicated by reference numbers in parentheses in column headings. Some published time series may be longer than indicated here. We include only those portions overlapping with the Phosphorus Accumulation Rate (PAR) time series.

** **Blank spaces used in bin interpolation and differencing. Paleontological Statistical Analysis (PAST) version 4.03 accounts for missing data.

**Supplementary Table S1. continued.**

| **Time (Ma) (*18*)** | **Nannofossil Diversification Rate (Rs-Re٠ 3my^-1^) (*18*)** | **Age (Ma) (*8*)** | **δ^13^C (‰) (*8*)** | **δ^34^S (‰) (*8*)** | **Age (Ma) (*8*)** | **Genera Origination Rate (*8*)** | **Time (Ma) (Johansson et al.)** | **Continental LIP area (km^2^) (Johansson et al.)** |
| --- | --- | --- | --- | --- | --- | --- | --- | --- |
| 0 | -2.364 | 0 | 1.39 | 21.2 | 17.3 | 0.2542774 | 0 | 16752.8 |
| 3.121 | -5.867 | 4.9 | 1.39 | 21.8 | 28.5 | 0.270974 | 1 | 10623 |
| 5.929 | -16.725 | 9.9 | 1.43 | 21.8 | 37.1 | 0.1981283 | 2 | 18093.3 |
| 8.738 | 8.844 | 14.8 | 2.13 | 22.2 | 48.1 | 0.2448549 | 3 | 275.08 |
| 11.546 | 6.392 | 19.8 | 1.95 | 22.1 | 60.6 | 0.8084943 | 4 | 6668.34 |
| 11.546 | 6.392 | 24.8 | 1.69 | 22 | 68 | 0.184003 | 5 | 15978.1 |
| 14.979 | 9.194 | 29.9 | 1.39 | 22 | 77 | 0.323612 | 6 | 0 |
| 17.788 | -4.816 | 34.9 | 1.54 | 22 | 88.5 | 0.3351221 | 7 | 0 |
| 20.596 | 18.301 | 40 | 1.73 | 21.8 | 96.5 | 0.30547 | 8 | 0 |
| 23.717 | -0.613 | 45.1 | 2.07 | 20.6 | 105.8 | 0.3714468 | 9 | 30134.4 |
| 27.15 | -0.613 | 50.2 | 2.65 | 18.9 | 118.5 | 0.4112173 | 10 | 38942.2 |
| 29.646 | -24.431 | 55.3 | 2.73 | 18.7 | 130.7 | 0.3625163 | 11 | 8810.28 |
| 33.391 | -21.629 | 60.4 | 2.2 | 18.2 | 140.9 | 0.6259499 | 12 | 0 |
| 35.576 | -8.669 | 65.5 | 2.14 | 18.1 | 148.2 | 0.2627632 | 13 | 0 |
| 39.008 | -2.715 | 70.5 | 1.7 | 18.6 | 157.8 | 0.4001926 | 14 | 481756 |
| 42.129 | -5.166 | 75.4 | 1.47 | 17.8 |  |  | 15 | 481704 |
| 44.626 | -9.019 | 80.4 | 1.54 | 17.9 |  |  | 16 | 238384 |
| 47.746 | 4.991 | 85.4 | 1.47 | 17.9 |  |  | 17 | 166467 |
| 50.243 | 11.296 | 90.3 | 1.2 | 17.7 |  |  | 18 | 0 |
| 53.988 | 0.788 | 95.3 | 1.38 | 16.2 |  |  | 19 | 10239 |
| 56.796 | 26.007 | 100.3 | 1.46 | 14.5 |  |  | 20 | 10238.2 |
| 59.917 | 8.844 | 105.3 | 2.75 | 14.5 |  |  | 21 | 643.32 |
| 62.725 | 44.221 | 110.2 | 3.2 | 14.4 |  |  | 22 | 643.1 |
| 64.91 | -50.35 | 115.2 | 1.69 | 15.3 |  |  | 23 | 0 |
| 67.094 | -50.701 | 120.2 | 1.35 | 15.5 |  |  | 24 | 2295.59 |
| 68.967 | -6.918 | 125.1 | 1.03 | 16.1 |  |  | 25 | 2296.07 |
| 71.463 | -6.918 | 130.1 | 0.91 | 17.1 |  |  | 26 | 0 |
| 74.584 | 2.89 | 135.1 | 0.8 | 16.6 |  |  | 27 | 0 |
| 78.017 | 6.392 | 140 | 0.86 | 16.8 |  |  | 28 | 0 |
| 80.825 | 7.093 | 145 | 1.67 | 17.1 |  |  | 29 | 89981.5 |
| 83.634 | 4.991 | 149.4 | 1.68 | 15.2 |  |  | 30 | 717048 |
| 86.13 | 7.793 | 153.7 | 2.01 | 16.7 |  |  | 31 | 714851 |
| 89.251 | 3.24 | 158.1 | 2.35 | 16.7 |  |  | 32 | 84853.4 |
| 92.684 | 2.539 |  |  |  |  |  | 33 | 0 |
| 95.804 | -8.319 |  |  |  |  |  | 34 | 5045.82 |
| 98.925 | 0.088 |  |  |  |  |  | 35 | 5045.81 |
| 102.046 | 4.991 |  |  |  |  |  | 36 | 58.34 |
| 104.854 | 3.24 |  |  |  |  |  | 37 | 549567 |
| 107.663 | 2.539 |  |  |  |  |  | 38 | 549486 |
| 110.784 | 10.245 |  |  |  |  |  | 39 | 9748.41 |
| 113.28 | 7.443 |  |  |  |  |  | 40 | 9748.39 |
| 116.089 | 7.093 |  |  |  |  |  | 41 | 0 |
| 119.521 | -5.166 |  |  |  |  |  | 42 | 0 |
| 122.018 | -5.166 |  |  |  |  |  | 43 | 0 |
| 125.139 | -9.72 |  |  |  |  |  | 44 | 48186 |
| 128.571 | 1.138 |  |  |  |  |  | 45 | 74633.2 |
| 131.38 | 7.093 |  |  |  |  |  | 46 | 0 |
| 134.501 | 11.296 |  |  |  |  |  | 47 | 0 |
| 137.621 | 12.697 |  |  |  |  |  | 48 | 0 |
| 140.118 | 16.55 |  |  |  |  |  | 49 | 0 |
| 143.863 | -6.567 |  |  |  |  |  | 50 | 0 |
| 146.359 | 4.991 |  |  |  |  |  | 51 | 0 |
| 149.48 | 3.59 |  |  |  |  |  | 52 | 0 |
| 152.601 | 10.595 |  |  |  |  |  | 53 | 0 |
| 155.721 | -9.019 |  |  |  |  |  | 54 | 0 |
| 158.53 | 3.24 |  |  |  |  |  | 55 | 0 |
|  |  |  |  |  |  |  | 56 | 849213 |
|  |  |  |  |  |  |  | 57 | 849654 |
|  |  |  |  |  |  |  | 58 | 126.06 |
|  |  |  |  |  |  |  | 59 | 15504.7 |
|  |  |  |  |  |  |  | 60 | 15502.7 |
|  |  |  |  |  |  |  | 61 | 424431 |
|  |  |  |  |  |  |  | 62 | 424502 |
|  |  |  |  |  |  |  | 63 | 61.04 |
|  |  |  |  |  |  |  | 64 | 625879 |
|  |  |  |  |  |  |  | 65 | 569443 |
|  |  |  |  |  |  |  | 66 | 9753.4 |
|  |  |  |  |  |  |  | 67 | 0 |
|  |  |  |  |  |  |  | 68 | 0 |
|  |  |  |  |  |  |  | 69 | 0 |
|  |  |  |  |  |  |  | 70 | 0 |
|  |  |  |  |  |  |  | 71 | 0 |
|  |  |  |  |  |  |  | 72 | 0 |
|  |  |  |  |  |  |  | 73 | 0 |
|  |  |  |  |  |  |  | 74 | 0 |
|  |  |  |  |  |  |  | 75 | 0 |
|  |  |  |  |  |  |  | 76 | 0 |
|  |  |  |  |  |  |  | 77 | 0 |
|  |  |  |  |  |  |  | 78 | 0 |
|  |  |  |  |  |  |  | 79 | 0 |
|  |  |  |  |  |  |  | 80 | 0 |
|  |  |  |  |  |  |  | 81 | 0 |
|  |  |  |  |  |  |  | 82 | 0 |
|  |  |  |  |  |  |  | 83 | 0 |
|  |  |  |  |  |  |  | 84 | 141553 |
|  |  |  |  |  |  |  | 85 | 141525 |
|  |  |  |  |  |  |  | 86 | 0 |
|  |  |  |  |  |  |  | 87 | 10853.8 |
|  |  |  |  |  |  |  | 88 | 10850.3 |
|  |  |  |  |  |  |  | 89 | 0 |
|  |  |  |  |  |  |  | 90 | 25062.9 |
|  |  |  |  |  |  |  | 91 | 25073.8 |
|  |  |  |  |  |  |  | 92 | 0 |
|  |  |  |  |  |  |  | 93 | 0 |
|  |  |  |  |  |  |  | 94 | 0 |
|  |  |  |  |  |  |  | 95 | 0 |
|  |  |  |  |  |  |  | 96 | 0 |
|  |  |  |  |  |  |  | 97 | 0 |
|  |  |  |  |  |  |  | 98 | 0 |
|  |  |  |  |  |  |  | 99 | 129915 |
|  |  |  |  |  |  |  | 100 | 129942 |
|  |  |  |  |  |  |  | 101 | 0 |
|  |  |  |  |  |  |  | 102 | 0 |
|  |  |  |  |  |  |  | 103 | 0 |
|  |  |  |  |  |  |  | 104 | 0 |
|  |  |  |  |  |  |  | 105 | 0 |
|  |  |  |  |  |  |  | 106 | 0 |
|  |  |  |  |  |  |  | 107 | 0 |
|  |  |  |  |  |  |  | 108 | 0 |
|  |  |  |  |  |  |  | 109 | 437873 |
|  |  |  |  |  |  |  | 110 | 437874 |
|  |  |  |  |  |  |  | 111 | 0 |
|  |  |  |  |  |  |  | 112 | 0 |
|  |  |  |  |  |  |  | 113 | 0 |
|  |  |  |  |  |  |  | 114 | 0 |
|  |  |  |  |  |  |  | 115 | 0 |
|  |  |  |  |  |  |  | 116 | 3949.84 |
|  |  |  |  |  |  |  | 117 | 3951.55 |
|  |  |  |  |  |  |  | 118 | 0 |
|  |  |  |  |  |  |  | 119 | 1150870 |
|  |  |  |  |  |  |  | 120 | 1150840 |
|  |  |  |  |  |  |  | 121 | 0 |
|  |  |  |  |  |  |  | 122 | 0 |
|  |  |  |  |  |  |  | 123 | 76391.4 |
|  |  |  |  |  |  |  | 124 | 104475 |
|  |  |  |  |  |  |  | 125 | 28081.1 |
|  |  |  |  |  |  |  | 126 | 0 |
|  |  |  |  |  |  |  | 127 | 0 |
|  |  |  |  |  |  |  | 128 | 0 |
|  |  |  |  |  |  |  | 129 | 44477.3 |
|  |  |  |  |  |  |  | 130 | 44471.2 |
|  |  |  |  |  |  |  | 131 | 731981 |
|  |  |  |  |  |  |  | 132 | 731981 |
|  |  |  |  |  |  |  | 133 | 0 |
|  |  |  |  |  |  |  | 134 | 30013.3 |
|  |  |  |  |  |  |  | 135 | 30015.5 |
|  |  |  |  |  |  |  | 136 | 0 |
|  |  |  |  |  |  |  | 137 | 0 |
|  |  |  |  |  |  |  | 138 | 0 |
|  |  |  |  |  |  |  | 139 | 0 |
|  |  |  |  |  |  |  | 140 | 0 |
|  |  |  |  |  |  |  | 141 | 0 |
|  |  |  |  |  |  |  | 142 | 1634.43 |
|  |  |  |  |  |  |  | 143 | 1634.62 |
|  |  |  |  |  |  |  | 144 | 0 |
|  |  |  |  |  |  |  | 145 | 0 |
|  |  |  |  |  |  |  | 146 | 3331.03 |
|  |  |  |  |  |  |  | 147 | 3330.01 |
|  |  |  |  |  |  |  | 148 | 0 |
|  |  |  |  |  |  |  | 149 | 19458.7 |
|  |  |  |  |  |  |  | 150 | 19453.9 |
|  |  |  |  |  |  |  | 151 | 0 |
|  |  |  |  |  |  |  | 152 | 0 |
|  |  |  |  |  |  |  | 153 | 0 |
|  |  |  |  |  |  |  | 154 | 137442 |
|  |  |  |  |  |  |  | 155 | 137422 |
|  |  |  |  |  |  |  | 156 | 0 |
|  |  |  |  |  |  |  | 157 | 0 |
|  |  |  |  |  |  |  | 158 | 0 |
|  |  |  |  |  |  |  | 159 | 423597 |

**Supplementary Table S1. continued.**

| **Age (Ma) (*17*)** | **Sea Level (m) (*17*)** | **Age (Ma) (*17*)** | **Sea Level (m) (*17*)** | **Age (Ma) (*17*)** | **Sea Level (m) (*17*)** | **Age (Ma) (*17*)** | **Sea Level (m) (*17*)** |
| --- | --- | --- | --- | --- | --- | --- | --- |
| 0 | -1.6 | ** | ** | 2.28 | -44.0 | 3.43 | -29.6 |
| 0.005 | -3.3 | ** | ** | 2.285 | -41.6 | 3.435 | -35.4 |
| 0.01 | -52.4 | 1.15 | -38.9 | 2.29 | -27.5 | 3.44 | -28.4 |
| 0.015 | -120.4 | 2.625 | -6.9 | 2.295 | -8.8 | 3.445 | -18.2 |
| 0.02 | -122.2 | 1.155 | -41.1 | 2.3 | 10.4 | 3.45 | -8.4 |
| 0.025 | -99.1 | 1.16 | -20.6 | 2.305 | 1.6 | 3.455 | 3.0 |
| 0.03 | -102.6 | 1.165 | -26.3 | 2.31 | -7.5 | 3.46 | -22.4 |
| 0.035 | -91.0 | 1.17 | -18.3 | 2.315 | -25.4 | 3.465 | -10.6 |
| 0.04 | -85.7 | 1.175 | -31.8 | 2.32 | -16.0 | 3.47 | 4.4 |
| 0.045 | -80.4 | 1.18 | -19.0 | 2.325 | 4.0 | 3.475 | -6.5 |
| 0.05 | -75.3 | 1.185 | -33.4 | 2.33 | 0.0 | 3.48 | -16.8 |
| 0.055 | -87.8 | 1.19 | -60.4 | 2.335 | -1.1 | 3.485 | -13.0 |
| 0.06 | -76.1 | 1.195 | -56.4 | 2.34 | -9.9 | 3.49 | -4.9 |
| 0.065 | -85.2 | 1.2 | -42.1 | 2.345 | 11.2 | 3.495 | -1.7 |
| 0.07 | -57.9 | 1.205 | -59.5 | 2.35 | 23.2 | 3.5 | 5.0 |
| 0.075 | -52.9 | 1.21 | -57.5 | 2.355 | 1.6 | 3.505 | -2.2 |
| 0.08 | -47.4 | 1.215 | -60.0 | 2.36 | -28.3 | 3.51 | 2.4 |
| 0.085 | -49.0 | 1.22 | -47.2 | 2.365 | -40.2 | 3.515 | -1.1 |
| 0.09 | -55.1 | 1.225 | -29.6 | 2.37 | -29.3 | 3.52 | -12.8 |
| 0.095 | -50.6 | 1.23 | -25.1 | 2.375 | -17.3 | 3.525 | -1.6 |
| 0.1 | -39.2 | 1.235 | -23.6 | 2.38 | -8.8 | 3.53 | -4.8 |
| 0.105 | -42.8 | 1.24 | -7.8 | 2.385 | 6.0 | 3.535 | 8.8 |
| 0.11 | -72.4 | 1.245 | -36.6 | 2.39 | 24.8 | 3.54 | 1.6 |
| 0.115 | -4.3 | 1.25 | -78.6 | 2.395 | 20.8 | 3.545 | -10.8 |
| 0.12 | 24.0 | 1.255 | -91.7 | 2.4 | -4.8 | 3.55 | -17.6 |
| 0.125 | -1.9 | 1.26 | -71.6 | 2.405 | -24.0 | 3.555 | 3.6 |
| 0.13 | -76.3 | 1.265 | -61.9 | 2.41 | -19.7 | 3.56 | -18.4 |
| 0.135 | -96.9 | 1.27 | -42.4 | 2.415 | -20.0 | 3.565 | -8.0 |
| 0.14 | -107.7 | 1.275 | -30.8 | 2.42 | -8.8 | 3.57 | -10.4 |
| 0.145 | -97.0 | 1.28 | -34.1 | 2.425 | 0.0 | 3.575 | 4.4 |
| 0.15 | -93.9 | 1.285 | -61.9 | 2.43 | -7.0 | 3.58 | -7.2 |
| 0.155 | -102.2 | 1.29 | -70.3 | 2.435 | -1.6 | 3.585 | -21.6 |
| 0.16 | -90.3 | 1.295 | -55.9 | 2.44 | -12.0 | 3.59 | -28.0 |
| 0.165 | -78.5 | 1.3 | -55.6 | 2.445 | -54.4 | 3.595 | -7.5 |
| 0.17 | -67.8 | 1.305 | -55.0 | 2.45 | -49.6 | 3.6 | -2.4 |
| 0.175 | -72.0 | 1.31 | -40.5 | 2.455 | -35.2 | 3.605 | -9.8 |
| 0.18 | -81.7 | 1.315 | -29.7 | 2.46 | -36.0 | 3.61 | -8.5 |
| 0.185 | -45.3 | 1.32 | -25.5 | 2.465 | -21.6 | 3.615 | 18.4 |
| 0.19 | -49.0 | 1.325 | -58.7 | 2.47 | 8.8 | 3.62 | -0.8 |
| 0.195 | -18.2 | 1.33 | -56.5 | 2.475 | 10.8 | 3.625 | -23.8 |
| 0.2 | -34.2 | 1.335 | -58.2 | 2.48 | -21.6 | 3.63 | -25.1 |
| 0.205 | -17.6 | 1.34 | -50.9 | 2.485 | -57.2 | 3.635 | -34.4 |
| 0.21 | -13.4 | 1.345 | -40.7 | 2.49 | -50.4 | 3.64 | 3.2 |
| 0.215 | -7.3 | 1.35 | -11.8 | 2.495 | -39.5 | 3.645 | 1.2 |
| 0.22 | -23.6 | 1.355 | -4.7 | 2.5 | -30.4 | 3.65 | 1.6 |
| 0.225 | -74.1 | 1.36 | -36.9 | 2.505 | -4.6 | 3.655 | 7.2 |
| 0.23 | -68.3 | 1.365 | -49.8 | 2.51 | -9.2 | 3.66 | -2.1 |
| 0.235 | -59.5 | 1.37 | -64.9 | 2.515 | -28.9 | 3.665 | -12.6 |
| 0.24 | -28.6 | 1.375 | -58.0 | 2.52 | -46.4 | 3.67 | -20.8 |
| 0.245 | -60.3 | 1.38 | -55.4 | 2.525 | -56.2 | 3.675 | -26.7 |
| 0.25 | -66.8 | 1.385 | -38.5 | 2.53 | -53.2 | 3.68 | -30.0 |
| 0.255 | -85.4 | 1.39 | -25.7 | 2.535 | -36.6 | 3.685 | -22.7 |
| 0.26 | -79.1 | 1.395 | -11.6 | 2.54 | -2.4 | 3.69 | -17.6 |
| 0.265 | -79.7 | 1.4 | -19.7 | 2.545 | 17.8 | 3.695 | -0.8 |
| 0.27 | -86.0 | 1.405 | -36.2 | 2.55 | -21.2 | 3.7 | 20.8 |
| 0.275 | -54.1 | 1.41 | -49.7 | 2.555 | -15.8 | 3.705 | -9.6 |
| 0.28 | -43.4 | 1.415 | -63.9 | 2.56 | -6.0 | 3.71 | 1.9 |
| 0.285 | -39.3 | 1.42 | -69.3 | 2.565 | -7.1 | 3.715 | -11.2 |
| 0.29 | -39.5 | 1.425 | -61.9 | 2.57 | -2.8 | 3.72 | -20.0 |
| 0.295 | -55.8 | 1.43 | -39.1 | 2.575 | 3.4 | 3.725 | -10.4 |
| 0.3 | -45.8 | 1.435 | -7.2 | 2.58 | 11.2 | 3.73 | -4.4 |
| 0.305 | -39.7 | 1.44 | -3.5 | 2.585 | 10.7 | 3.735 | 6.8 |
| 0.31 | -34.8 | 1.445 | 4.3 | 2.59 | 6.3 | 3.74 | 6.0 |
| 0.315 | -23.0 | 1.45 | 10.1 | 2.595 | -3.4 | 3.745 | 4.0 |
| 0.32 | -31.4 | 1.455 | -44.2 | 2.6 | -35.2 | 3.75 | 3.4 |
| 0.325 | 11.1 | 1.46 | -75.0 | 2.605 | -55.4 | 3.755 | -0.3 |
| 0.33 | -16.8 | 1.465 | -50.1 | 2.61 | -42.8 | 3.76 | -9.3 |
| 0.335 | -26.0 | 1.47 | -27.0 | 2.615 | -41.0 | 3.765 | 1.6 |
| 0.34 | -83.1 | 1.475 | -22.1 | 2.62 | -28.8 | 3.77 | 16.8 |
| 0.345 | -96.8 | 1.48 | -6.5 | 2.63 | -4.8 | 3.775 | 12.8 |
| 0.35 | -92.0 | 1.485 | 2.7 | 2.635 | 4.2 | 3.78 | 15.4 |
| 0.355 | -90.6 | 1.49 | 0.0 | 2.64 | -1.6 | 3.785 | -7.2 |
| 0.36 | -104.0 | 1.495 | -26.3 | 2.645 | -29.5 | 3.79 | -20.8 |
| 0.365 | -55.4 | 1.5 | -60.7 | 2.65 | -26.4 | 3.795 | -19.0 |
| 0.37 | -85.2 | 1.505 | -68.1 | 2.655 | -36.2 | 3.8 | 1.0 |
| 0.375 | -58.4 | 1.51 | -58.3 | 2.66 | -16.0 | 3.805 | -2.9 |
| 0.38 | -52.7 | 1.515 | -41.3 | 2.665 | 7.0 | 3.81 | -8.0 |
| 0.385 | -52.1 | 1.52 | -19.4 | 2.67 | 11.6 | 3.815 | -11.7 |
| 0.39 | -31.9 | 1.525 | -22.3 | 2.675 | 6.9 | 3.82 | -9.6 |
| 0.395 | -30.6 | 1.53 | -41.4 | 2.68 | -1.2 | 3.825 | -14.0 |
| 0.4 | -9.5 | 1.535 | -52.8 | 2.685 | -14.6 | 3.83 | -3.0 |
| 0.405 | 12.5 | 1.54 | -78.4 | 2.69 | -42.0 | 3.835 | -0.8 |
| 0.41 | 12.8 | 1.545 | -66.2 | 2.695 | -39.8 | 3.84 | -17.4 |
| 0.415 | -0.2 | 1.55 | -34.2 | 2.7 | -18.8 | 3.845 | -28.8 |
| 0.42 | -29.5 | 1.555 | -44.2 | 2.705 | -19.0 | 3.85 | 7.2 |
| 0.425 | -84.7 | 1.56 | -31.6 | 2.71 | -40.4 | 3.855 | 18.6 |
| 0.43 | -115.3 | 1.565 | -32.4 | 2.715 | -38.2 | 3.86 | 22.1 |
| 0.435 | -122.1 | 1.57 | -27.5 | 2.72 | -45.2 | 3.865 | -0.8 |
| 0.44 | -122.7 | 1.575 | -60.8 | 2.725 | -29.0 | 3.87 | 14.0 |
| 0.445 | -102.4 | 1.58 | -54.1 | 2.73 | -54.8 | 3.875 | -2.4 |
| 0.45 | -84.8 | 1.585 | -38.1 | 2.735 | -30.2 | 3.88 | -11.8 |
| 0.455 | -87.5 | 1.59 | -33.7 | 2.74 | -25.6 | 3.885 | -18.8 |
| 0.46 | -82.7 | 1.595 | -29.9 | 2.745 | -29.8 | 3.89 | -0.6 |
| 0.465 | -99.9 | 1.6 | -31.5 | 2.75 | 20.4 | 3.895 | -1.1 |
| 0.47 | -96.7 | 1.605 | -25.3 | 2.755 | 21.8 | 3.9 | 14.4 |
| 0.475 | -73.7 | 1.61 | -24.0 | 2.76 | 14.4 | 3.905 | -12.0 |
| 0.48 | -53.4 | 1.615 | -28.6 | 2.765 | 4.6 | 3.91 | -2.4 |
| 0.485 | -35.0 | 1.62 | -33.2 | 2.77 | -5.2 | 3.915 | -5.6 |
| 0.49 | -34.9 | 1.625 | -28.1 | 2.775 | -18.2 | 3.92 | 4.0 |
| 0.495 | -39.7 | 1.63 | -38.4 | 2.78 | -4.8 | 3.925 | -5.6 |
| 0.5 | -23.4 | 1.635 | -32.2 | 2.785 | 5.4 | 3.93 | -3.6 |
| 0.505 | -60.8 | 1.64 | -39.3 | 2.79 | 6.5 | 3.935 | 3.2 |
| 0.51 | -68.6 | 1.645 | -65.1 | 2.795 | -8.6 | 3.94 | 13.3 |
| 0.515 | -78.8 | 1.65 | -49.3 | 2.8 | -2.0 | 3.945 | -14.7 |
| 0.52 | -61.2 | 1.655 | -69.8 | 2.805 | -8.0 | 3.95 | -14.1 |
| 0.525 | -54.8 | 1.66 | -59.2 | 2.81 | -32.4 | 3.955 | 5.6 |
| 0.53 | -78.3 | 1.665 | -60.4 | 2.815 | -39.8 | 3.96 | -10.8 |
| 0.535 | -75.3 | 1.67 | -29.9 | 2.82 | -43.2 | 3.965 | -10.4 |
| 0.54 | -84.6 | 1.675 | -21.8 | 2.825 | -17.0 | 3.97 | -16.8 |
| 0.545 | -73.7 | 1.68 | -20.4 | 2.83 | -12.4 | 3.975 | -18.4 |
| 0.55 | -66.5 | 1.685 | -15.0 | 2.835 | -4.0 | 3.98 | -13.6 |
| 0.555 | -73.0 | 1.69 | -34.5 | 2.84 | -5.6 | 3.985 | -24.5 |
| 0.56 | -65.0 | 1.695 | -56.7 | 2.845 | -22.6 | 3.99 | -23.2 |
| 0.565 | -50.6 | 1.7 | -49.2 | 2.85 | -17.2 | 3.995 | -56.4 |
| 0.57 | -41.6 | 1.705 | -48.0 | 2.855 | -29.4 | 4 | -40.8 |
| 0.575 | -25.6 | 1.71 | -36.6 | 2.86 | -26.4 | 4.005 | -25.6 |
| 0.58 | -25.6 | 1.715 | -25.1 | 2.865 | -7.8 | 4.01 | -17.2 |
| 0.585 | -67.6 | 1.72 | -20.3 | 2.87 | -5.5 | 4.015 | 3.2 |
| 0.59 | -79.1 | 1.725 | -32.7 | 2.875 | -15.0 | 4.02 | -16.0 |
| 0.595 | -56.5 | 1.73 | -10.0 | 2.88 | -6.4 | 4.025 | -10.4 |
| 0.6 | -49.0 | 1.735 | -30.7 | 2.885 | -33.0 | 4.03 | -12.0 |
| 0.605 | -52.8 | 1.74 | -17.5 | 2.89 | -40.0 | 4.035 | -33.3 |
| 0.61 | -39.2 | 1.745 | -15.2 | 2.895 | -22.6 | 4.04 | -36.6 |
| 0.615 | -50.7 | 1.75 | -54.5 | 2.9 | -13.6 | 4.045 | -34.8 |
| 0.62 | -72.9 | 1.755 | -25.8 | 2.905 | -5.8 | 4.05 | -34.4 |
| 0.625 | -117.8 | 1.76 | -10.9 | 2.91 | -5.6 | 4.055 | -18.4 |
| 0.63 | -124.2 | 1.765 | -28.4 | 2.915 | -14.5 | 4.06 | -12.4 |
| 0.635 | -114.3 | 1.77 | -2.9 | 2.92 | -8.8 | 4.065 | -11.2 |
| 0.64 | -122.7 | 1.775 | -14.2 | 2.925 | -9.0 | 4.07 | -5.9 |
| 0.645 | -111.9 | 1.78 | -43.3 | 2.93 | -26.0 | 4.075 | -12.2 |
| 0.65 | -106.4 | 1.785 | -28.8 | 2.935 | -40.6 | 4.08 | -3.7 |
| 0.655 | -101.6 | 1.79 | -52.0 | 2.94 | -5.6 | 4.085 | -8.6 |
| 0.66 | -93.3 | 1.795 | -43.5 | 2.945 | -0.2 | 4.09 | -19.8 |
| 0.665 | -80.5 | 1.8 | -18.9 | 2.95 | 15.6 | 4.095 | -20.8 |
| 0.67 | -91.7 | 1.805 | -40.0 | 2.955 | 14.6 | 4.1 | -5.2 |
| 0.675 | -80.0 | 1.81 | -37.6 | 2.96 | -1.6 | 4.105 | 10.4 |
| 0.68 | -82.8 | 1.815 | -9.6 | 2.965 | 3.0 | 4.11 | 4.0 |
| 0.685 | -82.4 | 1.82 | -21.6 | 2.97 | -13.7 | 4.115 | -2.7 |
| 0.69 | -53.8 | 1.825 | -48.7 | 2.975 | -22.2 | 4.12 | -10.4 |
| 0.695 | -62.2 | 1.83 | -23.4 | 2.98 | -6.4 | 4.125 | -1.2 |
| 0.7 | -70.5 | 1.835 | -19.7 | 2.985 | 12.3 | 4.13 | -12.0 |
| 0.705 | -78.9 | 1.84 | -46.8 | 2.99 | 13.2 | 4.135 | -3.2 |
| 0.71 | -87.2 | 1.845 | -14.4 | 2.995 | 17.0 | 4.14 | -16.8 |
| 0.715 | -95.1 | 1.85 | -11.2 | 3 | 2.4 | 4.145 | -20.5 |
| 0.72 | -96.4 | 1.855 | 14.4 | 3.005 | -13.3 | 4.15 | -7.6 |
| 0.725 | -63.7 | 1.86 | -10.4 | 3.01 | -20.1 | 4.155 | 12.0 |
| 0.73 | -70.4 | 1.865 | -22.0 | 3.015 | -32.2 | 4.16 | 10.2 |
| 0.735 | -75.5 | 1.87 | -16.0 | 3.02 | -17.6 | 4.165 | 6.2 |
| 0.74 | -77.6 | 1.875 | -17.6 | 3.025 | -17.8 | 4.17 | 0.6 |
| 0.745 | -73.6 | 1.88 | -17.6 | 3.03 | -15.6 | 4.175 | 9.6 |
| 0.75 | -86.1 | 1.885 | -7.6 | 3.035 | 3.8 | 4.18 | -0.4 |
| 0.755 | -91.5 | 1.89 | 3.2 | 3.04 | 9.6 | 4.185 | 3.2 |
| 0.76 | -85.4 | 1.895 | -5.6 | 3.045 | 8.1 | 4.19 | 0.8 |
| 0.765 | -62.3 | 1.9 | -11.2 | 3.05 | -2.5 | 4.195 | -2.1 |
| 0.77 | -53.0 | 1.905 | -36.8 | 3.055 | -3.8 | 4.2 | -2.6 |
| 0.775 | -45.1 | 1.91 | -56.0 | 3.06 | -11.2 | 4.205 | 6.9 |
| 0.78 | -21.8 | 1.915 | -68.4 | 3.065 | 7.0 | 4.21 | -13.6 |
| 0.785 | -44.2 | 1.92 | -40.0 | 3.07 | 14.0 | 4.215 | -18.4 |
| 0.79 | -48.5 | 1.925 | -40.0 | 3.075 | 20.2 | 4.22 | 5.2 |
| 0.795 | -98.0 | 1.93 | -16.8 | 3.08 | 7.2 | 4.225 | 3.2 |
| 0.8 | -82.5 | 1.935 | -12.0 | 3.085 | -10.2 | 4.23 | -7.6 |
| 0.805 | -77.2 | 1.94 | -14.1 | 3.09 | -11.3 | 4.235 | -20.0 |
| 0.81 | -58.6 | 1.945 | -11.6 | 3.095 | -6.2 | 4.24 | -12.6 |
| 0.815 | -41.3 | 1.95 | -12.8 | 3.1 | -6.4 | 4.245 | 3.2 |
| 0.82 | -55.7 | 1.955 | -44.0 | 3.105 | 2.7 | 4.25 | -12.8 |
| 0.825 | -52.6 | 1.96 | -37.2 | 3.11 | 2.0 | 4.255 | -34.0 |
| 0.83 | -51.1 | 1.965 | -31.6 | 3.115 | -7.8 | 4.26 | -16.4 |
| 0.835 | -48.4 | 1.97 | -14.4 | 3.12 | -18.9 | 4.265 | -16.0 |
| 0.84 | -27.6 | 1.975 | -3.2 | 3.125 | -41.0 | 4.27 | 4.0 |
| 0.845 | -24.3 | 1.98 | 5.6 | 3.13 | -10.5 | 4.275 | 10.6 |
| 0.85 | -36.2 | 1.985 | -42.4 | 3.135 | -33.8 | 4.28 | 9.5 |
| 0.855 | -33.0 | 1.99 | -28.0 | 3.14 | -48.8 | 4.285 | -8.8 |
| 0.86 | -19.3 | 1.995 | -29.3 | 3.145 | -50.6 | 4.29 | -16.3 |
| 0.865 | -61.6 | 2 | -28.3 | 3.15 | -24.9 | 4.295 | -7.2 |
| 0.87 | -83.6 | 2.005 | -34.4 | 3.155 | -13.9 | 4.3 | 0.4 |
| 0.875 | -109.3 | 2.01 | -29.6 | 3.16 | -4.4 | 4.305 | -10.7 |
| 0.88 | -106.9 | 2.015 | -16.0 | 3.165 | -10.6 | 4.31 | -17.0 |
| 0.885 | -93.7 | 2.02 | 6.1 | 3.17 | -10.5 | 4.315 | 3.2 |
| 0.89 | -90.1 | 2.025 | -3.6 | 3.175 | -23.0 | 4.32 | 14.4 |
| 0.895 | -84.3 | 2.03 | -17.6 | 3.18 | -20.0 | 4.325 | 8.0 |
| 0.9 | -64.2 | 2.035 | -12.8 | 3.185 | -29.5 | 4.33 | 16.8 |
| 0.905 | -60.3 | 2.04 | -13.2 | 3.19 | -23.1 | 4.335 | 4.0 |
| 0.91 | -74.6 | 2.045 | -36.0 | 3.195 | -2.2 | 4.34 | 0.5 |
| 0.915 | -89.1 | 2.05 | -11.2 | 3.2 | -0.2 | 4.345 | 4.0 |
| 0.92 | -66.8 | 2.055 | 17.6 | 3.205 | -4.2 | 4.35 | -14.9 |
| 0.925 | -51.5 | 2.06 | -4.0 | 3.21 | -12.6 | 4.355 | -2.4 |
| 0.93 | -31.2 | 2.065 | -6.4 | 3.215 | -6.0 | 4.36 | -10.9 |
| 0.935 | -22.2 | 2.07 | -13.6 | 3.22 | -13.6 | 4.365 | -7.7 |
| 0.94 | -19.8 | 2.075 | -36.0 | 3.225 | -23.8 | 4.37 | 2.7 |
| 0.945 | -9.8 | 2.08 | -36.8 | 3.23 | -4.4 | 4.375 | -22.4 |
| 0.95 | 2.0 | 2.085 | -69.6 | 3.235 | -15.6 | 4.38 | -8.0 |
| 0.955 | -40.6 | 2.09 | -37.6 | 3.24 | -7.2 | 4.385 | -8.8 |
| 0.96 | -41.1 | 2.095 | -41.2 | 3.245 | -16.2 | 4.39 | -6.4 |
| 0.965 | -57.3 | 2.1 | -6.0 | 3.25 | -7.6 | 4.395 | -2.7 |
| 0.97 | -38.6 | 2.105 | -13.6 | 3.255 | -15.0 | 4.4 | -17.3 |
| 0.975 | -27.9 | 2.11 | -24.0 | 3.26 | -4.6 | 4.405 | -0.4 |
| 0.98 | -42.6 | 2.115 | -18.7 | 3.265 | -11.4 | 4.41 | -3.2 |
| 0.985 | -46.6 | 2.12 | -14.4 | 3.27 | -13.2 | 4.415 | 16.0 |
| 0.99 | -29.3 | 2.125 | -32.8 | 3.275 | -0.6 | 4.42 | -21.1 |
| 0.995 | -48.1 | 2.13 | -24.6 | 3.28 | -10.4 | 4.425 | -2.0 |
| 1 | -54.7 | 2.135 | -10.9 | 3.285 | 0.9 | 4.43 | 11.2 |
| 1.005 | -65.7 | 2.14 | 4.8 | 3.29 | -7.6 | 4.435 | -1.4 |
| 1.01 | -66.8 | 2.145 | -8.8 | 3.295 | -3.5 | 4.44 | 13.9 |
| 1.015 | -31.9 | 2.15 | -44.3 | 3.3 | -12.0 | 4.445 | -3.2 |
| 1.02 | -27.0 | 2.155 | -46.4 | 3.305 | -67.4 | 4.45 | -8.0 |
| 1.025 | -38.0 | 2.16 | -64.8 | 3.31 | -51.3 | 4.455 | 18.8 |
| 1.03 | -68.6 | 2.165 | -60.8 | 3.315 | -62.2 | 4.46 | 3.0 |
| 1.035 | -75.2 | 2.17 | -49.6 | 3.32 | -48.0 | 4.465 | 7.5 |
| 1.04 | -85.3 | 2.175 | -38.4 | 3.325 | -34.3 | 4.47 | 15.4 |
| 1.045 | -75.1 | 2.18 | -31.7 | 3.33 | -32.0 | 4.475 | 14.2 |
| 1.05 | -69.1 | 2.185 | -20.5 | 3.335 | -39.8 | 4.48 | 5.3 |
| 1.055 | -63.4 | 2.19 | -5.6 | 3.34 | -62.0 | 4.485 | 20.8 |
| 1.06 | -56.6 | 2.195 | -6.8 | 3.345 | -45.8 | 4.49 | 1.6 |
| 1.065 | -5.8 | 2.2 | -26.8 | 3.35 | -51.6 | 4.495 | 0.4 |
| 1.07 | -48.6 | 2.205 | -28.8 | 3.355 | -42.2 | 4.5 | -2.4 |
| 1.075 | -15.1 | 2.21 | -2.0 | 3.36 | -32.8 | 4.505 | -3.6 |
| 1.08 | -20.4 | 2.215 | 0.4 | 3.365 | -31.0 | 4.51 | -3.2 |
| 1.085 | -55.5 | 2.22 | -2.4 | 3.37 | -15.6 | 4.515 | -17.6 |
| 1.09 | -68.3 | 2.225 | -6.4 | 3.375 | -16.6 | 4.52 | -19.2 |
| 1.095 | -58.7 | 2.23 | -8.8 | 3.38 | -15.7 | 4.525 | 1.3 |
| 1.1 | -73.9 | 2.235 | 2.4 | 3.385 | -19.4 | 4.53 | 9.6 |
| 1.105 | -40.0 | 2.24 | -28.0 | 3.39 | -17.2 | 4.535 | -2.4 |
| 1.11 | -37.7 | 2.245 | -47.6 | 3.395 | -27.5 | 4.54 | -3.2 |
| 1.115 | -52.8 | 2.25 | -24.0 | 3.4 | -24.0 | 4.545 | -6.4 |
| 1.12 | -70.5 | 2.255 | -6.4 | 3.405 | -21.0 | 4.55 | -8.4 |
| 1.125 | -79.4 | 2.26 | -1.6 | 3.41 | 2.8 | 4.555 | -24.0 |
| 1.13 | -77.1 | 2.265 | 13.6 | 3.415 | -30.2 | 4.56 | -21.2 |
| 1.135 | -75.1 | 2.27 | 3.2 | 3.42 | -21.2 | 4.565 | -15.2 |
| 1.14 | -72.0 | 2.275 | -34.4 | 3.425 | -15.8 | 4.57 | -13.6 |

| **Age (Ma) (*17*)** | **Sea Level (m) (*17*)** | **Age (Ma) (*17*)** | **Sea Level (m) (*17*)** | **Age (Ma) (*17*)** | **Sea Level (m) (*17*)** | **Age (Ma) (*17*)** | **Sea Level (m) (*17*)** |
| --- | --- | --- | --- | --- | --- | --- | --- |
| 4.575 | -12.0 | 5.72 | -32.0 | 6.865 | -7.8 | 8.01 | 11.5 |
| 4.58 | -10.1 | 5.725 | -2.4 | 6.87 | 2.0 | 8.015 | 5.6 |
| 4.585 | 2.4 | 5.73 | -32.0 | 6.875 | 19.3 | 8.02 | -9.6 |
| 4.59 | -10.8 | 5.735 | -6.4 | 6.88 | -3.7 | 8.025 | -4.3 |
| 4.595 | -31.2 | 5.74 | -7.2 | 6.885 | -0.8 | 8.03 | -1.8 |
| 4.6 | -12.0 | 5.745 | -12.0 | 6.89 | -7.0 | 8.035 | -4.7 |
| 4.605 | 0.8 | 5.75 | -4.0 | 6.895 | -6.6 | 8.04 | -2.0 |
| 4.61 | -16.8 | 5.755 | -13.6 | 6.9 | -16.1 | 8.045 | 3.3 |
| 4.615 | -18.0 | 5.76 | -4.8 | 6.905 | 3.9 | 8.05 | 9.5 |
| 4.62 | -4.4 | 5.765 | -18.4 | 6.91 | -4.3 | 8.055 | 10.3 |
| 4.625 | 2.4 | 5.77 | -1.6 | 6.915 | 1.9 | 8.06 | 0.7 |
| 4.63 | -18.7 | 5.775 | -6.4 | 6.92 | 7.6 | 8.065 | -4.3 |
| 4.635 | -21.1 | 5.78 | 1.2 | 6.925 | 10.1 | 8.07 | -7.1 |
| 4.64 | -7.2 | 5.785 | -6.4 | 6.93 | 5.4 | 8.075 | -6.0 |
| 4.645 | -7.2 | 5.79 | -17.2 | 6.935 | -2.8 | 8.08 | -5.0 |
| 4.65 | 3.5 | 5.795 | 8.0 | 6.94 | -4.8 | 8.085 | -7.9 |
| 4.655 | -1.1 | 5.8 | 3.2 | 6.945 | -4.1 | 8.09 | -6.5 |
| 4.66 | 8.8 | 5.805 | -7.2 | 6.95 | -7.9 | 8.095 | -3.4 |
| 4.665 | -5.1 | 5.81 | -12.8 | 6.955 | 5.2 | 8.1 | -13.5 |
| 4.67 | -37.6 | 5.815 | -18.4 | 6.96 | 14.0 | 8.105 | -17.8 |
| 4.675 | -8.8 | 5.82 | -22.4 | 6.965 | 16.7 | 8.11 | -11.6 |
| 4.68 | -27.2 | 5.825 | -14.4 | 6.97 | 12.5 | 8.115 | -9.9 |
| 4.685 | -7.0 | 5.83 | -4.4 | 6.975 | 0.8 | 8.12 | -10.2 |
| 4.69 | -18.8 | 5.835 | -14.1 | 6.98 | 5.6 | 8.125 | -0.3 |
| 4.695 | -18.4 | 5.84 | -21.6 | 6.985 | -0.1 | 8.13 | 0.6 |
| 4.7 | -5.6 | 5.845 | -16.8 | 6.99 | 8.9 | 8.135 | -7.9 |
| 4.705 | -20.8 | 5.85 | -16.4 | 6.995 | 8.3 | 8.14 | -22.5 |
| 4.71 | -28.0 | 5.855 | -22.9 | 7 | 16.7 | 8.145 | -18.7 |
| 4.715 | -27.4 | 5.86 | -10.4 | 7.005 | 3.0 | 8.15 | -12.4 |
| 4.72 | -33.3 | 5.865 | -21.9 | 7.01 | -5.6 | 8.155 | -19.9 |
| 4.725 | -7.2 | 5.87 | -9.6 | 7.015 | -3.8 | 8.16 | -4.8 |
| 4.73 | -5.3 | 5.875 | -6.7 | 7.02 | 2.3 | 8.165 | -13.2 |
| 4.735 | -29.6 | 5.88 | -6.4 | 7.025 | -2.6 | 8.17 | -4.2 |
| 4.74 | 4.8 | 5.885 | -23.2 | 7.03 | -3.9 | 8.175 | -3.1 |
| 4.745 | -10.4 | 5.89 | -22.4 | 7.035 | -11.2 | 8.18 | -1.5 |
| 4.75 | -30.4 | 5.895 | -14.4 | 7.04 | -1.3 | 8.185 | 2.1 |
| 4.755 | -31.3 | 5.9 | -16.0 | 7.045 | 7.6 | 8.19 | 8.5 |
| 4.76 | -16.3 | 5.905 | -15.5 | 7.05 | -15.4 | 8.195 | 17.7 |
| 4.765 | -7.6 | 5.91 | -20.3 | 7.055 | -1.9 | 8.2 | -0.3 |
| 4.77 | 5.6 | 5.915 | -12.0 | 7.06 | -0.7 | 8.205 | 11.2 |
| 4.775 | -9.0 | 5.92 | -20.0 | 7.065 | -0.4 | 8.21 | 5.6 |
| 4.78 | -1.0 | 5.925 | -21.6 | 7.07 | 6.8 | 8.215 | 10.1 |
| 4.785 | -4.8 | 5.93 | -26.9 | 7.075 | 7.6 | 8.22 | -2.1 |
| 4.79 | -23.0 | 5.935 | -18.7 | 7.08 | 8.0 | 8.225 | 8.0 |
| 4.795 | -9.6 | 5.94 | -26.1 | 7.085 | 13.4 | 8.23 | 1.6 |
| 4.8 | -7.2 | 5.945 | -2.9 | 7.09 | 6.9 | 8.235 | 24.8 |
| 4.805 | 21.3 | 5.95 | -2.4 | 7.095 | -4.7 | 8.24 | -2.7 |
| 4.81 | 12.0 | 5.955 | -23.2 | 7.1 | -8.9 | 8.245 | 16.8 |
| 4.815 | 12.3 | 5.96 | -6.4 | 7.105 | -7.4 | 8.25 | 8.1 |
| 4.82 | 12.6 | 5.965 | -11.5 | 7.11 | 1.6 | 8.255 | 14.1 |
| 4.825 | -30.4 | 5.97 | -16.8 | 7.115 | 8.2 | 8.26 | 19.2 |
| 4.83 | -45.6 | 5.975 | -5.6 | 7.12 | 4.5 | 8.265 | 15.5 |
| 4.835 | -38.4 | 5.98 | -16.8 | 7.125 | 0.8 | 8.27 | 16.0 |
| 4.84 | -7.6 | 5.985 | 12.8 | 7.13 | -2.9 | 8.275 | 9.2 |
| 4.845 | -8.0 | 5.99 | -9.6 | 7.135 | -6.6 | 8.28 | -14.4 |
| 4.85 | -4.8 | 5.995 | -21.6 | 7.14 | -6.3 | 8.285 | 4.7 |
| 4.855 | -1.6 | 6 | 18.4 | 7.145 | -6.3 | 8.29 | -8.8 |
| 4.86 | -11.2 | 6.005 | -15.6 | 7.15 | 3.4 | 8.295 | 13.6 |
| 4.865 | -23.6 | 6.01 | -0.8 | 7.155 | 7.5 | 8.3 | -3.2 |
| 4.87 | -42.8 | 6.015 | 8.0 | 7.16 | 6.3 | 8.305 | 12.8 |
| 4.875 | -48.4 | 6.02 | -17.6 | 7.165 | 16.2 | 8.31 | 8.8 |
| 4.88 | -40.4 | 6.025 | -5.6 | 7.17 | 9.9 | 8.315 | 22.4 |
| 4.885 | -41.6 | 6.03 | -14.4 | 7.175 | -2.4 | 8.32 | 17.5 |
| 4.89 | -11.2 | 6.035 | -16.8 | 7.18 | -7.0 | 8.325 | 27.7 |
| 4.895 | 4.0 | 6.04 | -11.2 | 7.185 | -10.4 | 8.33 | 23.2 |
| 4.9 | -4.0 | 6.045 | -30.0 | 7.19 | 1.1 | 8.335 | 8.0 |
| 4.905 | -10.1 | 6.05 | -4.0 | 7.195 | 14.0 | 8.34 | 21.7 |
| 4.91 | -12.8 | 6.055 | -3.2 | 7.2 | 17.6 | 8.345 | 4.8 |
| 4.915 | -5.6 | 6.06 | 26.4 | 7.205 | 20.0 | 8.35 | 13.1 |
| 4.92 | -3.2 | 6.065 | -0.8 | 7.21 | 14.1 | 8.355 | 16.0 |
| 4.925 | -18.4 | 6.07 | -4.2 | 7.215 | 11.3 | 8.36 | 27.2 |
| 4.93 | -21.6 | 6.075 | 16.0 | 7.22 | -0.3 | 8.365 | 21.9 |
| 4.935 | -20.0 | 6.08 | 3.2 | 7.225 | -4.7 | 8.37 | 26.7 |
| 4.94 | -10.4 | 6.085 | 1.6 | 7.23 | 0.5 | 8.375 | 26.1 |
| 4.945 | -4.4 | 6.09 | 16.0 | 7.235 | 0.5 | 8.38 | 20.0 |
| 4.95 | -14.6 | 6.095 | -7.2 | 7.24 | 4.7 | 8.385 | 15.2 |
| 4.955 | -21.6 | 6.1 | -6.0 | 7.245 | 17.6 | 8.39 | 12.5 |
| 4.96 | 3.0 | 6.105 | -3.6 | 7.25 | 9.6 | 8.395 | 10.1 |
| 4.965 | -18.4 | 6.11 | -9.6 | 7.255 | 6.2 | 8.4 | 9.3 |
| 4.97 | 1.1 | 6.115 | -9.6 | 7.26 | -5.6 | 8.405 | 7.2 |
| 4.975 | 3.7 | 6.12 | 2.0 | 7.265 | -4.0 | 8.41 | 15.7 |
| 4.98 | 9.9 | 6.125 | -24.8 | 7.27 | 0.8 | 8.415 | 11.5 |
| 4.985 | -0.8 | 6.13 | -41.9 | 7.275 | 4.8 | 8.42 | 18.7 |
| 4.99 | 4.3 | 6.135 | -34.1 | 7.28 | 10.0 | 8.425 | 23.7 |
| 4.995 | -3.4 | 6.14 | 3.5 | 7.285 | 6.6 | 8.43 | 12.8 |
| 5 | -7.2 | 6.145 | 0.1 | 7.29 | 2.3 | 8.435 | 12.3 |
| 5.005 | -6.2 | 6.15 | 1.1 | 7.295 | -0.5 | 8.44 | 13.6 |
| 5.01 | -19.2 | 6.155 | -4.3 | 7.3 | 0.0 | 8.445 | 14.7 |
| 5.015 | 0.0 | 6.16 | -5.1 | 7.305 | 1.6 | 8.45 | 18.4 |
| 5.02 | -1.0 | 6.165 | 20.6 | 7.31 | 2.0 | 8.455 | 12.8 |
| 5.025 | 5.0 | 6.17 | -0.3 | 7.315 | 10.5 | 8.46 | 10.1 |
| 5.03 | 4.3 | 6.175 | -6.8 | 7.32 | 12.8 | 8.465 | -2.8 |
| 5.035 | -2.4 | 6.18 | 0.3 | 7.325 | 16.0 | 8.47 | -0.3 |
| 5.04 | -22.1 | 6.185 | 2.6 | 7.33 | 11.8 | 8.475 | 4.0 |
| 5.045 | -28.8 | 6.19 | 3.4 | 7.335 | 4.6 | 8.48 | 24.8 |
| 5.05 | -19.5 | 6.195 | 3.6 | 7.34 | -1.6 | 8.485 | 9.3 |
| 5.055 | -11.7 | 6.2 | -21.4 | 7.345 | -6.8 | 8.49 | 14.4 |
| 5.06 | -12.6 | 6.205 | -16.0 | 7.35 | 4.6 | 8.495 | 9.1 |
| 5.065 | -8.0 | 6.21 | 3.1 | 7.355 | 6.4 | 8.5 | 5.6 |
| 5.07 | -4.3 | 6.215 | -0.2 | 7.36 | 10.8 | 8.505 | -6.4 |
| 5.075 | -10.2 | 6.22 | 4.8 | 7.365 | 16.0 | 8.51 | 10.7 |
| 5.08 | -4.5 | 6.225 | 10.4 | 7.37 | 8.8 | 8.515 | 19.6 |
| 5.085 | 12.6 | 6.23 | -5.3 | 7.375 | 8.9 | 8.52 | 9.1 |
| 5.09 | -5.6 | 6.235 | -12.3 | 7.38 | 7.8 | 8.525 | 14.4 |
| 5.095 | 16.0 | 6.24 | -22.0 | 7.385 | 2.0 | 8.53 | -12.8 |
| 5.1 | 6.0 | 6.245 | -10.3 | 7.39 | 2.8 | 8.535 | 27.4 |
| 5.105 | 25.6 | 6.25 | 0.0 | 7.395 | 4.9 | 8.54 | 13.9 |
| 5.11 | -4.0 | 6.255 | 2.7 | 7.4 | -0.8 | 8.545 | 15.8 |
| 5.115 | -17.6 | 6.26 | -1.7 | 7.405 | -1.8 | 8.55 | 20.0 |
| 5.12 | -2.4 | 6.265 | 6.6 | 7.41 | -1.4 | 8.555 | 16.0 |
| 5.125 | 4.0 | 6.27 | 3.2 | 7.415 | 2.1 | 8.56 | 12.5 |
| 5.13 | 16.0 | 6.275 | -8.6 | 7.42 | 6.5 | 8.565 | 15.2 |
| 5.135 | 26.8 | 6.28 | 1.5 | 7.425 | 10.3 | 8.57 | 18.4 |
| 5.14 | 6.4 | 6.285 | 2.9 | 7.43 | 14.3 | 8.575 | 26.4 |
| 5.145 | 3.2 | 6.29 | -3.6 | 7.435 | 16.9 | 8.58 | 10.4 |
| 5.15 | -3.2 | 6.295 | 15.1 | 7.44 | 8.8 | 8.585 | 18.9 |
| 5.155 | -5.6 | 6.3 | 10.6 | 7.445 | 0.8 | 8.59 | 10.9 |
| 5.16 | -23.2 | 6.305 | -0.8 | 7.45 | -2.7 | 8.595 | 27.3 |
| 5.165 | -24.8 | 6.31 | -1.5 | 7.455 | -2.6 | 8.6 | 26.4 |
| 5.17 | 3.2 | 6.315 | 7.4 | 7.46 | -7.7 | 8.605 | 18.9 |
| 5.175 | 13.6 | 6.32 | 1.8 | 7.465 | -10.9 | 8.61 | 18.4 |
| 5.18 | -2.2 | 6.325 | 0.4 | 7.47 | -8.4 | 8.615 | 15.9 |
| 5.185 | 1.6 | 6.33 | 0.2 | 7.475 | -8.6 | 8.62 | 16.8 |
| 5.19 | 2.4 | 6.335 | -7.0 | 7.48 | -5.7 | 8.625 | 16.7 |
| 5.195 | 7.6 | 6.34 | -2.4 | 7.485 | -5.8 | 8.63 | 11.1 |
| 5.2 | 8.0 | 6.345 | 8.2 | 7.49 | -4.3 | 8.635 | 9.3 |
| 5.205 | 8.7 | 6.35 | 16.3 | 7.495 | -9.8 | 8.64 | 8.9 |
| 5.21 | 1.8 | 6.355 | 14.9 | 7.5 | -7.8 | 8.645 | 8.6 |
| 5.215 | 2.4 | 6.36 | 18.0 | 7.505 | -1.5 | 8.65 | 8.2 |
| 5.22 | 2.4 | 6.365 | -3.3 | 7.51 | 2.4 | 8.655 | 7.5 |
| 5.225 | -7.7 | 6.37 | -1.4 | 7.515 | 4.7 | 8.66 | 6.1 |
| 5.23 | -6.2 | 6.375 | 7.6 | 7.52 | 7.3 | 8.665 | 1.6 |
| 5.235 | -4.6 | 6.38 | 10.4 | 7.525 | 11.2 | 8.67 | 2.0 |
| 5.24 | -4.8 | 6.385 | 16.0 | 7.53 | 12.3 | 8.675 | 5.0 |
| 5.245 | -27.2 | 6.39 | 3.7 | 7.535 | 8.2 | 8.68 | 8.1 |
| 5.25 | 2.8 | 6.395 | 5.4 | 7.54 | 5.7 | 8.685 | 2.2 |
| 5.255 | 14.4 | 6.4 | 2.3 | 7.545 | 4.0 | 8.69 | -7.9 |
| 5.26 | 8.4 | 6.405 | -2.5 | 7.55 | 2.5 | 8.695 | -20.8 |
| 5.265 | 5.2 | 6.41 | 6.0 | 7.555 | -1.6 | 8.7 | -9.9 |
| 5.27 | 0.8 | 6.415 | 7.4 | 7.56 | 4.6 | 8.705 | -2.2 |
| 5.275 | -12.8 | 6.42 | 12.3 | 7.565 | 0.8 | 8.71 | 1.3 |
| 5.28 | 2.8 | 6.425 | 12.4 | 7.57 | 3.5 | 8.715 | 6.3 |
| 5.285 | 11.2 | 6.43 | 11.2 | 7.575 | 0.8 | 8.72 | 5.8 |
| 5.29 | 16.8 | 6.435 | 8.1 | 7.58 | 2.5 | 8.725 | 5.7 |
| 5.295 | 3.0 | 6.44 | 7.9 | 7.585 | -7.2 | 8.73 | 12.0 |
| 5.3 | -8.0 | 6.445 | -4.7 | 7.59 | -0.6 | 8.735 | 12.8 |
| 5.305 | 3.2 | 6.45 | -6.6 | 7.595 | -3.0 | 8.74 | 21.3 |
| 5.31 | 7.5 | 6.455 | 4.0 | 7.6 | -8.4 | 8.745 | 18.4 |
| 5.315 | 16.8 | 6.46 | -0.3 | 7.605 | -1.6 | 8.75 | 7.2 |
| 5.32 | 10.4 | 6.465 | 5.5 | 7.61 | 2.1 | 8.755 | 17.3 |
| 5.325 | 22.4 | 6.47 | -1.0 | 7.615 | 11.8 | 8.76 | 19.0 |
| 5.33 | 48.8 | 6.475 | 2.1 | 7.62 | 7.7 | 8.765 | 15.4 |
| 5.335 | 3.8 | 6.48 | 2.4 | 7.625 | -4.8 | 8.77 | 20.0 |
| 5.34 | -2.4 | 6.485 | -1.9 | 7.63 | -6.3 | 8.775 | 4.9 |
| 5.345 | -3.6 | 6.49 | 5.3 | 7.635 | -14.6 | 8.78 | 11.8 |
| 5.35 | 12.0 | 6.495 | -1.0 | 7.64 | -12.1 | 8.785 | 12.3 |
| 5.355 | -22.4 | 6.5 | -3.1 | 7.645 | -15.3 | 8.79 | 20.5 |
| 5.36 | 0.0 | 6.505 | -5.3 | 7.65 | -8.0 | 8.795 | 37.6 |
| 5.365 | -15.2 | 6.51 | -6.2 | 7.655 | -3.3 | 8.8 | 20.0 |
| 5.37 | -2.8 | 6.515 | 6.4 | 7.66 | 5.1 | 8.805 | 33.6 |
| 5.375 | -8.0 | 6.52 | 0.9 | 7.665 | 9.5 | 8.81 | 17.5 |
| 5.38 | -5.8 | 6.525 | -5.9 | 7.67 | 7.1 | 8.815 | 27.2 |
| 5.385 | 8.8 | 6.53 | 5.0 | 7.675 | -0.6 | 8.82 | 7.2 |
| 5.39 | 9.1 | 6.535 | 7.3 | 7.68 | 0.5 | 8.825 | 20.7 |
| 5.395 | 13.6 | 6.54 | 2.6 | 7.685 | -7.3 | 8.83 | 19.5 |
| 5.4 | 17.6 | 6.545 | -11.8 | 7.69 | -5.6 | 8.835 | 19.2 |
| 5.405 | 11.2 | 6.55 | -4.0 | 7.695 | -12.9 | 8.84 | 23.2 |
| 5.41 | -8.8 | 6.555 | 2.2 | 7.7 | -2.3 | 8.845 | -1.6 |
| 5.415 | -1.6 | 6.56 | -2.5 | 7.705 | -0.6 | 8.85 | 18.4 |
| 5.42 | -10.4 | 6.565 | -4.2 | 7.71 | 0.8 | 8.855 | 9.2 |
| 5.425 | 10.4 | 6.57 | -0.8 | 7.715 | 10.5 | 8.86 | 14.1 |
| 5.43 | 13.6 | 6.575 | 0.5 | 7.72 | 6.5 | 8.865 | 20.0 |
| 5.435 | 3.2 | 6.58 | 0.4 | 7.725 | 4.0 | 8.87 | -0.8 |
| 5.44 | 6.4 | 6.585 | 0.7 | 7.73 | 14.1 | 8.875 | 13.9 |
| 5.445 | 8.5 | 6.59 | 7.2 | 7.735 | -4.3 | 8.88 | 29.0 |
| 5.45 | 0.0 | 6.595 | -1.6 | 7.74 | -4.4 | 8.885 | 18.9 |
| 5.455 | -20.0 | 6.6 | -5.9 | 7.745 | -3.9 | 8.89 | 19.2 |
| 5.46 | 38.0 | 6.605 | -4.0 | 7.75 | 0.9 | 8.895 | 12.8 |
| 5.465 | -1.2 | 6.61 | 1.3 | 7.755 | 12.8 | 8.9 | 11.5 |
| 5.47 | 4.8 | 6.615 | -3.5 | 7.76 | 2.2 | 8.905 | 11.7 |
| 5.475 | 38.4 | 6.62 | 4.0 | 7.765 | 2.0 | 8.91 | 16.8 |
| 5.48 | 7.6 | 6.625 | 5.1 | 7.77 | -4.2 | 8.915 | 19.2 |
| 5.485 | 26.4 | 6.63 | 9.9 | 7.775 | 6.2 | 8.92 | 28.0 |
| 5.49 | 0.4 | 6.635 | 16.8 | 7.78 | 7.2 | 8.925 | 26.9 |
| 5.495 | 0.0 | 6.64 | 3.8 | 7.785 | 12.5 | 8.93 | 33.9 |
| 5.5 | -0.8 | 6.645 | -9.0 | 7.79 | 7.2 | 8.935 | 34.4 |
| 5.505 | 3.6 | 6.65 | -8.8 | 7.795 | -3.1 | 8.94 | 26.4 |
| 5.51 | 0.0 | 6.655 | 0.7 | 7.8 | -16.3 | 8.945 | -5.4 |
| 5.515 | -0.8 | 6.66 | -1.8 | 7.805 | -4.9 | 8.95 | -2.8 |
| 5.52 | -7.2 | 6.665 | 6.8 | 7.81 | -14.2 | 8.955 | -0.2 |
| 5.525 | 18.7 | 6.67 | -5.1 | 7.815 | -2.3 | 8.96 | 2.3 |
| 5.53 | 4.0 | 6.675 | -3.9 | 7.82 | -2.0 | 8.965 | 4.9 |
| 5.535 | -10.1 | 6.68 | -0.9 | 7.825 | -2.3 | 8.97 | 7.5 |
| 5.54 | -20.8 | 6.685 | -2.6 | 7.83 | 2.9 | 8.975 | 10.1 |
| 5.545 | -18.4 | 6.69 | -4.3 | 7.835 | 0.0 | 8.98 | 12.6 |
| 5.55 | 4.8 | 6.695 | -1.5 | 7.84 | -2.9 | 8.985 | 15.2 |
| 5.555 | -11.6 | 6.7 | 1.7 | 7.845 | -3.2 | 8.99 | 17.8 |
| 5.56 | -30.4 | 6.705 | 8.2 | 7.85 | 0.1 | 8.995 | 20.3 |
| 5.565 | -16.0 | 6.71 | 7.8 | 7.855 | 1.2 | 9 | 17.2 |
| 5.57 | -35.2 | 6.715 | -0.4 | 7.86 | -0.9 | 9.005 | 16.8 |
| 5.575 | -12.8 | 6.72 | 4.8 | 7.865 | 8.6 | 9.01 | 30.4 |
| 5.58 | -10.4 | 6.725 | 2.3 | 7.87 | 20.4 | 9.015 | 40.0 |
| 5.585 | -19.2 | 6.73 | 4.9 | 7.875 | 13.0 | 9.02 | 40.0 |
| 5.59 | -16.4 | 6.735 | -5.3 | 7.88 | -3.8 | 9.025 | 14.8 |
| 5.595 | -11.2 | 6.74 | 1.6 | 7.885 | -0.9 | 9.03 | 21.6 |
| 5.6 | -20.8 | 6.745 | 3.5 | 7.89 | -6.6 | 9.035 | 16.0 |
| 5.605 | 5.6 | 6.75 | 8.0 | 7.895 | -11.9 | 9.04 | -36.0 |
| 5.61 | -27.2 | 6.755 | 18.0 | 7.9 | 1.4 | 9.045 | -18.9 |
| 5.615 | 1.6 | 6.76 | 23.2 | 7.905 | 4.5 | 9.05 | -22.1 |
| 5.62 | -23.2 | 6.765 | 11.5 | 7.91 | 6.5 | 9.055 | -10.7 |
| 5.625 | -7.2 | 6.77 | 5.7 | 7.915 | 6.2 | 9.06 | -0.8 |
| 5.63 | -16.0 | 6.775 | 4.6 | 7.92 | 0.0 | 9.065 | -0.8 |
| 5.635 | -20.0 | 6.78 | 0.0 | 7.925 | -2.4 | 9.07 | -0.3 |
| 5.64 | -14.4 | 6.785 | -7.7 | 7.93 | 1.1 | 9.075 | 6.4 |
| 5.645 | -15.2 | 6.79 | 2.9 | 7.935 | 2.5 | 9.08 | 0.5 |
| 5.65 | -22.4 | 6.795 | 6.1 | 7.94 | 1.9 | 9.085 | 19.2 |
| 5.655 | -28.8 | 6.8 | -5.2 | 7.945 | -3.0 | 9.09 | -0.5 |
| 5.66 | 5.6 | 6.805 | -1.2 | 7.95 | 3.9 | 9.095 | 27.5 |
| 5.665 | -6.4 | 6.81 | -1.3 | 7.955 | 12.0 | 9.1 | 7.2 |
| 5.67 | -12.0 | 6.815 | 1.9 | 7.96 | -0.1 | 9.105 | 5.1 |
| 5.675 | -32.0 | 6.82 | 16.4 | 7.965 | 4.1 | 9.11 | 8.8 |
| 5.68 | -25.6 | 6.825 | 2.4 | 7.97 | 12.4 | 9.115 | 18.7 |
| 5.685 | -30.9 | 6.83 | 5.4 | 7.975 | 0.3 | 9.12 | 21.6 |
| 5.69 | -36.0 | 6.835 | 0.0 | 7.98 | -1.1 | 9.125 | 8.8 |
| 5.695 | -27.2 | 6.84 | 14.1 | 7.985 | -4.4 | 9.13 | -2.4 |
| 5.7 | -8.8 | 6.845 | 10.5 | 7.99 | -7.6 | 9.135 | -4.8 |
| 5.705 | -15.2 | 6.85 | -0.5 | 7.995 | -5.4 | 9.14 | 13.3 |
| 5.71 | -41.5 | 6.855 | -16.8 | 8 | -4.8 | 9.145 | 20.5 |
| 5.715 | -44.5 | 6.86 | -10.3 | 8.005 | -2.4 | 9.15 | 15.2 |

| **Age (Ma) (*17*)** | **Sea Level (m) (*17*)** | **Age (Ma) (*17*)** | **Sea Level (m) (*17*)** | **Age (Ma) (*17*)** | **Sea Level (m) (*17*)** | **Age (Ma) (*17*)** | **Sea Level (m) (*17*)** |
| --- | --- | --- | --- | --- | --- | --- | --- |
| 9.155 | 8.0 | 30.2 | 3.80 | 53.1 | 80.23 | 76 | 1.39 |
| 9.16 | 4.0 | 30.3 | 3.43 | 53.2 | 83.22 | 76.1 | 16.11 |
| 9.165 | -1.1 | 30.4 | 3.06 | 53.3 | 89.26 | 76.2 | 17.45 |
| 9.17 | 6.0 | 30.5 | 5.61 | 53.4 | 94.32 | 76.3 | 18.89 |
| 9.175 | 13.6 | 30.6 | 8.17 | 53.5 | 91.77 | 76.4 | 20.43 |
| 9.18 | 21.2 | 30.7 | 10.73 | 53.6 | 92.00 | 76.5 | 21.97 |
| 9.185 | -5.6 | 30.8 | 13.29 | 53.7 | 25.00 | 76.6 | 23.50 |
| 9.19 | 24.5 | 30.9 | 15.84 | 53.8 | 26.00 | 76.7 | 25.04 |
| 9.195 | 19.6 | 31 | 16.27 | 53.9 | 38.00 | 76.8 | 24.00 |
| 9.2 | 18.1 | 31.1 | 11.51 | 54 | 54.00 | 76.9 | 15.00 |
| 9.205 | 14.4 | 31.2 | 11.31 | 54.1 | 60.28 | 77 | 11.00 |
| 9.21 | 4.0 | 31.3 | 11.11 | 54.2 | 66.07 | 77.1 | 8.00 |
| 9.215 | 6.4 | 31.4 | 10.89 | 54.3 | 66.85 | 77.2 | 7.00 |
| 9.22 | 8.9 | 31.5 | 10.68 | 54.4 | 67.93 | 77.3 | 8.00 |
| 9.225 | 11.3 | 31.6 | 10.46 | 54.5 | 69.00 | 77.4 | 10.00 |
| 9.23 | 13.7 | 31.7 | 5.00 | 54.6 | 70.08 | 77.5 | 18.00 |
| 9.235 | 16.1 | 31.8 | -4.00 | 54.7 | 63.44 | 77.6 | 26.00 |
| 9.24 | 18.6 | 31.9 | -6.00 | 54.8 | 30.00 | 77.7 | 29.00 |
| 9.245 | 21.0 | 32 | -4.00 | 54.9 | 28.00 | 77.8 | 31.53 |
| 9.25 | 23.4 | 32.1 | 0.00 | 55 | 37.72 | 77.9 | 33.21 |
| 9.3 | -0.77 | 32.2 | 4.68 | 55.1 | 49.80 | 78 | 33.51 |
| 9.4 | -1.03 | 32.3 | -0.26 | 55.2 | 48.22 | 78.1 | 33.81 |
| 9.5 | -12.00 | 32.4 | 31.00 | 55.3 | 46.64 | 78.2 | 34.11 |
| 9.6 | -0.27 | 32.5 | 28.41 | 55.4 | 45.06 | 78.3 | 34.51 |
| 9.7 | -0.73 | 32.6 | 36.48 | 55.5 | 49.44 | 78.4 | 35.50 |
| 9.8 | -4.87 | 32.7 | -8.03 | 55.6 | 43.50 | 78.5 | 36.49 |
| 9.9 | -5.03 | 32.8 | -8.21 | 55.7 | 42.97 | 78.6 | 37.48 |
| 10 | -5.19 | 32.9 | -12.00 | 55.8 | 44.77 | 78.7 | 38.47 |
| 10.1 | -5.35 | 33 | -10.00 | 55.9 | 44.83 | 78.8 | 39.46 |
| 10.2 | -5.51 | 33.1 | 5.86 | 56 | 44.63 | 78.9 | 40.45 |
| 10.3 | -5.66 | 33.2 | 11.22 | 56.1 | 44.40 | 79 | 41.44 |
| 10.4 | -3.97 | 33.3 | 16.59 | 56.2 | 44.18 | 79.1 | 42.43 |
| 10.5 | -4.44 | 33.4 | 21.95 | 56.3 | 43.95 | 79.2 | 43.42 |
| 10.6 | -5.00 | 33.5 | -7.01 | 56.4 | 42.92 | 79.3 | 44.41 |
| 10.7 | -9.00 | 33.6 | 26.00 | 56.5 | 38.62 | 79.4 | 45.40 |
| 10.8 | -10.00 | 33.7 | 40.00 | 56.6 | 33.00 | 79.5 | 46.39 |
| 10.9 | -9.00 | 33.8 | 42.55 | 56.7 | 29.00 | 79.6 | 47.38 |
| 11 | 0.00 | 33.9 | 42.81 | 56.8 | 22.00 | 79.7 | 48.37 |
| 11.1 | 20.00 | 34 | 42.74 | 56.9 | 20.00 | 79.8 | 49.25 |
| 11.2 | 20.00 | 34.1 | 42.67 | 57 | 21.00 | 79.9 | 49.83 |
| 11.3 | 21.12 | 34.2 | 47.07 | 57.1 | 23.00 | 80 | 50.42 |
| 11.4 | 20.01 | 34.3 | 45.00 | 57.2 | 27.00 | 80.1 | 51.01 |
| 11.5 | 18.90 | 34.4 | 30.00 | 57.3 | 32.00 | 80.2 | 51.60 |
| 11.6 | 17.79 | 34.5 | 6.00 | 57.4 | 34.93 | 80.3 | 52.19 |
| 11.7 | 16.68 | 34.6 | 6.00 | 57.5 | 36.93 | 80.4 | 52.78 |
| 11.8 | 15.57 | 34.7 | 15.00 | 57.6 | 38.92 | 80.5 | 53.37 |
| 11.9 | 14.46 | 34.8 | 17.48 | 57.7 | 40.92 | 80.6 | 53.96 |
| 12 | -1.86 | 34.9 | 29.55 | 57.8 | 42.92 | 80.7 | 54.55 |
| 12.1 | -1.98 | 35 | 34.25 | 57.9 | 46.64 | 80.8 | 55.14 |
| 12.2 | -2.10 | 35.1 | 38.95 | 58 | 43.33 | 80.9 | 45.44 |
| 12.3 | -2.23 | 35.2 | 41.59 | 58.1 | 48.57 | 81 | 46.04 |
| 12.4 | -3.08 | 35.3 | 45.19 | 58.2 | 48.12 | 81.1 | 46.00 |
| 12.5 | -3.13 | 35.4 | 41.41 | 58.3 | 47.00 | 81.2 | 45.50 |
| 12.6 | -12.00 | 35.5 | 54.34 | 58.4 | 45.88 | 81.3 | 45.00 |
| 12.7 | 5.48 | 35.6 | 32.17 | 58.5 | 44.76 | 81.4 | 43.00 |
| 12.8 | -1.71 | 35.7 | 49.18 | 58.6 | 49.70 | 81.5 | 40.00 |
| 12.9 | 2.50 | 35.8 | 49.00 | 58.7 | 49.69 | 81.6 | 36.00 |
| 13 | 2.00 | 35.9 | 35.00 | 58.8 | 49.69 | 81.7 | 32.00 |
| 13.1 | -7.00 | 36 | 22.00 | 58.9 | 49.68 | 81.8 | 28.00 |
| 13.2 | -8.00 | 36.1 | 10.00 | 59 | 49.67 | 81.9 | 24.00 |
| 13.3 | -7.00 | 36.2 | 8.00 | 59.1 | 49.66 | 82 | 19.00 |
| 13.4 | -1.00 | 36.3 | 12.00 | 59.2 | 49.65 | 82.1 | 16.00 |
| 13.5 | -0.08 | 36.4 | 17.00 | 59.3 | 49.65 | 82.2 | 14.00 |
| 13.6 | -0.20 | 36.5 | 19.00 | 59.4 | 49.64 | 82.3 | 13.00 |
| 13.7 | -1.17 | 36.6 | 19.27 | 59.5 | 49.63 | 82.4 | 12.00 |
| 13.8 | -2.13 | 36.7 | 19.09 | 59.6 | 49.62 | 82.5 | 12.00 |
| 13.9 | -1.50 | 36.8 | 26.13 | 59.7 | 49.61 | 82.6 | 13.00 |
| 14 | -9.00 | 36.9 | 25.65 | 59.8 | 50.16 | 82.7 | 14.00 |
| 14.1 | -10.00 | 37 | 25.17 | 59.9 | 50.25 | 82.8 | 16.00 |
| 14.2 | -9.00 | 37.1 | 43.55 | 60 | 50.34 | 82.9 | 19.00 |
| 14.3 | -1.00 | 37.2 | 40.00 | 60.1 | 40.00 | 83 | 25.00 |
| 14.4 | -0.59 | 37.3 | 34.00 | 60.2 | 32.00 | 83.1 | 32.00 |
| 14.5 | -2.72 | 37.4 | 30.00 | 60.3 | 29.00 | 83.2 | 41.15 |
| 14.6 | -4.85 | 37.5 | 26.00 | 60.4 | 26.00 | 83.3 | 54.42 |
| 14.7 | -9.40 | 37.6 | 23.00 | 60.5 | 27.00 | 83.4 | 56.11 |
| 14.8 | -10.00 | 37.7 | 21.00 | 60.6 | 29.00 | 83.5 | 57.80 |
| 14.9 | -11.00 | 37.8 | 20.00 | 60.7 | 32.00 | 83.6 | 47.10 |
| 15 | -12.00 | 37.9 | 19.00 | 60.8 | 42.00 | 83.7 | 48.58 |
| 15.1 | -14.00 | 38 | 18.00 | 60.9 | 48.00 | 83.8 | 42.65 |
| 15.2 | -10.00 | 38.1 | 17.00 | 61 | 50.00 | 83.9 | 47.52 |
| 15.3 | 0.00 | 38.2 | 15.00 | 61.1 | 51.75 | 84 | 40.00 |
| 15.4 | 10.00 | 38.3 | 13.00 | 61.2 | 51.86 | 84.1 | -5.00 |
| 15.5 | 15.00 | 38.4 | 11.00 | 61.3 | 45.72 | 84.2 | -3.00 |
| 15.6 | 16.88 | 38.5 | 9.00 | 61.4 | 45.87 | 84.3 | 0.77 |
| 15.7 | 5.80 | 38.6 | 7.50 | 61.5 | 46.02 | 84.4 | 25.34 |
| 15.8 | 1.86 | 38.7 | 6.00 | 61.6 | 46.18 | 84.5 | 25.29 |
| 15.9 | 1.40 | 38.8 | 5.50 | 61.7 | 46.33 | 84.6 | 25.24 |
| 16 | 0.93 | 38.9 | 5.00 | 61.8 | 52.51 | 84.7 | 25.19 |
| 16.1 | 0.46 | 39 | 5.50 | 61.9 | 52.62 | 84.8 | 25.14 |
| 16.2 | -14.00 | 39.1 | 6.00 | 62 | 52.73 | 84.9 | 25.09 |
| 16.3 | -16.00 | 39.2 | 6.50 | 62.1 | 52.84 | 85 | 25.05 |
| 16.4 | -14.00 | 39.3 | 7.00 | 62.2 | 52.95 | 85.1 | 25.00 |
| 16.5 | 3.10 | 39.4 | 8.00 | 62.3 | 51.02 | 85.2 | 24.95 |
| 16.6 | 7.22 | 39.5 | 9.00 | 62.4 | 51.94 | 85.3 | 26.00 |
| 16.7 | 9.66 | 39.6 | 12.00 | 62.5 | 52.87 | 85.4 | 24.00 |
| 16.8 | 8.49 | 39.7 | 15.00 | 62.6 | 53.80 | 85.5 | 22.00 |
| 16.9 | 6.79 | 39.8 | 19.00 | 62.7 | 54.72 | 85.6 | 19.00 |
| 17 | 6.26 | 39.9 | 22.00 | 62.8 | 55.38 | 85.7 | 17.00 |
| 17.1 | 5.72 | 40 | 17.00 | 62.9 | 54.66 | 85.8 | 15.00 |
| 17.2 | 7.09 | 40.1 | 18.03 | 63 | 53.93 | 85.9 | 12.00 |
| 17.3 | 5.25 | 40.2 | 18.14 | 63.1 | 50.00 | 86 | 9.00 |
| 17.4 | 5.24 | 40.3 | 18.25 | 63.2 | 36.00 | 86.1 | 7.00 |
| 17.5 | 4.15 | 40.4 | 18.36 | 63.3 | 25.00 | 86.2 | 6.00 |
| 17.6 | -2.05 | 40.5 | 18.47 | 63.4 | 19.00 | 86.3 | 5.00 |
| 17.7 | -1.76 | 40.6 | 30.71 | 63.5 | 11.00 | 86.4 | 6.00 |
| 17.8 | 10.14 | 40.7 | 31.69 | 63.6 | 9.00 | 86.5 | 7.00 |
| 17.9 | 8.00 | 40.8 | 32.68 | 63.7 | 8.00 | 86.6 | 10.00 |
| 18 | -17.00 | 40.9 | 33.67 | 63.8 | 8.00 | 86.7 | 25.00 |
| 18.1 | -18.00 | 41 | 34.66 | 63.9 | 9.00 | 86.8 | 14.55 |
| 18.2 | -16.00 | 41.1 | 25.42 | 64 | 10.00 | 86.9 | 14.66 |
| 18.3 | -12.28 | 41.2 | 25.29 | 64.1 | 12.00 | 87 | 14.77 |
| 18.4 | -12.22 | 41.3 | 24.00 | 64.2 | 15.00 | 87.1 | 14.88 |
| 18.5 | -12.15 | 41.4 | 22.00 | 64.3 | 22.00 | 87.2 | 14.99 |
| 18.6 | -12.09 | 41.5 | 20.00 | 64.4 | 25.00 | 87.3 | 15.10 |
| 18.7 | -12.02 | 41.6 | 18.00 | 64.5 | 22.00 | 87.4 | 15.22 |
| 18.8 | -12.08 | 41.7 | 16.00 | 64.6 | 23.60 | 87.5 | 15.33 |
| 18.9 | -12.17 | 41.8 | 14.00 | 64.7 | 25.07 | 87.6 | 15.44 |
| 19 | -12.26 | 41.9 | 11.00 | 64.8 | 26.55 | 87.7 | 15.55 |
| 19.1 | -12.34 | 42 | 8.00 | 64.9 | 28.02 | 87.8 | 15.66 |
| 19.2 | -12.43 | 42.1 | 6.00 | 65 | 29.49 | 87.9 | 14.00 |
| 19.3 | -7.90 | 42.2 | 5.00 | 65.1 | 30.96 | 88 | 7.00 |
| 19.4 | -13.64 | 42.3 | 5.00 | 65.2 | 32.43 | 88.1 | 10.00 |
| 19.5 | -1.59 | 42.4 | 6.00 | 65.3 | 33.91 | 88.2 | 16.00 |
| 19.6 | 7.15 | 42.5 | 7.00 | 65.4 | 35.38 | 88.3 | 27.00 |
| 19.7 | 7.72 | 42.6 | 9.00 | 65.5 | 36.85 | 88.4 | 29.33 |
| 19.8 | 2.86 | 42.7 | 15.00 | 65.6 | 38.32 | 88.5 | 29.12 |
| 19.9 | 1.94 | 42.8 | 25.00 | 65.7 | 39.80 | 88.6 | 28.91 |
| 20 | 3.59 | 42.9 | 32.00 | 65.8 | 41.27 | 88.7 | 28.70 |
| 20.1 | -1.07 | 43 | 43.00 | 65.9 | 42.74 | 88.8 | 28.49 |
| 20.2 | -3.57 | 43.1 | 52.00 | 66 | 44.21 | 88.9 | 28.28 |
| 20.3 | 0.80 | 43.2 | 58.00 | 66.1 | 42.00 | 89 | 28.07 |
| 20.4 | 1.25 | 43.3 | 62.00 | 66.2 | 38.00 | 89.1 | 27.86 |
| 20.5 | 2.53 | 43.4 | 63.00 | 66.3 | 25.00 | 89.2 | 27.65 |
| 20.6 | 1.49 | 43.5 | 64.55 | 66.4 | 19.00 | 89.3 | 27.44 |
| 20.7 | 4.09 | 43.6 | 64.52 | 66.5 | 15.00 | 89.4 | 27.22 |
| 20.8 | 3.62 | 43.7 | 64.50 | 66.6 | 14.00 | 89.5 | 27.01 |
| 20.9 | 3.14 | 43.8 | 72.26 | 66.7 | 16.00 | 89.6 | 26.80 |
| 21 | 2.66 | 43.9 | 73.10 | 66.8 | 20.00 | 89.7 | 26.59 |
| 21.1 | 3.39 | 44 | 73.95 | 66.9 | 33.00 | 89.8 | 26.38 |
| 21.2 | 7.40 | 44.1 | 74.80 | 67 | 38.00 | 89.9 | 2.00 |
| 21.3 | -14.45 | 44.2 | 75.62 | 67.1 | 38.76 | 90 | 10.31 |
| 21.4 | -13.02 | 44.3 | 76.44 | 67.2 | 27.38 | 90.1 | 10.57 |
| 21.5 | -12.52 | 44.4 | 77.26 | 67.3 | 28.66 | 90.2 | 10.82 |
| 21.6 | -13.76 | 44.5 | 66.52 | 67.4 | 29.94 | 90.3 | 11.07 |
| 21.7 | -18.00 | 44.6 | 58.00 | 67.5 | 31.23 | 90.4 | 11.32 |
| 21.8 | -20.00 | 44.7 | 51.00 | 67.6 | 32.51 | 90.5 | 11.57 |
| 21.9 | -18.00 | 44.8 | 47.00 | 67.7 | 33.79 | 90.6 | 11.82 |
| 22 | -10.96 | 44.9 | 44.00 | 67.8 | 35.07 | 90.7 | 12.07 |
| 22.1 | -10.00 | 45 | 40.00 | 67.9 | 36.36 | 90.8 | 12.32 |
| 22.2 | -9.04 | 45.1 | 38.00 | 68 | 37.64 | 90.9 | 12.57 |
| 22.3 | -8.09 | 45.2 | 37.00 | 68.1 | 38.92 | 91 | 12.82 |
| 22.4 | -7.13 | 45.3 | 37.00 | 68.2 | 40.20 | 91.1 | 13.07 |
| 22.5 | -6.18 | 45.4 | 38.00 | 68.3 | 41.49 | 91.2 | 13.32 |
| 22.6 | -5.22 | 45.5 | 40.00 | 68.4 | 42.77 | 91.3 | 13.57 |
| 22.7 | -4.26 | 45.6 | 50.00 | 68.5 | 44.05 | 91.4 | 13.83 |
| 22.8 | -3.31 | 45.7 | 55.00 | 68.6 | 45.34 | 91.5 | 12.00 |
| 22.9 | -2.35 | 45.8 | 60.00 | 68.7 | 46.62 | 91.6 | 5.00 |
| 23 | -1.40 | 45.9 | 65.00 | 68.8 | 35.58 | 91.7 | 2.00 |
| 23.1 | -0.44 | 46 | 70.00 | 68.9 | 36.89 | 91.8 | 4.00 |
| 23.2 | -0.58 | 46.1 | 75.00 | 69 | 38.19 | 91.9 | 10.00 |
| 23.3 | -3.62 | 46.2 | 76.33 | 69.1 | 46.00 | 92 | 22.00 |
| 23.4 | 5.58 | 46.3 | 75.00 | 69.2 | 44.00 | 92.1 | 28.13 |
| 23.5 | 4.01 | 46.4 | 70.00 | 69.3 | 40.00 | 92.2 | 38.31 |
| 23.6 | -0.78 | 46.5 | 65.00 | 69.4 | 37.00 | 92.3 | 27.09 |
| 23.7 | -9.21 | 46.6 | 56.00 | 69.5 | 30.00 | 92.4 | 26.58 |
| 23.8 | -17.64 | 46.7 | 47.00 | 69.6 | 25.00 | 92.5 | 29.23 |
| 23.9 | -31.51 | 46.8 | 43.00 | 69.7 | 21.00 | 92.6 | 32.04 |
| 24 | -32.21 | 46.9 | 45.00 | 69.8 | 18.00 | 92.7 | 34.59 |
| 24.1 | -32.91 | 47 | 48.00 | 69.9 | 16.00 | 92.8 | 37.12 |
| 24.2 | -23.38 | 47.1 | 55.00 | 70 | 13.00 | 92.9 | 41.59 |
| 24.3 | -36.08 | 47.2 | 60.00 | 70.1 | 11.00 | 93 | 43.28 |
| 24.4 | -28.52 | 47.3 | 62.33 | 70.2 | 10.00 | 93.1 | 44.97 |
| 24.5 | -10.62 | 47.4 | 60.00 | 70.3 | 11.00 | 93.2 | 46.65 |
| 24.6 | -11.75 | 47.5 | 58.00 | 70.4 | 12.00 | 93.3 | 48.82 |
| 24.7 | -12.90 | 47.6 | 55.00 | 70.5 | 15.00 | 93.4 | 51.50 |
| 24.8 | -33.86 | 47.7 | 52.00 | 70.6 | 20.00 | 93.5 | 48.00 |
| 24.9 | 12.87 | 47.8 | 51.00 | 70.7 | 23.00 | 93.6 | 44.77 |
| 25 | 11.61 | 47.9 | 52.00 | 70.8 | 25.00 | 93.7 | 46.85 |
| 25.1 | 1.94 | 48 | 53.00 | 70.9 | 26.00 | 93.8 | 48.94 |
| 25.2 | -7.08 | 48.1 | 55.00 | 71 | 3.00 | 93.9 | 51.03 |
| 25.3 | -16.09 | 48.2 | 61.00 | 71.1 | 5.00 | 94 | 53.11 |
| 25.4 | -6.97 | 48.3 | 65.00 | 71.2 | 14.73 | 94.1 | 55.20 |
| 25.5 | 2.15 | 48.4 | 69.25 | 71.3 | 28.44 | 94.2 | 57.28 |
| 25.6 | 1.30 | 48.5 | 74.79 | 71.4 | 28.62 | 94.3 | 59.37 |
| 25.7 | 0.45 | 48.6 | 73.93 | 71.5 | 28.80 | 94.4 | 61.46 |
| 25.8 | -12.04 | 48.7 | 71.00 | 71.6 | 28.98 | 94.5 | 63.54 |
| 25.9 | -9.97 | 48.8 | 62.00 | 71.7 | 29.16 | 94.6 | 38.00 |
| 26 | -7.90 | 48.9 | 58.00 | 71.8 | 29.34 | 94.7 | 47.74 |
| 26.1 | -5.83 | 49 | 52.00 | 71.9 | 29.53 | 94.8 | 51.70 |
| 26.2 | -3.77 | 49.1 | 50.00 | 72 | 29.71 | 94.9 | 55.30 |
| 26.3 | 10.69 | 49.2 | 52.00 | 72.1 | 29.89 | 95 | 58.89 |
| 26.4 | 25.14 | 49.3 | 65.00 | 72.2 | 30.07 | 95.1 | 61.43 |
| 26.5 | 23.14 | 49.4 | 73.00 | 72.3 | 30.25 | 95.2 | 62.83 |
| 26.6 | 2.72 | 49.5 | 79.00 | 72.4 | 30.43 | 95.3 | 64.24 |
| 26.7 | 38.79 | 49.6 | 80.81 | 72.5 | 30.61 | 95.4 | 65.65 |
| 26.8 | 18.39 | 49.7 | 80.64 | 72.6 | 30.79 | 95.5 | 67.05 |
| 26.9 | 2.47 | 49.8 | 60.93 | 72.7 | 30.97 | 95.6 | 68.46 |
| 27 | -14.58 | 49.9 | 61.25 | 72.8 | 31.15 | 95.7 | 69.87 |
| 27.1 | -2.17 | 50 | 58.92 | 72.9 | 31.33 | 95.8 | 71.27 |
| 27.2 | -10.47 | 50.1 | 59.92 | 73 | 31.51 | 95.9 | 70.00 |
| 27.3 | 17.83 | 50.2 | 60.92 | 73.1 | 35.75 | 96 | 66.00 |
| 27.4 | 16.08 | 50.3 | 61.93 | 73.2 | 36.12 | 96.1 | 60.00 |
| 27.5 | 14.39 | 50.4 | 62.93 | 73.3 | 36.48 | 96.2 | 54.00 |
| 27.6 | -4.67 | 50.5 | 27.00 | 73.4 | 36.85 | 96.3 | 44.00 |
| 27.7 | -23.73 | 50.6 | 26.00 | 73.5 | 37.21 | 96.4 | 75.00 |
| 27.8 | -24.41 | 50.7 | 36.95 | 73.6 | 37.58 | 96.5 | 70.00 |
| 27.9 | -26.45 | 50.8 | 55.25 | 73.7 | 37.94 | 96.6 | 40.00 |
| 28 | -30.00 | 50.9 | 56.71 | 73.8 | 38.31 | 96.7 | 30.00 |
| 28.1 | -32.00 | 51 | 53.43 | 73.9 | 38.68 | 96.8 | 27.00 |
| 28.2 | -20.00 | 51.1 | 53.95 | 74 | 39.04 | 96.9 | 30.00 |
| 28.3 | 19.50 | 51.2 | 54.47 | 74.1 | 39.41 | 97 | 37.00 |
| 28.4 | 18.97 | 51.3 | 54.99 | 74.2 | 39.77 | 97.1 | 38.47 |
| 28.5 | 18.43 | 51.4 | 55.51 | 74.3 | 41.15 | 97.2 | 38.67 |
| 28.6 | 19.09 | 51.5 | 59.88 | 74.4 | 43.29 | 97.3 | 38.87 |
| 28.7 | 18.64 | 51.6 | 64.53 | 74.5 | 44.93 | 97.4 | 40.76 |
| 28.8 | 5.62 | 51.7 | 69.18 | 74.6 | 45.38 | 97.5 | 48.67 |
| 28.9 | -7.40 | 51.8 | 73.84 | 74.7 | 43.24 | 97.6 | 56.34 |
| 29 | 7.82 | 51.9 | 76.74 | 74.8 | 41.11 | 97.7 | 54.14 |
| 29.1 | 3.72 | 52 | 77.34 | 74.9 | 38.97 | 97.8 | 51.94 |
| 29.2 | -1.84 | 52.1 | 87.96 | 75 | 36.84 | 97.9 | 49.74 |
| 29.3 | -7.41 | 52.2 | 91.61 | 75.1 | 33.20 | 98 | 39.13 |
| 29.4 | -8.78 | 52.3 | 100.12 | 75.2 | 32.43 | 98.1 | 37.99 |
| 29.5 | -9.00 | 52.4 | 121.87 | 75.3 | 31.66 | 98.2 | 36.86 |
| 29.6 | -12.00 | 52.5 | 124.56 | 75.4 | 30.89 | 98.3 | 35.73 |
| 29.7 | -13.00 | 52.6 | 127.26 | 75.5 | 30.12 | 98.4 | 35.74 |
| 29.8 | -10.00 | 52.7 | 129.96 | 75.6 | 29.36 | 98.96 | 26.37 |
| 29.9 | 0.00 | 52.8 | 132.65 | 75.7 | 28.59 | 99.03 | 26.1 |
| 30 | 3.00 | 52.9 | 77.71 | 75.8 | 0.80 | 99.15 | 21.63 |
| 30.1 | 4.18 | 53 | 78.96 | 75.9 | 1.09 | 99.28 | 12.54 |
| **Age (Ma) (17)** | **Sea Level (m) (17)** | **Age (Ma) (17)** | **Sea Level (m) (17)** | **Age (Ma) (17)** | **Sea Level (m) (17)** | **Age (Ma) (17)** | **Sea Level (m) (17)** |
| 99.34 | 0.18 | 103.85 | 7.37 | 110.04 | -2.6 | 114.95 | 1.41 |
| 99.46 | 1.61 | 103.96 | 5.76 | 110.24 | -2.36 | 115.09 | 3.54 |
| 99.59 | 14.42 | 104.16 | 4.39 | 110.34 | -1.58 | 115.23 | 5.42 |
| 99.72 | 26.56 | 104.37 | 3.04 | 110.55 | 0.03 | 115.30 | 7.11 |
| 99.78 | 34.66 | 104.57 | 2.74 | 110.76 | 1.31 | 115.44 | 8.29 |
| 99.90 | 38.69 | 104.68 | 3.14 | 110.86 | 2.02 | 115.58 | 8.94 |
| 100.03 | 41.9 | 104.88 | 3.86 | 111.07 | 2.91 | 115.65 | 9.66 |
| 100.09 | 45.19 | 105.09 | 4.51 | 111.27 | 4.48 | 115.79 | 10.51 |
| 100.22 | 48.26 | 105.19 | 5.05 | 111.48 | 5.07 | 115.93 | 11.08 |
| 100.34 | 50.44 | 105.40 | 5.81 | 111.58 | 3.79 | 116.07 | 11.08 |
| 100.47 | 50.19 | 105.60 | 6.4 | 111.79 | -0.97 | 116.14 | 9.1 |
| 100.53 | 46.7 | 105.81 | 7 | 111.99 | -5.65 | 116.28 | 6.2 |
| 100.66 | 42.3 | 105.91 | 7.41 | 112.20 | -9.6 | 116.42 | 3.46 |
| 100.78 | 38.89 | 106.12 | 6.97 | 112.27 | -13.53 | 116.49 | 0.75 |
| 100.91 | 35.48 | 106.33 | 4.66 | 112.41 | -17.79 | 116.64 | 0.68 |
| 100.97 | 32.11 | 106.43 | 1.38 | 112.55 | -22.06 | 116.78 | 4.09 |
| 101.10 | 29.13 | 106.63 | -2.37 | 112.62 | -25.41 | 116.92 | 7.06 |
| 101.22 | 26.14 | 106.84 | -5.63 | 112.76 | -28.33 | 116.99 | 10.56 |
| 101.28 | 23.48 | 107.05 | -8.14 | 112.90 | -30.74 | 117.13 | 13.11 |
| 101.41 | 20.5 | 107.15 | -8.6 | 113.04 | -32.62 | 117.27 | 15.24 |
| 101.54 | 17.17 | 107.36 | -8.51 | 113.12 | -33.97 | 117.34 | 16.08 |
| 101.66 | 12.25 | 107.56 | -8.09 | 113.26 | -34.83 | 117.48 | 15.59 |
| 101.72 | 5.62 | 107.77 | -7.38 | 113.40 | -35.29 | 117.62 | 11.75 |
| 101.85 | -0.59 | 107.87 | -6.86 | 113.47 | -35 | 117.76 | 8.11 |
| 101.97 | -2.93 | 108.08 | -6.01 | 113.61 | -33.44 | 117.83 | 4.11 |
| 102.04 | 0.14 | 108.28 | -5.38 | 113.75 | -31.45 | 117.97 | 0.4 |
| 102.20 | 4.8 | 108.39 | -4.64 | 113.89 | -28.66 | 118.11 | -2.16 |
| 102.41 | 9.49 | 108.59 | -4.12 | 113.96 | -25.67 | 118.25 | -3.99 |
| 102.62 | 13.68 | 108.80 | -3.26 | 114.10 | -22.69 | 118.32 | -4.94 |
| 102.72 | 15.41 | 109.01 | -2.67 | 114.24 | -19.52 | 118.47 | -5.79 |
| 102.92 | 15 | 109.11 | -2.6 | 114.31 | -15.84 | 118.61 | -6.64 |
| 103.13 | 13.49 | 109.31 | -2.6 | 114.45 | -11.7 | 118.68 | -7.5 |
| 103.34 | 11.92 | 109.52 | -2.6 | 114.59 | -8.29 | 118.82 | -8.35 |
| 103.44 | 10.55 | 109.62 | -2.6 | 114.73 | -4.88 | 118.96 | -9.2 |
| 103.65 | 8.84 | 109.83 | -2.6 | 114.80 | -1.15 | 119.10 | -9.83 |
| **Age (Ma) (17)** | **Sea Level (m) (17)** | **Age (Ma) (17)** | **Sea Level (m) (17)** | **Age (Ma) (17)** | **Sea Level (m) (17)** | **Age (Ma) (17)** | **Sea Level (m) (17)** |
| 119.17 | -10.26 | 123.80 | 2.21 | 130.33 | -15.75 | 136.55 | -32.55 |
| 119.31 | -11.35 | 123.88 | 3.06 | 130.50 | -17.94 | 136.73 | -28.02 |
| 119.45 | -12.12 | 124.04 | 3.83 | 130.83 | -16.91 | 136.82 | -23.5 |
| 119.52 | -13.4 | 124.20 | 4.07 | 131.17 | -12.36 | 137.00 | -18.86 |
| 119.66 | -14.7 | 124.28 | 4.34 | 131.50 | -9.27 | 137.29 | -15.98 |
| 119.80 | -16.64 | 124.44 | 4.74 | 131.67 | -13.79 | 137.58 | -13.62 |
| 119.94 | -19.2 | 124.60 | 4.74 | 132.00 | -24.03 | 137.72 | -12.94 |
| 120.01 | -21.45 | 124.76 | 4.96 | 132.18 | -30.77 | 138.01 | -13.33 |
| 120.16 | -23.62 | 124.84 | 5.07 | 132.27 | -32.96 | 138.30 | -13.9 |
| 120.30 | -23.46 | 125.00 | 4.74 | 132.45 | -30.07 | 138.44 | -14.75 |
| 120.44 | -22.54 | 125.16 | 4.58 | 132.64 | -26.65 | 138.73 | -15.6 |
| 120.51 | -21.27 | 125.24 | 4.41 | 132.82 | -24.44 | 139.02 | -16.54 |
| 120.65 | -20.42 | 125.40 | 4.39 | 132.91 | -24.28 | 139.30 | -18.01 |
| 120.79 | -19.56 | 125.56 | 4.07 | 133.09 | -25 | 139.45 | -20.26 |
| 120.86 | -18.71 | 125.72 | 4.07 | 133.27 | -25.62 | 139.74 | -22.28 |
| 121.00 | -17.52 | 125.80 | 3.78 | 133.45 | -24.56 | 140.02 | -21.77 |
| 121.16 | -16.67 | 125.96 | 3.74 | 133.55 | -23.04 | 140.17 | -19.41 |
| 121.32 | -15.82 | 126.12 | 3.59 | 133.73 | -23.26 | 140.46 | -17.99 |
| 121.40 | -14.96 | 126.20 | 3.4 | 133.91 | -24.62 | 140.74 | -18.38 |
| 121.56 | -13.95 | 126.36 | 3.74 | 134.00 | -20.64 | 141.03 | -20.94 |
| 121.72 | -12.59 | 126.52 | 4.59 | 134.18 | -19.29 | 141.18 | -22.46 |
| 121.80 | -11.74 | 126.68 | 5.63 | 134.36 | -20.82 | 141.46 | -22.53 |
| 121.96 | -10.88 | 126.76 | 6.63 | 134.55 | -25.75 | 141.75 | -20.29 |
| 122.12 | -9.79 | 126.92 | 7.48 | 134.64 | -27.29 | 142.04 | -18.13 |
| 122.28 | -8.51 | 127.17 | 8.98 | 134.82 | -25.28 | 142.18 | -17.61 |
| 122.36 | -7.66 | 127.50 | 10.41 | 135.00 | -25.7 | 142.47 | -18.06 |
| 122.52 | -6.67 | 127.67 | 10.41 | 135.09 | -29.53 | 142.76 | -18.91 |
| 122.68 | -5.29 | 128.00 | 3.38 | 135.27 | -36.15 | 142.90 | -19.52 |
| 122.76 | -4.18 | 128.33 | -3.6 | 135.45 | -35.79 | 143.19 | -19.95 |
| 122.92 | -2.91 | 128.50 | -3.6 | 135.64 | -32.52 | 143.48 | -20.47 |
| 123.08 | -2.06 | 128.83 | -9.63 | 135.73 | -30.95 | 143.77 | -21.51 |
| 123.24 | -1.21 | 129.17 | -16.07 | 135.91 | -33.86 | 143.91 | -22.79 |
| 123.32 | -0.35 | 129.50 | -19.46 | 136.09 | -36.9 | 144.20 | -22.91 |
| 123.48 | 0.5 | 129.67 | -20.61 | 136.18 | -37.96 | 144.40 | -21.56 |
| 123.64 | 1.35 | 130.00 | -13.94 | 136.36 | -35.8 | 144.50 | -17.48 |

| **Age (Ma) (*17*)** | **Sea Level (m) (*17*)** | **Age (Ma) (*17*)** | **Sea Level (m) (*17*)** |
| --- | --- | --- | --- |
| 144.70 | -14.37 | 150.70 | 4.74 |
| 144.90 | -13.27 | 151.04 | 4.74 |
| 145.10 | -13.44 | 151.38 | 4.41 |
| 145.20 | -13.87 | 151.72 | 4.41 |
| 145.40 | -13.92 | 151.89 | 4.41 |
| 145.60 | -13.49 | 152.23 | 3.85 |
| 145.70 | -13.07 | 152.57 | -1.62 |
| 145.90 | -12.64 | 152.74 | -12.7 |
| 146.10 | -12.73 | 153.08 | -15.61 |
| 146.30 | -13.58 | 153.42 | -12.43 |
| 146.40 | -14.43 | 153.76 | -4.85 |
| 146.60 | -15.61 | 153.93 | 1.98 |
| 146.80 | -15.51 | 154.28 | 4.07 |
| 147.00 | -14.7 | 154.63 | 3.96 |
| 147.10 | -13.42 | 154.81 | 3.54 |
| 147.30 | -11.94 | 155.16 | 2.82 |
| 147.50 | -12.21 | 155.51 | -6.97 |
| 147.60 | -15.82 | 155.87 | -8.94 |
| 147.80 | -17.28 | 156.04 | -8.22 |
| 148.00 | -15.07 | 156.40 | -7.61 |
| 148.20 | -3.51 | 156.75 | 1.07 |
| 148.30 | 1.74 | 156.93 | -0.77 |
| 148.50 | -10.03 | 157.28 | -7.8 |
| 148.70 | -23.93 | 157.63 | -19.11 |
| 148.80 | -26.28 | 157.99 | -25.95 |
| 149.00 | -23.84 | 158.16 | -20.18 |
| 149.20 | -10 | 158.52 | -7.83 |
| 149.40 | 0.66 | 158.87 | -6.27 |
| 149.50 | 2.82 | 159.22 | -7.27 |
| 149.70 | 4.08 | 159.40 | -11.39 |
| 149.90 | 4.51 | 159.62 | -16.43 |
| 150.00 | 4.74 | 159.84 | -19.73 |
| 150.20 | 4.74 | 159.96 | -22.31 |
| 150.40 | 4.74 |  |  |
| 150.60 | 4.74 |  |  |

| **Supplementary Table S2. ALL DATA ON 11 MYR BINS ONLY NO DATA DIFFERENCED.*** | | | | | |
| --- | --- | --- | --- | --- | --- |
| **Age (Ma) (Foster et al.)** | **CO_2_ (ppm) (Foster et al.)** | **^87^Sr/^86^Sr (Cárdenas & Harries)** | **Biogenic Phosphorus Accumulation Rate (Föllmi)** | **Total Phosphorus Accumulation Rate (PAR) (Föllmi)** | **Nannofossil Diversification Rate (Rs-Re) (Bown et al.)** |
| 17 | 261 | 0.7087 | 0.13751188 | 0.50177838 | -4.816 |
| 28 | 458.8 | 0.7081 | 0.404236295 | 0.386114995 | -8.724097756 |
| 39 | 568.3333333 | 0.7078 | 0.14246679 | 0.29011654 | -2.728878788 |
| 50 | 692.7272727 | 0.7078 | 0.3949974 | 0.516051265 | 10.6824177 |
| 61 | 24.31034483 | 0.7079 | 0.800355665 | 0.64368086 | 22.4883344 |
| 72 | 307.5272727 | 0.7078 | 0.195710515 | 0.1801596 | -5.230433194 |
| 83 | 1522 | 0.7075 | 0.35383624 | 0.651164335 | 5.46542791 |
| 94 | 1082.645161 | 0.7074 | 0.47655943 | 0.63489468 | -2.040848718 |
| 105 | 1276.307692 | 0.7074 |  | 0.24446645 | 3.20356497 |
| 116 | 1071 | 0.7073 |  | 0.508331145 | 7.104089356 |
| 127 | 1596.222222 | 0.7074 |  | 0.314381215 | -3.832255828 |
| 138 | 182.9767442 | 0.7074 |  | 0.547557785 | 13.28181658 |
| 149 | 742.7142857 | 0.7072 |  | 0.39200084 | 3.805469401 |
| 160 | 590.0833333 | 0.707 |  |  | 4.706858974 |
|  |  |  |  |  |  |
|  |  |  |  |  |  |
| **Supplementary Table S2 (continued)** | | |  |  |  |
| **Age (Ma) (Foster et al.)** | **δ^13^C (Cárdenas & Harries)** | **δ^34^S (Cárdenas & Harries)** | **Origination Rate (Cárdenas & Harries)** | **Sea Level (Cárdenas & Harries)** |  |
| 17 | 2.0508 | 22.156 | 0.2543 | 45.7793 |  |
| 28 | 1.5018 | 22 | 0.2702 | 50.5386 |  |
| 39 | 1.6927 | 21.8392 | 0.2062 | 56.0559 |  |
| 50 | 2.6273 | 18.9667 | 0.3305 | 71.2126 |  |
| 61 | 2.1929 | 18.1882 | 0.7747 | 66.1895 |  |
| 72 | 1.6296 | 18.3551 | 0.2461 | 56.8777 |  |
| 83 | 1.5036 | 17.9 | 0.3296 | 61.8653 |  |
| 94 | 1.3332 | 16.59 | 0.3147 | 67.3721 |  |
| 105 | 2.6984 | 14.5 | 0.3658 | 49.7767 |  |
| 116 | 1.6288 | 15.336 | 0.4034 | 47.455 |  |
| 127 | 0.9835 | 16.4878 | 0.3773 | 46.9619 |  |
| 138 | 0.8367 | 16.7225 | 0.5511 | 38.9643 |  |
| 149 | 1.6793 | 15.3296 | 0.2742 | 48.4533 |  |
| 160 | 1.8756 | 16.793 | 0.4595 | 38.6554 |  |
|  |  |  |  |  |  |
| ***Data sources indicated in parentheses in column headings.** | | | | |  |

| **Supplementary Table S3. ALL DATA ON 5MYR BINS ONLY NO DATA DIFFERENCED.*** | | | | | |
| --- | --- | --- | --- | --- | --- |
| **Age (Ma)** | **CO_2_ (ppm) (Foster et al.)** | **^87^Sr/^86^Sr (Cárdenas & Harries)** | **Biogenic Phosphorus Accumulation Rate (Föllmi)** | **Total Phosphorus Accumulation Rate (PAR) (Föllmi)** | **Nannofossil Diversification Rate (Rs-Re) (Bown et al.)** |
| 17 | 261 | 0.7087 | 0.13751188 | 0.50177838 | -4.816 |
| 22 | 350.9090909 | 0.708427273 | 0.25875025 | 0.449204114 | -6.592408071 |
| 27 | 440.8181818 | 0.708154545 | 0.379988621 | 0.396629848 | -8.368816142 |
| 32 | 498.630303 | 0.707990909 | 0.309047384 | 0.351206466 | -6.544018131 |
| 37 | 548.4181818 | 0.707854545 | 0.190061245 | 0.307570805 | -3.8189186 |
| 42 | 602.2589531 | 0.7078 | 0.211338775 | 0.351735101 | 0.928747527 |
| 47 | 658.8016529 | 0.7078 | 0.326125415 | 0.454432704 | 7.024791385 |
| 52 | 571.1969222 | 0.707818182 | 0.468698903 | 0.539256646 | 12.82894801 |
| 57 | 267.3710459 | 0.707863636 | 0.65295266 | 0.597270098 | 18.19527378 |
| 62 | 50.05733827 | 0.707890909 | 0.745387924 | 0.601542564 | 19.96844644 |
| 67 | 178.7923055 | 0.707845455 | 0.47054922 | 0.390851082 | 7.369006621 |
| 72 | 307.5272727 | 0.7078 | 0.195710515 | 0.1801596 | -5.230433194 |
| 77 | 859.5603306 | 0.707663636 | 0.267585845 | 0.394252661 | -0.368678147 |
| 82 | 1411.593388 | 0.707527273 | 0.339461174 | 0.608345723 | 4.493076901 |
| 87 | 1362.234604 | 0.707463636 | 0.398462855 | 0.645248097 | 2.735872773 |
| 92 | 1162.527859 | 0.707418182 | 0.454246123 | 0.637852799 | -0.676071149 |
| 97 | 1135.462215 | 0.7074 |  | 0.528414254 | -0.610554076 |
| 102 | 1223.490638 | 0.7074 |  | 0.350946876 | 1.773270328 |
| 107 | 1238.979021 | 0.707381818 |  | 0.292441849 | 3.912751222 |
| 112 | 1145.657343 | 0.707336364 |  | 0.412380347 | 5.685716852 |
| 117 | 1118.747475 | 0.707309091 |  | 0.490699333 | 6.109876157 |
| 122 | 1357.484848 | 0.707354545 |  | 0.402540274 | 1.138810165 |
| 127 | 1596.222222 | 0.7074 |  | 0.314381215 | -3.832255828 |
| 132 | 953.8379139 | 0.7074 |  | 0.420370565 | 3.946867994 |
| 137 | 311.4536058 | 0.7074 |  | 0.526359915 | 11.72599182 |
| 142 | 386.5176684 | 0.707327273 |  | 0.490991623 | 9.835872151 |
| 147 | 640.9438236 | 0.707236364 |  | 0.420283921 | 5.528441615 |
| 152 | 701.0876623 | 0.707145455 |  |  | 4.051302921 |
| 157 | 631.7099567 | 0.707054545 |  |  | 4.461025454 |
|  |  |  |  |  |  |

| **Supplementary Table S3. continued** | | |  |  |  |
| --- | --- | --- | --- | --- | --- |
| **Age (Ma)** | **Nannofossil Diversification Rate (Rs-Re) (Bown et al.)** | **δ^13^C (Cárdenas & Harries)** | **δ^34^S (Cárdenas & Harries)** | **Origination Rate (Cárdenas & Harries)** | **Sea Level (Cárdenas & Harries)** |
| 17 | -4.816 | 2.0508 | 22.156 | 0.2543 | 45.7793 |
| 22 | -6.592408071 | 1.801254545 | 22.08509091 | 0.261527273 | 47.94261818 |
| 27 | -8.368816142 | 1.551709091 | 22.01418182 | 0.268754545 | 50.10593636 |
| 32 | -6.544018131 | 1.571218182 | 21.94152727 | 0.246927273 | 52.54489091 |
| 37 | -3.8189186 | 1.657990909 | 21.86843636 | 0.217836364 | 55.05275455 |
| 42 | 0.928747527 | 1.947590909 | 21.05579091 | 0.2401 | 60.18954545 |
| 47 | 7.024791385 | 2.372409091 | 19.75010909 | 0.2966 | 67.07895455 |
| 52 | 12.82894801 | 2.548318182 | 18.82515455 | 0.411263636 | 70.29930909 |
| 57 | 18.19527378 | 2.350863636 | 18.47129091 | 0.613172727 | 68.01608182 |
| 62 | 19.96844644 | 2.141690909 | 18.20337273 | 0.726645455 | 65.34297273 |
| 67 | 7.369006621 | 1.885645455 | 18.27923636 | 0.486372727 | 61.11033636 |
| 72 | -5.230433194 | 1.6296 | 18.3551 | 0.2461 | 56.8777 |
| 77 | -0.368678147 | 1.572327273 | 18.14823636 | 0.284054545 | 59.14479091 |
| 82 | 4.493076901 | 1.515054545 | 17.94137273 | 0.322009091 | 61.41188182 |
| 87 | 2.735872773 | 1.441636364 | 17.42363636 | 0.324181818 | 63.86777273 |
| 92 | -0.676071149 | 1.364181818 | 16.82818182 | 0.317409091 | 66.37086364 |
| 97 | -0.610554076 | 1.705527273 | 16.02 | 0.328636364 | 62.57335455 |
| 102 | 1.773270328 | 2.326072727 | 15.07 | 0.351863636 | 54.57544545 |
| 107 | 3.912751222 | 2.503927273 | 14.652 | 0.372636364 | 49.35457273 |
| 112 | 5.685716852 | 2.017745455 | 15.032 | 0.389727273 | 48.29925455 |
| 117 | 6.109876157 | 1.570136364 | 15.44070909 | 0.401027273 | 47.41017273 |
| 122 | 1.138810165 | 1.276818182 | 15.96425455 | 0.389163636 | 47.18603636 |
| 127 | -3.832255828 | 0.9835 | 16.4878 | 0.3773 | 46.9619 |
| 132 | 3.946867994 | 0.916772727 | 16.59448182 | 0.4563 | 43.32662727 |
| 137 | 11.72599182 | 0.850045455 | 16.70116364 | 0.5353 | 39.69135455 |
| 142 | 9.835872151 | 1.1431 | 16.21599091 | 0.450409091 | 42.41484545 |
| 147 | 5.528441615 | 1.5261 | 15.58285455 | 0.324545455 | 46.72802727 |
| 152 | 4.051302921 | 1.732836364 | 15.72870909 | 0.324736364 | 45.78114545 |
| 157 | 4.461025454 | 1.822063636 | 16.39389091 | 0.408963636 | 41.32755455 |
|  |  |  |  |  |  |
| ***Data sources indicated in parentheses in column headings.** | | | |  |  |

| **Supplementary Table S4. 11 MYR DATA BINNED ON EXACT 11MYR AND DIFFERENCED.*** | | | | | |
| --- | --- | --- | --- | --- | --- |
| **Age (Ma)** | **CO_2_ (Foster et al.)** | **Sr (Cárdenas & Harries)** | **Phosphorus Accumulation Rate (BIOGENIC) (Föllmi)** | **Phosphorus Accumulation Rate (TOTAL) (Föllmi)** | **Nannofossil Diversification Rate** |
| 17 | -458.8 | -0.7081 | -0.404236295 | -0.386114995 | 8.724097756 |
| 28 | -307.3333333 | 0.0009 | -0.00495491 | 0.21166184 | -2.087121212 |
| 39 | -233.9272727 | 0.0003 | 0.009238895 | -0.12993627 | -19.40651546 |
| 50 | 544.0229885 | -1.00E-04 | -0.657888875 | -0.35356432 | -25.21721319 |
| 61 | 385.2 | 0 | 0.199286885 | 0.335891665 | 15.91285089 |
| 72 | -1497.689655 | 0.0004 | 0.446519425 | -0.007483475 | 17.02290649 |
| 83 | -775.1178883 | 0.0004 | -0.280848915 | -0.45473508 | -3.189584476 |
| 94 | 245.692308 | 1.00E-04 | 0.35383624 | 0.406697885 | 2.26186294 |
| 105 | 11.645161 | 1.00E-04 |  | 0.126563535 | -9.144938074 |
| 116 | -319.91453 | 0 |  | -0.069914765 | 7.035820798 |
| 127 | 888.0232558 | -1.00E-04 |  | -0.03922664 | -6.177727224 |
| 138 | 853.5079363 | 0.0002 |  | -0.077619625 | -7.637725229 |
| 149 | -407.1065891 | 0.0004 |  | 0.547557785 | 8.574957606 |
| 160 | -7.7857143 | 0.7072 |  |  | 3.805469401 |

| **Supplementary Table S4. continued** | | |  |  |
| --- | --- | --- | --- | --- |
| **Age (Ma)** | **d^13^C (Cárdenas & Harries)** | **d^34^S (Cárdenas & Harries)** | **Origination Rate (Cárdenas & Harries)** | **Sea Level (Cárdenas & Harries)** |
| 17 | -1.5018 | -22 | -0.2702 | -50.5386 |
| 28 | 0.3581 | 0.3168 | 0.0481 | -10.2766 |
| 39 | -1.1255 | 3.0333 | -0.0603 | -20.674 |
| 50 | -0.5002 | 3.651 | -0.5685 | -10.1336 |
| 61 | 0.9977 | 0.6116 | 0.0844 | 14.3349 |
| 72 | 0.6893 | 0.2882 | 0.4451 | 4.3242 |
| 83 | 0.2964 | 1.7651 | -0.0686 | -10.4944 |
| 94 | -1.1948 | 3.4 | -0.0362 | 12.0886 |
| 105 | -0.2956 | 1.254 | -0.0887 | 19.9171 |
| 116 | 1.7149 | -1.9878 | -0.0115 | 2.8148 |
| 127 | 0.7921 | -1.3865 | -0.1477 | 8.4907 |
| 138 | -0.6958 | 1.1582 | 0.1031 | -1.4914 |
| 149 | -1.0389 | -0.0705 | 0.0916 | 0.3089 |
| 160 | 1.6793 | 15.3296 | 0.2742 | 48.4533 |
|  |  |  |  |  |
| ***Data sources indicated in parentheses in column headings.** | | | |  |

| **Supplementary Table S5. 5 MYR DATA BINNED ON EXACT 5 MYR AND DIFFERENCED.*** | | | | | |
| --- | --- | --- | --- | --- | --- |
| **Age (Ma)** | **CO_2_ (ppm) (Foster et al.)** | **^87^Sr/^86^Sr (Cárdenas & Harries)** | **Biogenic Phosphorus Accumulation Rate (Föllmi)** | **Total Phosphorus Accumulation Rate (PAR) (Föllmi)** | **Nannofossil Diversification Rate (Rs-Re) (Bown et al.)** |
| 17 | -350.9090909 | -0.708427273 | -0.25875025 | -0.449204114 | 6.592408071 |
| 22 | -179.8181818 | 0.000545455 | -0.242476741 | 0.105148532 | 3.552816142 |
| 27 | -147.7212121 | 0.000436364 | -0.050297134 | 0.097997648 | -0.04838994 |
| 32 | -107.6 | 0.0003 | 0.189927375 | 0.089059044 | -4.549897541 |
| 37 | -103.6286501 | 0.000190909 | 0.09770861 | -0.000528635 | -7.472765658 |
| 42 | -110.3834711 | 5.45E-05 | -0.13606417 | -0.146861899 | -10.84370999 |
| 47 | 31.06203096 | -1.82E-05 | -0.257360128 | -0.187521545 | -11.90020048 |
| 52 | 391.430607 | -6.36E-05 | -0.326827244 | -0.142837395 | -11.1704824 |
| 57 | 521.1395839 | -7.27E-05 | -0.276689021 | -0.062285918 | -7.139498428 |
| 62 | 88.57874039 | 1.82E-05 | 0.18240344 | 0.206419016 | 10.82626716 |
| 67 | -257.4699344 | 9.09E-05 | 0.549677409 | 0.421382964 | 25.19887963 |
| 72 | -680.7680251 | 0.000181818 | 0.202963375 | -0.00340158 | 7.737684768 |
| 77 | -1104.066116 | 0.000272727 | -0.143750659 | -0.428186123 | -9.723510095 |
| 82 | -502.6742734 | 0.0002 | -0.13087701 | -0.250995435 | -3.104550919 |
| 87 | 249.0655294 | 0.000109091 | -0.114784949 | -0.029507076 | 5.16914805 |
| 92 | 226.7723891 | 6.36E-05 | 0.398462855 | 0.116833843 | 3.346426848 |
| 97 | -60.96277909 | 1.82E-05 |  | 0.286905923 | -2.449341477 |
| 102 | -103.5168058 | 1.82E-05 |  | 0.235972405 | -4.523305298 |
| 107 | 77.83329555 | 6.36E-05 |  | -0.06143347 | -3.912446524 |
| 112 | 120.231546 | 7.27E-05 |  | -0.198257484 | -2.197124935 |
| 117 | -211.8275058 | -1.82E-05 |  | 0.009840073 | 4.546906687 |
| 122 | -477.4747473 | -9.09E-05 |  | 0.176318118 | 9.942131985 |
| 127 | 403.6469345 | -4.55E-05 |  | -0.017830291 | -2.808057829 |
| 132 | 1284.768616 | 0 |  | -0.2119787 | -15.55824764 |
| 137 | 567.3202455 | 7.27E-05 |  | -0.070621058 | -5.889004157 |
| 142 | -329.4902178 | 0.000163636 |  | 0.106075994 | 6.1975502 |
| 147 | -314.5699939 | 0.000181818 |  | 0.490991623 | 5.78456923 |
| 152 | 9.233866927 | 0.000181818 |  |  | 1.067416161 |
| 157 | 81.83766235 | 0.707145455 |  |  | 4.051302921 |

| **Supplementary Table S5. continued** | | |  |  |
| --- | --- | --- | --- | --- |
|  |  |  |  |  |
| **Age (Ma)** | **δ^13^C (Cárdenas & Harries)** | **δ^34^S (Cárdenas & Harries)** | **Origination Rate (Cárdenas & Harries)** | **Sea Level (Cárdenas & Harries)** |
| 17 | -1.801254545 | -22.08509091 | -0.261527273 | -47.94261818 |
| 22 | 0.499090909 | 0.141818182 | -0.014454545 | -4.326636364 |
| 27 | 0.230036364 | 0.143563636 | 0.0146 | -4.602272727 |
| 32 | -0.106281818 | 0.145745455 | 0.050918182 | -4.946818182 |
| 37 | -0.376372727 | 0.885736364 | 0.006827273 | -7.644654545 |
| 42 | -0.714418182 | 2.118327273 | -0.078763636 | -12.0262 |
| 47 | -0.600727273 | 2.230636364 | -0.171163636 | -10.10976364 |
| 52 | 0.021545455 | 1.278818182 | -0.316572727 | -0.937127273 |
| 57 | 0.406627273 | 0.621781818 | -0.315381818 | 4.956336364 |
| 62 | 0.465218182 | 0.192054545 | 0.1268 | 6.905745455 |
| 67 | 0.512090909 | -0.151727273 | 0.480545455 | 8.465272727 |
| 72 | 0.313318182 | 0.131 | 0.202318182 | 1.965545455 |
| 77 | 0.114545455 | 0.413727273 | -0.075909091 | -4.534181818 |
| 82 | 0.130690909 | 0.7246 | -0.040127273 | -4.722981818 |
| 87 | 0.150872727 | 1.113190909 | 0.0046 | -4.958981818 |
| 92 | -0.263890909 | 1.403636364 | -0.004454545 | 1.294418182 |
| 97 | -0.961890909 | 1.758181818 | -0.034454545 | 11.79541818 |
| 102 | -0.7984 | 1.368 | -0.044 | 13.21878182 |
| 107 | 0.308327273 | 0.038 | -0.037863636 | 6.276190909 |
| 112 | 0.933790909 | -0.788709091 | -0.028390909 | 1.9444 |
| 117 | 0.740927273 | -0.932254545 | 0.000563636 | 1.113218182 |
| 122 | 0.586636364 | -1.047090909 | 0.023727273 | 0.448272727 |
| 127 | 0.360045455 | -0.630227273 | -0.067136364 | 3.859409091 |
| 132 | 0.133454545 | -0.213363636 | -0.158 | 7.270545455 |
| 137 | -0.226327273 | 0.378490909 | 0.005890909 | 0.911781818 |
| 142 | -0.676054545 | 1.118309091 | 0.210754545 | -7.036672727 |
| 147 | -0.589736364 | 0.487281818 | 0.125672727 | -3.3663 |
| 152 | -0.295963636 | -0.811036364 | -0.084418182 | 5.400472727 |
| 157 | 1.732836364 | 15.72870909 | 0.324736364 | 45.78114545 |
|  |  |  |  |  |
| ***Data sources indicated in parentheses in column headings.** | | | |  |
